# EruA, a Regulator of Adherent-Invasive *E. coli,* Enhances Bacterial Pathogenicity by Promoting Adhesion to Epithelial Cells and Survival Within Macrophages

**DOI:** 10.3390/biom16010152

**Published:** 2026-01-14

**Authors:** Zeyan Xu, Chuyu Qin, Ruohan Zhang, Mengting Wu, Anqi Cui, Wei Chen, Lu Chen, Daqing Gao, Ruihua Shi

**Affiliations:** 1 School of Medicine, Southeast University, Ding Jiaqiao Street 87, Nanjing 210009, China; xzy201036@hotmail.com (Z.X.);; 2 Department of Gastroenterology, Affiliated Zhongda Hospital, Southeast University, Ding Jiaqiao Street 87, Nanjing 210009, China

**Keywords:** adherent-invasive *Escherichia coli*, *eruA* gene, regulator, bacterial resistance, bacterial pathogenesis, inflammatory bowel disease

## Abstract

Adherent-invasive *E. coli* (AIEC) is closely related to inflammatory bowel disease (IBD). However, its pathogenic mechanism has not yet been fully elucidated. Using a BLASTP search, we discovered that the amino acid sequence of a putative protein (UFP37798.1) in the AIEC LF82 strain is highly homologous to some regulators in the SlyA family. We named it EruA. We displayed the secondary structures of EruA using bioinformatics, overexpressed the His_6_-tagged EruA protein using SDS-PAGE, and dissected the genetic organization of the *eruA* chromosomal region using 5′RACE. We constructed an *eruA* deletion mutant (ΔeruA) and a complementary strain (CΔeruA) of the LF82 strain. The transcriptomes of wild-type (WT) and ΔeruA bacteria were compared using RNA sequencing and qRT-PCR, thereby identifying 32 differentially expressed genes (DEGs). Based on YASARA software and EMSA analysis, EruA directly binds to the consensus sequences (P_fimA_ and P_tnaB_) in the promoter region of the *fimA* and *tnaB* genes from these DEGs. By using a super-resolution confocal microscope (SCM), counting CFUs of colonies on plates, indole quantification, and crystal violet staining of biofilms adhered to tubes or 96-well plates, we found that EruA activates the *fimA* to promote bacterial adhesion to intestinal epithelial cells and activates the *tnaB* to enhance bacterial indole production and biofilm formation. Moreover, EruA helps AIEC resist environmental stress and enhances bacterial survival within macrophages as well as loading in mouse tissues. Notably, EruA promotes AIEC colonization in the colons of mice and exacerbates intestinal inflammation caused by bacterial infection in mice with DSS-induced inflammatory colitis, manifested by weight loss, colon length shortening, and pathological changes in colon tissues. Therefore, EruA plays a key role in the pathogenicity of AIEC.

## 1. Introduction

Inflammatory bowel disease (IBD), including Crohn’s disease (CD) and ulcerative colitis (UC), affects over a million people worldwide [1]. It has been hypothesized that genetically defective hosts respond to gut microbes, resulting in an inappropriate inflammatory response leading to IBD [2]. Adherent-invasive *Escherichia coli* (AIEC) is considered to be closely involved in the pathogenesis of IBD [3]. The pathogen is frequently isolated in the intestinal mucosa or mucosa-associated tissues of patients with CD [4]. Though no specific genetic signature has been identified in the genome of AIEC [5], its pathogenicity is rather subtle and complex, involving both the pathogen and the host. As its name suggests, AIEC possesses the ability to adhere to and invade intestinal epithelial cells [6]. For example, a carcinoembryonic antigen-associated cell adhesion molecule (ceacam) 6 is found to be overexpressed in the ileal mucosa of patients with CD, and a type I fimbria of AIEC interacts with the receptor of the ceacam 6 in the ileal epithelium, making it easier for the pathogen to adhere to the intestinal epithelial cells [7]. The fimbria on the bacterial surface is assembled from subunits, of which FimA is the main subunit, accounting for 95% of the total subunits. Moreover, the type I fimbria is one of the key structures for the formation of biofilm in bacteria such as *Escherichia coli* (*E. coli*) [8]. OmpR is a transcriptional regulator, and it is essential for the colonization of AIEC and bacterial tolerance to sodium deoxycholate [9]. In addition, another feature of AIEC that has been reported is that the pathogen is able to survive and replicate in phagolysosomes within macrophages [10]. When macrophages are infected by bacteria, the cells will produce reactive oxygen species (ROS) to eliminate the bacteria. In order to survive, AIEC has to adapt to the oxidative and acidic environment within macrophages [11]. A gene encoding an invasive brain endothelial protein A (IbeA) in AIEC facilitates the invasion of the pathogen into intestinal epithelial cells and enables its replication in macrophages [12]. AIEC assembles biofilm-like communities to protect the pathogen from various pressures within macrophages. RstA/RstB is a two-component system (TCS) that is required for the formation of biofilms in AIEC and helps the bacteria resist acidic stress and replicate within macrophages [13]. In addition, a tryptophanase (TnaA) in *E. coli* decomposes tryptophan to produce indole, and the production of indole is mainly dependent on a tryptophan-specific transporter (TnaB) [14]. Indole is a type of signaling molecule that regulates bacterial resistance, biofilm formation, and survival in host cells [15].

In addition, as AIEC has to adapt to oxidative and acidic environments within macrophages, transcriptional regulators are of great significance for the pathogen’s response to these environmental stresses. SlyA is a regulator in *Salmonella typhimurium* (*S. typhimurium*). It is a crucial regulatory factor that can increase bacterial metabolic activity and survival ability within macrophages, and it also contributes to the bacterial virulence [16,17]. However, SlyA in *Enterococcus faecalis* reduced bacterial persistence, survival, and virulence in an insect infection model [18]. Moreover, SlyA-like proteins are present in 118 species of bacteria, and in *E. coli* alone, there are 16 homologous proteins [19]. Members of the SlyA family include Rap, Hor, RovA, and Eha proteins from *Serratia*, *Erwinia*, *Yersinia,* and *Edwardsiella*, all of which are bacterial transcription factors [20]. The family uncovers a conserved architecture, a helix-turn-helix (HTH) domain, which connects a winged region with an α-helical dimerization domain [21]. The active form of SlyA exists in the form of a dimer in vitro [22]. We found that a putative protein (UFP37798.1) in the AIEC LF82 strain shared 92% homology with SlyA in the *Salmonella enterica* (*S. enterica*) subsp. *indica* strain. However, there are currently no reports on proteins similar to SlyA in AIEC.

In the present study, the secondary structure of the protein (UFP37798.1) was revealed based on bioinformatics analysis. The His_6_-tagged protein was overexpressed, as confirmed by SDS-PAGE analysis. The gene organization of the open reading frame (ORF) chromosomal region was dissected on the basis of 5′ rapid amplification of cDNA ends (5′RACE) analysis. We considered this ORF to be a gene and named it *E. coli* LF82 regulating gene (*eruA*) (GenBank accession number: MZ475355). To further demonstrate the function of the *eruA* gene, we constructed an eruA deletion mutant (ΔeruA) as well as a complementary strain (CΔeruA) of the AIEC LF82 strain. Under certain conditions, the transcriptomes of ΔeruA bacteria and wild-type (WT) bacteria were compared to identify some differentially expressed genes (DEGs). The main interaction sites between the consensus sequences in the promoters of the target genes from these DEGs and EruA were analyzed by bioinformatics methods. The results were verified by electrophoretic mobility shift assay (EMSA). Next, to gather evidence for the role of EruA in AIEC biology, we compared the biological effects of the WT and ΔeruA bacteria according to the function of their target genes. Finally, we found that EruA can enhance the bacterial survival ability within macrophages as well as the loading in mouse tissues. EruA promoted AIEC colonization in the colons of mice and exacerbated intestinal inflammation caused by bacterial infection in mice with DSS-induced inflammatory colitis, manifested by weight loss, colon length shortening, and pathological changes in colon tissues. Our work contributes to the understanding of the function of regulator EruA in AIEC, particularly its role in bacterial pathogenicity, which may influence the management of IBD.

## 2. Materials and Methods

### 2.1. Strains, Cells, and Growth Conditions

The AIEC LF82 strain was kindly provided by Prof. Moller-Jensen [23]. Plasmid pDS132 and *E. coli* SM10λpir were kindly supplied by Prof. Liang [24]. The plasmids and bacterial strains used in the study are listed in Table 1. All bacteria were grown in Luria-Bertani (LB) (Sunshine Biotechnology, Nanjing, China). Murine RAW 264.7 macrophage (CBP60533) and murine intestinal epithelial MODE-K cells (C495) were obtained from Nanjing Cobioer Biosciences Co., Ltd. (Nanjing, China). Human monocyte THP-1 cells (BH-C136) were obtained from Bohui Biotechnolog Co., Ltd. (Guangzhou, China). The RAW 264.7 macrophages and MODE-K cells were respectively cultured in Dulbecco’s Minimal Eagle Medium (DMEM; Gibco, Shanghai, China) and Roswell Park Memorial Institute (PRMI) 1640, supplemented with 10% (*v*/*v*) fetal calf serum (FCS) (Gibco, Shanghai, China) at 37 °C in a humidified incubator with less than 5% CO_2_.

THP-1 cells were grown in suspension with RPMI 1640, with glutamax supplemented with 10% FCS in the presence of phorbol 12-myristate-12 acetate (PMA; Sigma-Aldrich, St. Louis, MO, USA). The PMA was moved after two days of treatment.

### 2.2. Bioinformatic Analysis of EruA Structure

The amino acid sequence of EruA from the AIEC LF82 strain was compared with that of SlyA from *Salmonella enterica* subsp. *indica* using BLASTP (https://blast.ncbi.nlm.nih.gov/Blast.cgi, accessed on 31 October 2026). Multiple sequence alignment was performed and optimized using Jalview software (version 2.11.3.0) for comparative analysis. Structural models of the EruA monomer and dimer were predicted using the AlphaFold Server (AlphaFold3, https://alphafoldserver.com/) with default parameters. To model the EruA-DNA interaction, the experimentally determined structure of the SlyA-DNA complex was used as a structural reference, and protein-double-stranded DNA complex prediction was performed using AlphaFold3.

Predicted models were evaluated based on established confidence metrics, including the interface predicted TM-score (ipTM). Models with an ipTM score ≥ 0.8 were considered high-confidence predictions and selected for downstream analyses, whereas models with ipTM scores < 0.6 were excluded due to insufficient reliability. Selected models were subsequently visualized and rendered using PyMOL software (version 3.0.3). Further optimization of hydrogen-bond networks and analysis of protein–DNA intermolecular interactions were conducted using YASARA (version 19.9.17). Protein–DNA interface residues and non-covalent interactions were identified using the Analyze > Interactions module with a distance cut-off of ≤4.0 Å.

### 2.3. 5′RACE Analysis of the eruA Gene Chromosomal Region

The identification of 5′ ends of the *eruA* transcripts was performed by the 5′RACE system (Sangon, Shanghai, China), as described in the manufacturer’s instructions. Total RNA was extracted from the AIEC LF82 strain, and cDNA was synthesized based on reverse transcription (RT) with the RC1145-RT3 and RC1145-RT4 primers (Table 2). In the middle amplification step, PCR was performed with primers RC1145-MZ-Y-F and RC1145-MZ-Y-R (Table 2), and the products were sequenced. For nested amplification, the first-round PCR was finished using the RC1145-R3 and 5′ adaptor primers, and the second-round PCR used the primers RC1145-R4 and 5.3′ outer primers (Table 2). Subsequently, sequencing products were carried out.

### 2.4. Cloning the eruA Gene

The X-F and X-R primers (Table 2) from the fragment were designed according to the genomic sequence of the AIEC LF82 strain (GenBank accession no: NC_011993). The fragment was amplified from the AIEC LF82 strain by PCR and sequencing. A 435 bp gene was digested with *EcoR I* and *Hind Ⅲ* and inserted into a pET28a^+^ vector with His_6_-tag at its N-terminus to produce a recombinant plasmid, pET28a-eruA. The recombinant plasmid and vector plasmid were respectively transformed into *E. coli* BL21 using the CaCl_2_ method. *E. coli* pET28a-eruA/BL21 (DE3) and pET28a^+^/BL21 (DE3) were obtained on plates containing kanamycin (50 μg/mL).

### 2.5. Expression and Purification of an EruA Protein in E. coli

The logarithmic phase pET28a-eruA/BL21 and pET28a^+^/BL21 strains were separately cultured in LB with kanamycin (50 μg/mL) at 37 °C until the OD_600_ reached 0.5. Next, a final concentration of 1 mM isopropyl β-D-1-thiogalactopyranosid (IPTG, Sigma-Aldrich, St. Louis, MO, USA) was added to induce protein production for 4 or 6 h at 30 °C. After these cultures were centrifuged, bacterial pellets were collected and dispersed in phosphate buffer saline (PBS). These bacterial suspensions were broken by ultrasonication on ice for 10 min at 400 W, and were centrifuged.

Next, a 200 mL logarithmic phase culture of the pET28a-eruA/BL21 was induced with 1 mM IPTG for 6 h at 30 °C. The bacterial pellets were harvested after centrifugation and dispersed in TieChui *E. coli* lysis buffer (ACE, Shuzhou, China) on ice for 30 min, and lysed by ultrasonication on ice for 10 min at 400 W, and then centrifuged. A His_6_-tagged EruA fusion protein was purified by affinity chromatography, following the manufacturer’s instructions for Protino NI-NTA Agarose (Gene Biotech, Shanghai, China), as described by Spriestersbach et al. [25].

The proteins from the supernatants were quantified with a bicinchoninic acid (BCA) protein assay kit (Yeasen, Shanghai, China) and separated by 12% sodium dodecyl sulfate-polyacrylamide gel electrophoresis (SDS-PAGE). The gels were stained with Coomassie brilliant blue.

### 2.6. Constructing an eruA Mutant (∆eruA), Complementary Strain, and LF 82-flu Strain

The mutant was constructed as described by Gao et al. [26], with minor modifications. In brief, a synthesized F1F2 fragment (Genewiz, Suzhou, China) was generated that encompassed the upstream and downstream regions of the *eruA* gene from the AIEC LF82 strain. The F1F2 fragment was ligated into a recombinant plasmid pDS132-F1F2 using a suicide vector pDS132. The recombinant plasmid was transformed into *E. coli* SM10λpir using the CaCl_2_ method, and then introduced into the AIEC LF82 strain by conjugation with SM10λpir. The conjugants (pDS132-F1F2/LF82 strain) were selected on LB plates containing 30 μg/mL chloramphenicol and 100 μg/mL ampicillin. An eruA mutant was obtained by selection on LB plates with 15% wt/vol sucrose and 30 μg/mL chloramphenicol. The eruA mutant was identified by PCR with F1-F/F2-R primers (Table 2) and was confirmed by sequencing (Sunshine, Nanjing, China). The eruA mutant was named ∆eruA.

The tnaB and fimA fragments were respectively amplified from the AIEC LF82 strain by PCR with the tnaB-F, tnaB-R, fimA-F, and fimA-R primers (Table 2) and inserted into the pET28a^+^ vector to produce two recombinant plasmids, pET28a-tnaB and pET28a-fimA. The recombinant plasmids, pACYC184-eruA, pET28a-tnaB, and pET28a-fimA, were transformed into ΔeruA bacteria to generate the complementary strains CΔeruA, ΔeruA-tnaB, and ΔeruA-fimA using the CaCL_2_ method. These complementary strains were confirmed by PCR with eruA-F, eruA-R, tnaB-F, tnaB-R, fimA-F, and fimA-R primers, respectively (Table 2).

A red fluorescence plasmid, pBBbr1mcs2-Tac-mcherry, was transformed into the wild type (WT), ΔeruA, CΔeruA, and ΔeruA-fimA of the LF 82 strain to produce the WT-flu, ΔeruA-flu, CΔeruA-flu, and ΔeruA-fimA-flu strain using the CaCl_2_ method.

### 2.7. RNA Extraction and Transcriptome Sequencing

The logarithmic phase WT and ΔeruA bacteria continued to grow in LB broth (pH 6.3) for 2 h at 37 °C. The two types of RNA were isolated using an RNA extraction kit (TaKaRa Bio, Dalian, China). The concentration, purity, and RIN (RNA integrity number) of these RNAs were inspected with Nanodrop 2000 spectrophotometer (Thermo Fisher Scientific, Wilmington, DE, USA) and Agilent 2100 (Agilent Technologies, Santa Clara, CA, USA). The complementary DNA (cDNA) libraries were constructed. These samples were sequenced on the Illumina sequencing 2000 platform located in Shenzhen Hua Da Gene Co., Ltd. (Shenzhen, China). The raw data from RNA-sequencing were deposited in the NCBI SRA (Sequence Read Archive) database (BioProject number: PRJNA1070331).

### 2.8. Analysis of Differentially Expressed Genes (DEGs)

Clean reads were mapped onto the genome sequences of the reference AIEC LF82 strain (Genbank accession number: NC011993.1). The quality of transcriptome sequencing was evaluated. The differences in the gene expression between the WT and ΔeruA bacteria were compared, and the mRNA abundances were estimated using the expected number of fragments per kilobase of transcript sequenced per million base pairs sequenced (RPKM) method. If the differences in RPKM values of these genes between the two samples were |log2 fold change| ≥ 2, false discovery rate (FDR) ≤ 0.001, and *p*-value < 0.05, the genes were considered to be DEGs. The FDR adjusted the *p*-value. The functional classification of these DEGs was analyzed with Gene Ontology (GO).

### 2.9. Binding of the Consensus Sequences (P_fimA_ or P_tnaB_) in the Promoter Region of fimA or tnaB Genes by EruA Detected by EMSA

For EMSA, oligonucleotide probes (PfimA, uPfimA, mPfimA, PtnaB, uPtnaB, and mPtnaB, Table 2) were synthesized in GenScript Biotech Co., Ltd. (Nanjing, China) using the conserved sequences in the promoter region of the *fimA* and *tnaB* genes and labeled or unlabeled with fluorescent CY3 at the 5′ end, and annealed. Experiments were performed in accordance with the manufacturer’s instructions for the LightShift™ Chemiluminescent EMSA optimization and control kit (Thermo Fisher Scientific Inc., Waltham, MA, USA). The purified EruA protein was bound to 10 nM probes in EMSA buffer for 20 min at room temperature. The probe–EruA protein binding complexes were separated on 6% native polyacrylamide gels using a 0.5× Tris-Borate-EDTA (TBE) buffer system for 1.5 h at 4 °C. The resulting bands were detected using a Sapphire™ FL biomolecular imager (Azure Biosystems, Dublin, CA, USA) and analyzed with Sapphire Capture software (version 18.5).

### 2.10. Counting Colony-Forming Units (CFUs) of Colonies on Plates and Observing Under a Super-Resolution Confocal Microscope (SCM) for Adhesion to Cells

A MODE-K cell suspension (about 1 × 10^5^ cell/mL) was added to a sterile coverslip in each well on a 24-well plate and incubated in RPMI with 10% FCS at 37 °C for 24 h. After log-phase bacterial suspensions (about 1 × 10^8^ CFU/mL) were centrifuged, bacterial precipitates were washed with PBS and resuspended with 1640 containing 50 μg/mL kanamycin, either with or without 35 μg/mL chloramphenicol. After the MODE-K cells were infected by the WT-flu, ΔeruA-flu, or ΔeruA-fimA-flu bacteria for 20 min or 2 h at a multiplicity of infection (MOI) of 100:1 and were washed with PBS, the cells were lysed with 0.2% Triton X-100. Samples were diluted and plated on LB plates, either with or without 35 μg/mL chloramphenicol, overnight at 37 °C to count CFUs/mL. The CFUs/mL were determined as CFUs/mL × dilution ratio.

Then, the infected cells were permeabilized with 0.2% Triton X-100, washed with PBS, and incubated with EPCAM/CD326 goat anti-mouse polyclonal antibody (Proteintech, Wuhan, China) and Alexa Fluor 488-conjugated goat anti-rabbit polyclonal antibody (Servicebio, Wuhan, China) for 1 h at room temperature. Finally, the coverslips were mounted using fluoroshield^TM^ with DAPI (Sigma-Aldrich, St. Louis, MO, USA).

Images were captured using an SCM (Nikon A1R HD25, Tokyo, Japan) equipped with 100× oil-immersion objective, analyzed with an NIS Elements Viewer 5.21, and formatted using Image J software (version 1.0).

### 2.11. Determination of Indole Concentrations in Bacterial Cultures Using a p-Dimethylaminobenzaldehyde (p-DMAB)

This assay was performed as described previously [27]. First, an indole standard curve indicating the relationship between different concentrations of indole standard (Sinopharm, Shanghai, China) and the corresponding spectrophotometric absorbances (OD_571_) was established. Next, overnight, 2 mL bacterial cultures were sub-cultured into 200 mL LB broth, either without or with 35 μg/mL chloramphenicol at 37 °C. Five milliliters of bacterial culture were removed every 2 h from 2 to 18 h, and at 24 and 36 h, and centrifuged at 4000 rpm for 5 min. Then, the supernatants were filtered and added to the 200 µL p-DMAB developer. These mixtures were incubated in a boiling water bath for 10 min. The absorbance at OD_571_ was measured by an ultraviolet spectrophotometer. The relative indole concentrations were calculated from the indole standard curve.

### 2.12. Biofilm Assays with Crystal Violet Staining

Bacterial biofilm assays were stained with crystal violet, as previously described [27]. Five microliters of fresh bacterial suspension (about 1 × 10^8^ CFU/mL) was added to 5 mL LB in a glass tube and incubated at 37 °C for designated times (24, 36, 48, 60, or 72 h). After discarding these cultures, the tubes were washed with PBS three times and stained with 1% crystal violet for 25 min. After the staining solutions were discarded, the tubes were washed with sterile water for 2 min three times. At the junction of culture and air, the purple biofilms were observed in the walls of the tubes.

Subsequently, 250 microliters of bacterial suspension (about 1 × 10^8^ CFU/mL) were added to each well in a 96-well plate, and the suspensions were incubated for 48 h at 37 °C, with LB medium serving as a negative control. After discarding the cultures, the wells were fixed with 250 microliters of methanol and washed with PBS. The wells were stained with 1% crystal violet, and decolorized with 250 μL acetic acid for 25 min. The absorbance value at OD_570_ in each well was determined by an automatic microplate reader.

### 2.13. Bacterial Survival Rate in H_2_O_2_ or Acidic LB Medium

As previously described [26], logarithmic phase bacteria were cultured in LB broth with various concentrations of H_2_O_2_ (2, 5, or 10 mM), or in LB broth (pH 2.0 or 5.8) for 2 h at 37 °C. Bacteria grown in standard LB broth (pH 7.4) without H_2_O_2_ were used as the control group. After the treatment, bacteria were diluted and smeared on LB plates overnight at 37 °C. The bacterial survival rates were determined as the colony numbers in the experimental group/the colony numbers in the control group × 100%.

### 2.14. Bacterial Growth Curve

The fresh bacterial cultures were sub-cultured 1:100 into 200 mL LB broth or DMEM, either with or without 35 μg/mL chloramphenicol at 37 °C. Every hour, one milliliter of bacterial culture was taken out of each culture to measure its OD_600_. The growth curves of LB or DMEM at different times and with corresponding spectrophotometric absorbances (OD_600_) were established.

### 2.15. Intracellular Bacterial Replication Observed Under an SCM

Briefly, RAW 264.7 macrophage suspension (about 1 × 10^5^ cells/mL) was added to a sterile coverslip in each well on a 24-well plate and incubated in DMEM with 10% FCS for 24 h at 37 °C. After the macrophages were infected by the WT-flu, ΔeruA-flu, or CΔeruA-flu bacteria in LB containing 50 μg/mL kanamycin, either with or without 35 μg/mL chloramphenicol for 20 min at a MOI of 100:1, the cells were cultured in DMEM containing 200 μg/mL gentamicin for 1 h to kill the bacteria outside the cells. Then, when these cells were further cultured using a fresh medium containing 30 μg/mL gentamicin, time was defined as 0 h.

Intracellular bacterial replication was observed under an SCM as previously described [13]. At designated times (1, 6, and 18 h) post-infection, the infected cells were washed with PBS and fixed with 4% paraformaldehyde for 10 min. Then, the infected cells were permeabilized with 0.2% Triton X-100 and washed with PBS. The coverslips were mounted using Fluoroshield^TM^ with DAPI (Sigma-Aldrich, St. Louis, MO, USA). Images were captured using an SCM.

### 2.16. Counting CFUs/mL of Colonies on Plates for Intracellular Bacterial Replication

Bacterial intracellular replication in RAW 264.7 macrophages or THP-1 cells was assessed by counting CFUs/mL on plates as previously described [26]. Briefly, the cell suspension (about 1 × 10^5^ cells/mL) was added to each well on a 24-well plate and incubated in DMEM or RPMI with 10% FCS for 24 h at 37 °C. The macrophages were infected by the WT, ΔeruA, or CΔeruA bacteria for 20 min at an MOI of 100:1. The cells were then cultured in DMEM or RPMI containing 200 μg/mL gentamicin for 1 h to kill the bacteria outside the cells, then fresh medium containing 30 μg/mL gentamicin, either with or without 35 μg/mL chloramphenicol, was used to continue incubating the cells and defined as 0 h. At designated time intervals (1, 6, and 18 h) post-infection, the infected cells were lysed with 0.2% Triton X-100, then the cell lysates were diluted and spread on LB agar plates, either with or without 35 μg/mL chloramphenicol. After overnight incubation at 37 °C, CFUs/mL were determined as CFUs × dilution ratio/mL.

### 2.17. RNA Extraction and qRT-PCR

The logarithmic phase WT or ΔeruA bacteria continued to grow in LB broth (pH 6.3) for 2 h at 37 °C. The two types of RNA were isolated using an RNA extraction kit (TaKaRa Bio, Dalian, China). The log-phase WT and ΔeruA bacteria of the AIEC LF82 strain were respectively cultivated in DMEM (pH 5.8 or 7.4) or in DMEM containing H_2_O_2_ (0 or 2 mM) for 2 h at 37 °C. In addition, the WT bacteria infected RAW 264.7 macrophages for 20 min at an MOI of 100:1. The extracellular bacteria were killed with DMEM containing 200 μg/mL gentamicin for 1 h, and defined as 0 h. The cells continued to be cultured in the fresh medium containing 30 μg/mL gentamicin for 1, 6, and 18 h. The WT bacteria grown in DMEM (pH 7.4) served as the control group. After centrifugation, the bacterial deposits were collected and treated with TRIzol reagent (TaKaRa Bio, Dalian, China), and the RNAs were isolated.

RNAs were reverse-transcribed using a PrimeScript™ RT reagent kit (Takara, Dalian). The qPCRs were conducted using SYBR Premix ExTaq (Takara, Dalian, China) in an ABI 7300 real-time detection system (Applied Biosystems, Carlsbad, CA, USA). The primers are shown in Table 2, and the 16S rRNA gene was used as a control. The transcriptional levels of these genes were compared using the 2^−ΔΔCT^ method [28].

### 2.18. Bacterial Loads from Infected Mouse Tissues

All animal experiments were approved by the Institutional Animal Care and Use Committee (IACUC) of Southeast University and were conducted in accordance with the ARRIVE guidelines (version 2.0) [29].

Six-week-old male C57BL/6 mice weighing 18–22 g were obtained from the Experimental Animal Center of Yangzhou University. Mice were raised under specific pathogen-free (SPF) conditions at the Experimental Animal Center of Southeast University. A total of 64 mice were randomly assigned to two experimental groups and intraperitoneally injected with either wild-type (WT) or ΔeruA bacteria.

After 7 d of habituation, each mouse was intraperitoneally injected with 0.2 mL containing 5 × 10^6^ CFUs/mL of logarithmic-phase bacteria suspended in sterile normal saline (NS) as previously described [26]. Briefly, mice were sacrificed under anesthesia at 1, 3, 5, and 7 d post-infection, and organs (liver, kidney, and spleen) were harvested for preparation of tissue homogenates. A series of dilutions of these homogenates were smeared onto LB plates with 100 μg/mL ampicillin. The plates were incubated overnight at 37 °C. The CFUs/organ were determined as CFUs/organ × dilution ratio.

### 2.19. DSS-Induced and Non-Induced Mice Colitis Model

After 7 d of habituation, the mice were randomly divided into Control, DSS, WT, ΔeruA, DSS + ΔeruA, and DSS + WT groups, with 8 mice in each group, using a computer-generated random number sequence. The logarithmic-phage WT or ΔeruA bacteria were suspended in NS (5 × 10^9^ CFUs/mL). The mice in the DSS, DSS + ΔeruA, and DSS + WT groups drank 2.5% (*w*/*v*) DSS (Aladdin Company, Shanghai, China) in distilled water for 5 d to induce colitis, while the mice in the Control, WT, and ΔeruA groups drank NS at the same time. In addition, the mice in the Control and DSS groups were given 200 μL/d distilled water by gavage for 8 d, while the mice in the other groups were given an equal volume of the WT or ΔeruA bacterial suspension at the same time. All mice were administered distilled water for 1 d before sacrifice (Figure 7A). During the experiment, the body weights of the six groups of mice were measured at the same time each day for 9 d. Humane endpoints were predefined: mice exhibiting more than 25% body-weight loss relative to baseline, persistent diarrhea for more than 3 consecutive days, or marked lethargy and anorexia were euthanized and excluded from further analyses. Detailed exclusion criteria and welfare considerations are provided in ARRIVE guidelines 2.0: author checklist.

Before sacrifice on day 17, all the mice in the six groups were anesthetized with isoflurane gas inhalation through a small animal anesthesia machine (RWD Life Science Co., Ltd., Shenzhen, China) and kept in a coma. The mice were anesthetized by inhalation of 4–5% isoflurane, and then 1.5–2% isoflurane was used to maintain anesthesia. Finally, the mice were executed by CO_2_ inhalation after anesthesia.

After the colons were excised, the colon lengths were measured. After removal of the colonic contents, part of the colon tissues were fixed with 4% paraformaldehyde, and the remaining fresh colon tissues were weighed and minced. Then, grinding beads were added to pulverize freshly minced tissues using a tissue crushing instrument. A series of dilutions of the samples were smeared onto LB plates with 100 μg/mL ampicillin. The plates were incubated overnight at 37 °C. The CFUs/g was CFUs × dilution ratio/weight of colon (g).

### 2.20. Hematoxylin and Eosin (H&E) Staining for Histological Analysis

Colonic tissue samples were fixed with 4% paraformaldehyde, embedded with paraffin, and sectioned (4 μm thick). The sections were stained with H&E, and histopathological scores were performed on the basis of the extent of inflammatory cell infiltration (0–5), crypt damage (0–4), ulceration (0–3), and edema (0 or 1) [30]. The parameters were calculated and summed to obtain the total score.

### 2.21. Statistical Analysis

Data are from at least three independent experiments and presented as the mean ± standard deviation (SD). GraphPad Prism (version 8.0) was used to analyze the data. Data from two or more groups were analyzed using a one-way analysis of variance (ANOVA), followed by John Wilder Tukey’s test. The difference was considered to be statistically significant for *p* values < 0.05.

## 3. Results

### 3.1. EruA Resembling SlyA in S. enterica Found in AIEC LF82 Strain

**Figure 1:**
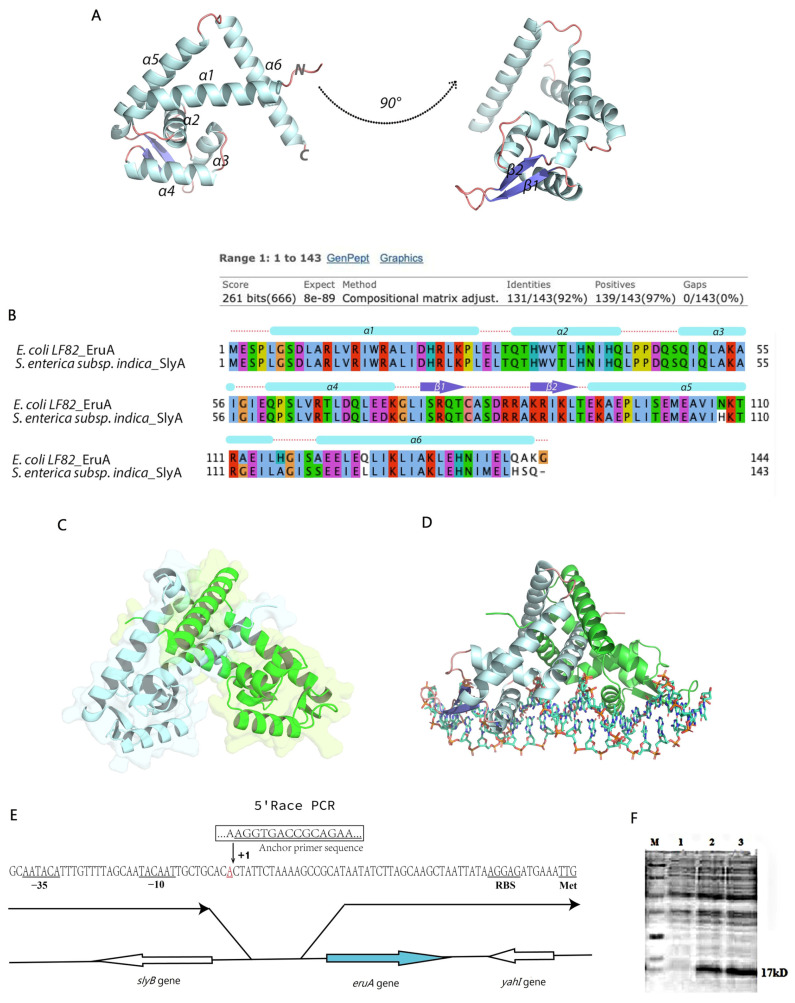
The EruA found in AIEC LF82 was similar to the SlyA in *S. enterica* subsp. *indica*. (**A**) A schematic diagram of an EruA monomer showing secondary structures. α-helices, pale cyan; β-sheet, slate; loop, salmon. (**B**) The EruA amino acid sequence of the AIEC LF82 strain compared with the SlyA amino acid sequence of *S. enterica* subsp. Marked with six rectangles (α-helices), two arrows (β-sheet), and several dashed lines (loop). (**C**) A schematic diagram of an EruA dimer showing secondary structures. (**D**) A schematic diagram of an EruA-DNA model predicated based on the structure of the SlyA-DNA complex as a reference. (**E**) The genetic organization of the *eruA* chromosomal region dissected in the AIEC LF82 strain. Large arrows represented the *eruA* and its adjacent *slyB* and *yahI* genes, and their orientation indicates the transcriptional direction. The nucleotide sequence of the *eruA* promoter region is shown. The transcription initiation nucleotide (+1) identified by 5′RACE is marked with an arrow, and a putative ribosome binding site (RBS) sequence, −10 and −35 sites, and transcription initiation codon (TTG) are underlined. (**F**) The crude extracts from *E. coli* pET28a-eruA/BL21 and control *E. coli* pET28a^+^/BL21 were analyzed with 12% SDS-PAGE following 1 mM IPTG induction for 4 or 6 h at 30 °C. M: protein marker; lane 1: the crude extracts from control *E. coli* pET28a^+^/BL21 induced for 6 h; lane 2–3: the crude extracts from pET28a-eruA/BL21 induced for 4 or 6 h. Original images can be found at Supplementary Materials.

The secondary protein structure of EruA (UFP37798.1) was predicted using Alphafold3 software (Figure 1A). Its ipTM value was 0.81 (>0.8). By BLASTP and Jalview analysis, we observed that the amino acid sequence of EruA of the AIEC LF82 strain showed 92% identity and 97% similarity with that of SlyA of the *S. enterica* subsp. *indica* strain (Figure 1B). Secondary structure elements were annotated and visualized in PyMOL to support comparative structural analysis. In the EruA protein, the helices α1, α5, and α6 formed a dimerization domain, and the helices α3 and α4 formed a canonical HTH of DNA-binding domain. A wing-like region consisted of two antiparallel β-sheets and their connecting loop between helices α4 and α5 (Figure 1A). Based on alignments between EruA and SlyA proteins, the HTH domain was stabilized due to intramolecular hydrophobic interactions between Thr-36, Leu-37, Ile-40, Leu-52, Leu-63, Leu-67, and Leu-70. The helix α2 separates the upper dimerization domain from the lower DNA-binding domain. SlyA forms a dimer, which is an active form in vitro [22]. In the predicted structure, the EruA dimer adopted a triangular arrangement (Figure 1C). Analysis of the EruA dimer interface revealed that Leu-12 from one monomer interacted hydrophobically with Ile-130 and Leu-133 from another monomer in the dimerization domain. Two Leu-126 residues, one from each monomer, interacted with each other to form a hydrophobic dimer interface. Glu-59 and Arg-65 in each monomer formed an intermolecular salt bridge to anchor these helices. Similar to the SlyA-DNA model, the EruA dimer in the EruA-DNA model was located above the DNA duplex, with the DNA helical axis located beneath the symmetry axis of the dimer (Figure 1D).

The size of the ORF appears to be 435 bp, with an initiation codon TTG and a termination codon TGA, respectively. The initiation codon separated by six nucleotides is a putative ribosome binding site sequence (RBS), AGGAG. The *eruA* gene transcript was found using 5′RACE analysis (Figure 1E). We concluded that the A at the upstream of the TTG codon corresponded to the nucleotide +1 of the transcript. A putative 10 box (TAACAT) located eight nucleotides upstream from the transcriptional start point, and a putative sequence near the 35 box separated by 13 nucleotides was consensus ACATAA.

Next, we expressed a recombinant His_6_-tagged EruA protein, encoded by the *eruA* gene. A 17-kDa protein was obtained from the crude extract from *E. coli* pET28a-eruA/BL21 induced by IPTG, while no such protein was present in the crude extract from control *E. coli* pET28a^+^/BL21 (Figure 1F). The molecular weight of the protein based on the *eruA* gene was predicted to be 16.058 kDa, which, coupled with the size of His_6_ tags (0.8 kDa) from the pET28a-eruA plasmid, allowed a protein of about 17 kDa to be produced. The expression abundance of the EruA protein was higher for 6 h than for 4 h of induction. The protein may be a regulator like SlyA in the SlyA family.

### 3.2. The eruA Gene Encodes a Regulator in the AIEC LF82 Strain

**Figure 2:**
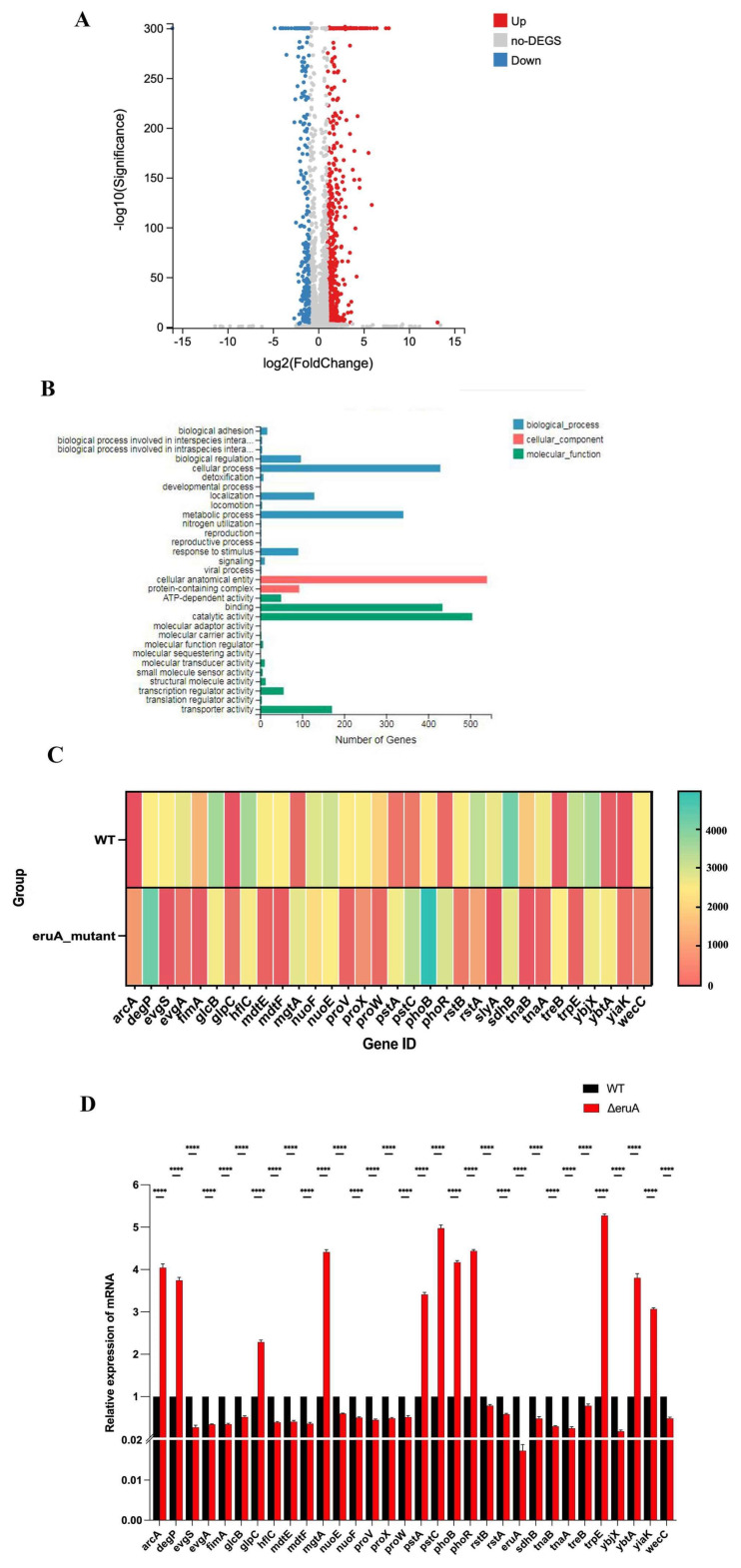
The *eruA* gene encodes a regulator in the AIEC LF82 strain. (**A**) The results of RNA sequencing presented as a volcano plot, showing the number of differentially expressed genes (DEGs) in the ΔeruA bacteria, compared to the WT strain (|log2 fold change| ≥ 2, FDR ≤ 0.001). In the ΔeruA bacteria, the red indicates the upregulated DEGs, and the blue indicates the downregulated DEGs. (**B**) The GO functional categories of these DEGs for ΔeruA and its WT bacteria, including biological process, cellular component, and molecular function. (**C**) The normalized reads for thirty-two DEGs from RNA-sequencing are shown. (**D**) The thirty-two DEGs verified by qRT-PCR. **** p < 0.0001.

As EruA may be a member of the SlyA family that can regulate some target genes in response to environmental stress [16,17], we simulated an oxidative or acidic environment to culture the LF82 strain in vitro, and compared the expression levels of the *eruA* gene in these conditions. The results showed that the highest expression level of the *eruA* was observed when the logarithmic phase LF82 strain was cultivated in LB broth (pH 6.3) for 2 h at 37 °C (Supplementary Materials; Figure S1). In this condition, the transcriptome of the WT bacteria was compared with that of the ΔeruA bacteria. Based on RNA sequencing, the WT bacterial group and the ΔeruA bacterial group, respectively, obtained a total of 96.15% and 95.33% of the read data, and all of these data were mapped onto the reference genome of the AIEC LF82 strain. Next, gene expression profiles of the two samples were compared using the RPKM algorithm. As shown in the volcano plot (Figure 2A), the results displayed that the expressions of DEGs were upregulated or downregulated in the ΔeruA bacteria, compared to the WT bacteria (Table S1).

Gene ontology (GO) was used to classify the functional annotations of these DEGs. A GO category analysis revealed that these DEGs were classified into three functional categories, including biological process, cellular component, and molecular function (Figure 2B). These DEGs were mainly involved in biological adhesion, biological regulation, cellular process, catalytic activity, response to stimulus, localization, metabolism, transport, binding, and transcription regulator activity, and so on. Among these DEGs, normalized reads for thirty-two genes that exhibited consistent expression changes were visualized in a heatmap (Figure 2C), and their differential expression was verified by qRT-PCR (Figure 2D). Functional annotation of these genes revealed representation of multiple biological categories, including regulators (SlyA (EruA), ArcA, PhoB, YbtA), two-component systems (EvgS/EvgA, PhoR, RstAB), transportors (MdtE/MdtF, ProV/ProW/ProX, MgtA, PstA/PstC, TnaB, TreB) or enzymes (DegP, GlcC/GlcB, NuoE/NuoF, SdhB, TnaA, TrpE, WecC, YiaK, HflC), and others (YbjX, FimA). For example, the expression level of the *slyA* (*eruA*) gene in ΔeruA was only 1/16th of that in the WT bacteria (Table S1). SlyA, an analogue of EruA, in *Salmonella* regulates some target genes and responds to the stimulation of hydrogen peroxide and sodium hypochlorite [31]. In *E. coli*, *evgS*/*evgA* encodes a two-component system, which enables the bacteria to adapt to acidic environments [32]. MdtEF-TolC is a proton-driven drug pump in *E. coli* and other bacteria in the intestines. Acid-modulated MdtEF-TolC allows these bacteria to survive in an extreme-acid environment [33]. The proteins encoded by these thirty-two DEGs may be related to the bacteria’s ability to resist oxidative or acidic stress, and so on, and their functions will be suggested in the discussion. These results indicate that deletion of *eruA* has a broad impact on the transcriptional landscape of AIEC LF82. Therefore, we think that the *eruA* gene in AIEC may be a regulator. It might regulate some target genes, enabling the pathogen to survive when facing environmental stresses.

**Figure 3:**
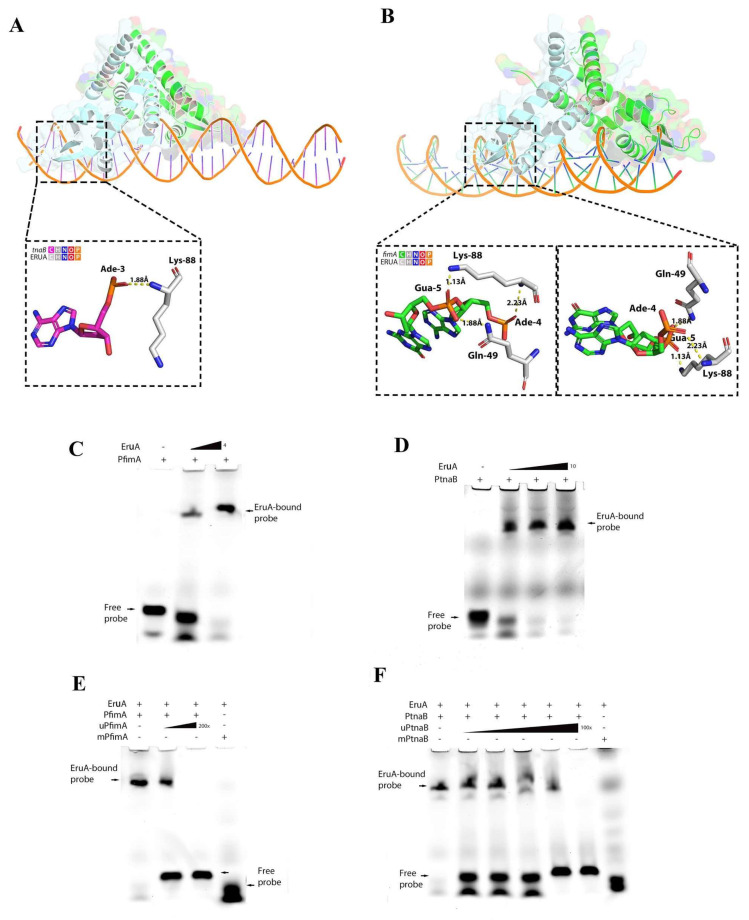
EruA directly regulates the *tnaB* and *fimA* of its target genes in the AIEC LF82 strain. (**A**) A main interaction site between Lys-88 in EruA and TTAAAGGGTTAA of the consensus sequences in the P_tnaB_ identified using YASARA software analysis. Gray and pink, carbon (EruA and DNA, respectively); dark grey, hydrogen; blue, nitrogen; red, oxygen; orange, phosphorus. (**B**) The main interaction sites between Lys-88 and Gln-49 in EruA and TTAAGAGGATAA of the consensus sequences in the P_fimA_ identified using YASARA software analysis. Gray and green, carbon (EruA and DNA, respectively); dark gray, hydrogen; blue, nitrogen; red, oxygen; orange, phosphorus. Nucleic acid and protein residues are depicted by bars, and hydrogen bonds are shown as dashed lines. (**C**) Different concentrations of purified EruA subjected to EMSA with 5′-Cy3-labeled probe PfimA. For the 5′-CY3-PfimA probe (10 nM), EruA concentrations were 0, 2, or 4 μM. (**D**) Different concentrations of purified EruA subjected to EMSA with 5′-Cy3-labeled probe PtnaB. For the 5′-CY3-labeled PtnaB probe (10 nM), EruA concentrations were 0, 6, 8, or 10 μM. (**E**) The specific binding of EruA to the consensus sequences in the promoter region of the *fimA* gene (5′-CY3-labeled PfimA) verified by EMSA. The concentration of 5′-CY3-labeled PfimA was 10 nM, the concentrations of unlabeled probes (uPfimA) were 100 and 200 times those of PfimA, and the concentration of 5′-CY3-labeled mutant probes (mPfimA) was 10 nM. The purified EruA protein was 4 μM. (**F**) The specific binding of EruA to the consensus sequences in the promoter region of the *tnaB* gene (5′-CY3-labeled PtnaB) verified by EMSA. The concentration of 5′-CY3-labeled PtnaB was 10 nM, the concentrations of unlabeled probes (uPtnaB) were 10, 20, 40, 80, and 100 times those of PtnaB, and the concentration of 5′-CY3-labeled mutant probes (mPtnaB) was 10 nM. The purified EruA protein was 10 μM. Original images can be found at Supplementary Materials.

Based on this broad regulatory signature and the presence of conserved promoter motifs in several candidate targets, we focused on *tnaB* and *fimA* genes for targeted validation. In the *S. typhimurium* LT2 strain, the binding site for SlyA is TTAN6TAA within the promoter region of its target genes and contains a near-perfect inverted repeat [22]. We found that the promoter regions of the *fimA* and *tnaB* genes from these DEGs possessed the consensus sequence TTAN6TAA (P_fimA_ and P_tnaB_). Alphafold3 predicted the interaction of the EruA dimer with the consensus pseudo-palindromic DNA sequences of P_fimA_ and P_tnaB_, supporting the hypothesis that EruA may directly engage these promoters to modulate transcription. The ipTM scores were 0.80 and 0.87. In the EruA-DNA complex, the DNA at the P_fimA_ or P_tnaB_ bent toward the bottom of the EruA dimer. The potential intermolecular interactions were further evaluated using YASARA software. The results reveal that hydrogen bonds were the predominant interaction force mediating the binding between the EruA dimer and the P_fimA_ or P_tnaB._ A hydrogen bond between the EruA dimer and TTAAAGGGTTAA in the P_tnaB_ is shown (Figure 3A). The N atom of Lys-88 in EruA formed a hydrogen bond with the O1 atom of the adenine 3 in the consensus sequences of the P_tnaB_ with a hydrogen–oxygen distance of 1.88 Å and a bond energy of 10.55 kJ/mol. Some hydrogen bonds between EruA and TTAAGAGGATAA in the P_fimA_ were observed (Figure 3B). The N_2_ atom of residue Gln-49 in EruA donated a hydrogen bond to the O_2_ atom of guanine 5 in the consensus sequences of the P_fimA_. The hydrogen bond distance was 1.88 Å with a calculated bond energy of 23.85 kJ/mol. The N atoms in the backbone and side chain of Lys-88 in EruA also had two hydrogen bonds with the O1 atoms of adenine 4 and guanine 5 in the sequences of the P_fimA_. The two hydrogen bond distances were 2.23 and 1.13 Å, with calculated bond energies of 18.50 and 17.20 kJ/mol, respectively.

Next, to further determine whether the purified EruA specifically bound to the consensus sequences in the promoter region of the *fimA* and *tnaB,* we performed *EMSA*. The lowest concentrations of EruA that fully bound to the 5′-CY3-labeled PfimA and PtnaB probes were determined, indicating that EruA had a higher affinity for PfimA than for PtnaB (Figure 3C,D). The EMSA results showed that EruA can bind to the CY3-labeled probes PfimA and PtnaB, but could not bind to the mutant probes mPfimA and mPtnaB. These bindings were competitively inhibited by high concentrations of the unlabeled probes uPfimA and uPtnaB (Figure 3E,F). The results show that EruA specifically bound to the TTAAGAGGATAA motif in the promoter region of the *fimA* gene and the TTAAAGGGTTAA motif in the promoter region of the *tnaB* gene. Compared with PtnaB, EruA required a higher concentration of unlabeled uPfimA probe to competitively inhibit the binding of labeled PfimA. This indicates that PfimA has a higher affinity for EruA, which is consistent with the results predicted by AlphaFold3. Therefore, the gene was identified as encoding a regulator, and was therefore designated as the gene *E. coli* LF82 strain regulating gene (*eruA*) (GenBank accession number: MZ475355).

### 3.3. EruA Activates fimA to Promote Bacterial Adhesion to Intestinal Epithelial Cells and tnaB to Increase Production of Indole and Formation of Biofilm

**Figure 4:**
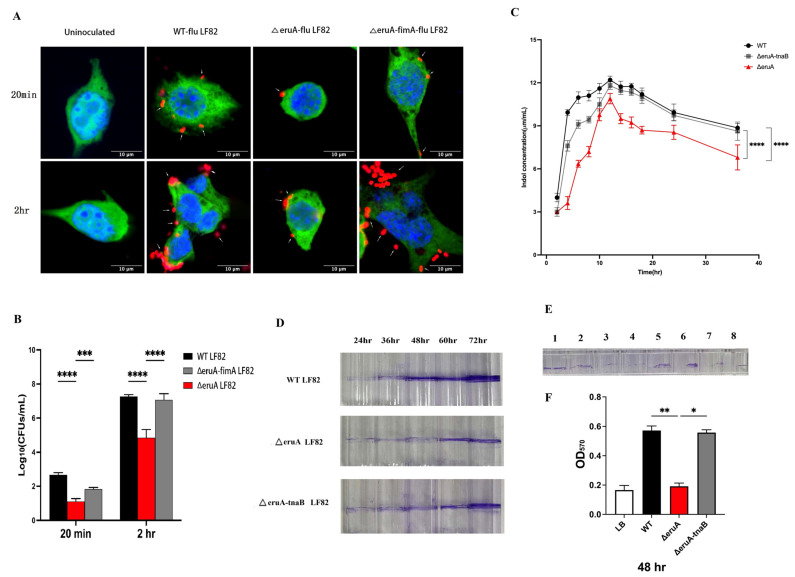
EruA activates the *fimA* gene to promote adhesion to intestinal epithelial cells and the *tnaB* gene to increase the production of indole and the formation of biofilm. (**A**) The number of WT-flu, ΔeruA-flu, and ΔeruA-fimA-flu bacteria adhering to MODE-K cells for 20 min or 2 h of incubation, as observed under an SCM (scale bars, 10 µm; red indicates bacteria; blue indicates cell nuclei, and green indicates cytoplasm within MODE-K cells). The white arrows indicate the AIEC bacteria. (**B**) The number of the ΔeruA bacteria adhering to MODE-K cells compared with that of the WT and ∆eruA-fimA bacteria for 20 min or 2 h of incubation, as determined by counting CFUs/mL on LB plates without or with 35 μg/mL chloramphenicol. (**C**) The concentrations of indole in the supernatants of the ∆eruA bacteria compared with those of the WT and ∆eruA-tnaB bacteria at different culture times using the p-DMAB method. (**D**) Biofilm formation of the ∆eruA bacteria compared with that of the WT and ∆eruA-tnaB bacteria at 24, 36, 48, 60, and 72 h after incubation in 0.2% crystal violet-stained glass tubes. (**E**) Biofilm formation of the ∆eruA bacteria compared with the WT and ∆eruA-tnaB bacteria by incubation in 0.2% crystal violet-stained 96-well plates for 48 h using uninoculated LB medium as a negative control; 1–8 represent biofilm of the WT, WT, ∆eruA, ∆eruA, ∆eruA-tnaB, and ∆eruA-tnaB bacteria, as well as LB and LB broth, respectively. (**F**) The absorbance (570 nm) of the biofilms of the ∆eruA bacteria compared with that of the WT and ∆eruA-tnaB bacteria in the 96-well plates for 48 h using an automatic microplate reader. Data are from three independent experiments and are represented as the mean ± SD. Statistical significance was assessed using one-way ANOVA, followed by Tukey’s test. Note: * *p* < 0.05, ** *p* < 0.01, *** *p* < 0.001; **** *p* < 0.0001. Original images can be found at Supplementary Materials.

EruA directly regulates the *fimA* and *tnaB* of its target genes. FimA is a main subunit of type I fimbriae, which are related to bacterial adhesion [8]. We attempted to find whether EruA affects the adherence of AIEC to epithelial cells. After the MODE-K cells were infected by the AIEC LF 82 strain for 20 min or 2 h, the number of ∆eruA-flu bacteria adhering to MODE-K cells was less than that of WT-flu and ∆eruA-fimA-flu bacteria under an SCM (Figure 4A), and the number of ∆eruA bacteria was significantly lower than that of WT and ∆eruA-fimA bacteria determined by counting CFUs/mL on plates (*p* < 0.001; all three, *p* < 0.0001; Figure 4B). These results show that the *eruA* gene could activate the *fimA* gene to promote the adherence of the LF82 strain to epithelial cells.

TnaA (Tryptophanase) can catalyze tryptophan to generate indole, and tryptophan-specific transporter TnaB is used for transporting tryptophan [14]. Indole is a bacterial signal molecule that can participate in regulating processes such as biofilm formation [15]. We attempted to investigate whether the *eruA* deletion causes the LF82 strain to produce indole. The concentrations of indole in the supernatants of the WT, ∆eruA-tnaB, and ∆eruA bacteria at different culture time points were compared. The results showed that the concentrations increased rapidly in the first 2–12 h, and then decreased slowly for 14–36 h. At 36 h, the indole concentration of the ∆eruA bacteria was significantly different from that of the WT and ∆ eruA-tnaB bacteria (both, *p* < 0.0001; Figure 4C). These results show that the *eruA* gene can activate the *tnaB* gene to allow the LF82 strain to produce indole.

As indole is related to the formation of bacterial biofilm, we further explored whether deletion of *eruA* affects the biofilm. The biofilms formed by the LF82 strain at different times were deposited on glass tubes stained with crystal violet (Figure 4D). The results demonstrate that the biofilms began to form at 24 h and reached a mature state at 48 h. The biofilm of the ∆eruA bacteria was less than that of the WT and ∆eruA-tnaB bacteria. In a 96-well plate stained with crystal violet, the uninoculated LB medium was used as a negative control. After 48 h of cultivation, the biofilm formed by the ∆eruA bacteria was less than that of the WT bacteria and ∆eruA-tnaB bacteria (Figure 4E). The absorbance OD_571_ of the ∆eruA bacteria was significantly lower than that of the WT and ∆eruA-tnaB bacteria (*p* = 0.005, *p* = 0.013; Figure 4F). Therefore, these results show that the *eruA* gene can activate *tnaB* to promote the formation of bacterial biofilm.

### 3.4. EruA Helps AIEC Respond to Acidic and Oxidative Signals and Tolerate Environmental Stresses

**Figure 5:**
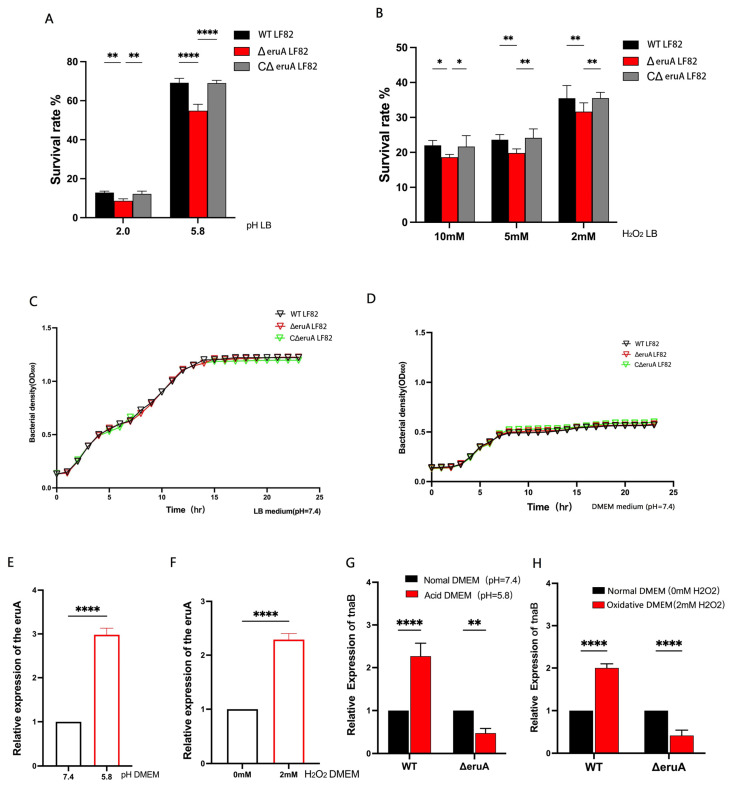
EruA can help the AIEC LF82 strain resist environmental stress. (**A**) The survival rates of the ΔeruA bacteria were significantly lower than those of the WT and CΔeruA bacteria in LB broth (pH 2.0 or 5.8), as determined by counting CFUs on plates. (**B**) The survival rates of the ΔeruA bacteria were significantly lower than those of the WT or CΔeruA strain grown on 10, 5, or 2 mM H_2_O_2_ LB broth, as determined by counting CFUs on plates. (**C**) The growth curve of the ΔeruA bacteria compared with that of the WT and CΔeruA bacteria grown on standard LB (pH 7.4 and 0 mM H_2_O_2_). (**D**) The growth curve of the WT bacteria compared with that of the ΔeruA and CΔeruA bacteria grown in DMEM (pH 7.4 and 0 mM H_2_O_2_). (**E**) The expression levels of the *eruA* gene in the WT LF82 strain in acidified DMEM (pH 5.8) compared with those in standard DMEM (pH 7.4) based on qRT-PCR analysis. (**F**) The expression levels of the *eruA* gene of the WT LF82 strain in oxidative DMEM (2 mM H_2_O_2_) compared with those in standard DMEM (0 mM H_2_O_2_) according to qRT-PCR analysis. (**G**) The expression levels of the *tnaB* gene in the WT or ΔeruA bacteria cultured in acidified DMEM relative to standard DMEM, as determined using qRT-PCR analysis. (**H**) The expression levels of the *tnaB* gene in the WT or ΔeruA bacteria cultured in oxidative DMEM (2 mM H_2_O_2_) relative to standard DMEM (0 mM H_2_O_2_), as determined using qRT-PCR analysis. Data are from three independent experiments and are represented as the mean ± SD. Statistical significance was assessed using one-way ANOVA, followed by Tukey’s test. Note: * *p* < 0.05, ** *p* < 0.01, **** *p* < 0.0001.

Since some target genes from DEGs may be involved in the bacterial response to acidic or oxidative stress, we compare the survival rates of the ΔeruA and WT bacteria in a simulated gastric acidic environment, as well as acidic or oxidative environments of phagolysosomes within macrophages. In the acidified LB (pH 2), the rate of the ΔeruA bacteria was significantly lower than that of the WT and CΔeruA bacteria (*p* = 0.0012, *p* = 0.0064). In the less-acidified LB (pH 5.8), the rate of the ΔeruA bacteria was significantly lower than that of the WT and CΔeruA bacteria (both, *p* < 0.0001; Figure 5A). All survival rates of the ΔeruA bacteria were also significantly lower than those of the WT (*p* = 0.0212, *p* = 0.0095, and *p* = 0.0089) and CΔeruA (*p* = 0.0396, *p* = 0.0030, and *p* = 0.0083) bacteria in LB containing 10, 5, or 2 mM H_2_O_2_ (Figure 5B). However, in standard LB broth (pH 7.4; Figure 5C) and DMEM (pH 7.4; Figure 5D), the growth curve of the ∆eruA bacteria was not significantly different from that of the WT and C∆eruA bacteria. Therefore, EruA helps the LF82 strain adapt to acidic and oxidative environments.

Moreover, when the WT bacteria were cultured in acidified DMEM (pH 5.8), the expression level of the *eruA* gene was significantly different compared with cultures in standard DMEM (pH 7.4) (*p* < 0.0001; Figure 5E). There was also a significant difference in the expression level of the *eruA* gene between the LF82 strain cultured in the oxidative DMEM (containing 2 mM H_2_O_2_) and the strain cultured in the standard DMEM (without H_2_O_2_) (*p* < 0.0001; Figure 5F). Overall, these results suggest that the expression level of the *eruA* gene in the WT bacteria was upregulated under acidic or oxidative stress.

As indole functions as a signaling molecule involved in bacterial resistance [15], we investigated the *tnaB* expression in response to acidic or oxidative stress by measuring the *tnaB* mRNA levels in the WT and ΔeruA bacteria cultured in acidic or oxidative DMEM. The results indicated that compared with standard DMEM, acidic or oxidative treatment increased the expression of the *tnaB* in the WT bacteria (both, *p* < 0.0001). However, the ΔeruA bacteria abolished the upregulation of the *tnaB* induced by acidic or oxidative stress (*p* < 0.0082, *p* < 0.0001; Figure 5G,H). These results show that EruA could activate the *tnaB* gene and help the LF82 strain resist environmental stress.

### 3.5. EruA Promotes the Survival of AIEC Within Macrophages and Bacterial Virulence

**Figure 6:**
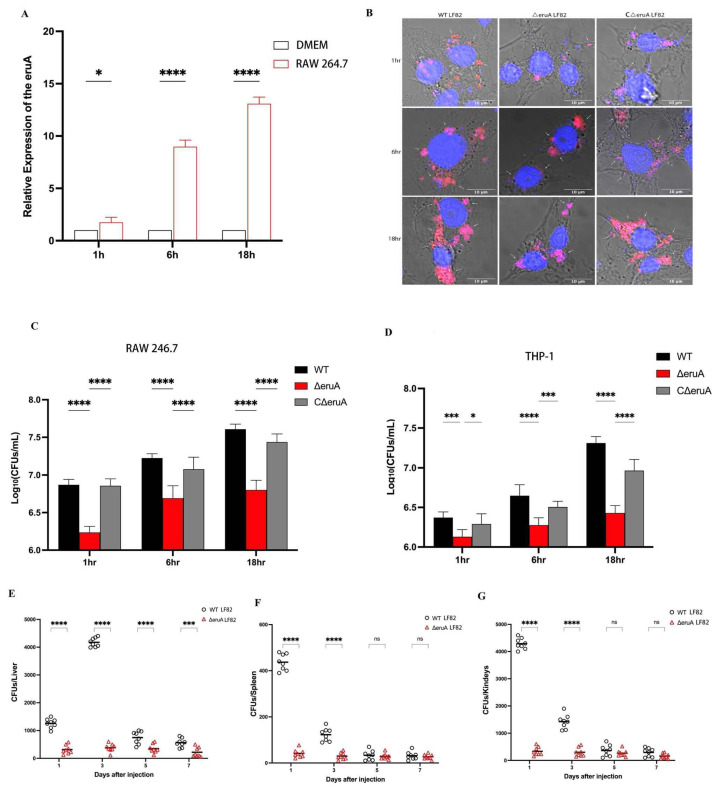
EruA promotes the bacterial survival of the AIEC LF82 strain within macrophages and loading in mouse tissues. (**A**) The expression levels of the *eruA* gene of the LF82 strain grown in RAW 264.7 macrophages for 1, 6, and 18 h compared with those of the LF82 strain cultured in standard DMEM using qRT-PCR. (**B**) The numbers of WT-flu, ΔeruA-flu, and CΔeruA-flu bacteria cultured in RAW 264.7 macrophages for 1, 6, and 18 h of incubation observed under an SCM (scale bars, 10 µm; bacteria, red; cell nuclei, blue). The white arrows indicate the AIEC bacteria. (**C**) The number of ΔeruA bacteria cultured in RAW 264.7 macrophages compared with those of the WT or CΔeruA bacteria for 1, 6, and 18 h of incubation, as determined by counting CFUs/mL on LB plates. (**D**) The number of ΔeruA bacteria within THP-1 macrophages compared with those of the WT or CΔeruA bacteria for 1, 6, and 18 h of incubation, as determined by counting CFUs/mL on LB plates. (**E**–**G**) Each mouse was intraperitoneally injected with the WT or ΔeruA bacteria. The bacterial loads of the WT or ΔeruA bacteria in the livers (**E**), spleens (**F**), and kidneys (**G**) were determined on days 1, 3, 5, and 7 post-infection by counting CFUs/mL on LB plates. Data are from three independent experiments and are represented as the mean ± SD. Statistical significance was assessed using one-way ANOVA, followed by Tukey’s test. Note: * *p* < 0.05, *** *p* < 0.001, **** *p* < 0.0001; ns, not significant.

As the *eruA* gene is involved in the bacterial response to stress signals and in tolerating environmental stresses, its protein may regulate the intracellular survival of AIEC within macrophages. First, we investigated the expression levels of the *eruA* gene in the LF82 strain when infected with RAW 264.7 macrophages (Figure 6A). The results showed that its expression levels gradually increased at 1, 6, and 18 h after the LF82 strain had infected the cells, and when only using standard DMEM for cultivation of the bacteria, those expression levels remained stable. The expression levels of *eruA* when the LF82 strain infected the cells were higher than those when only using standard DMEM for cultivation of the bacteria (*p* = 0.0118, both *p* < 0.0001). The results suggested that the upregulation of the *eruA* may contribute to AIEC adapting to the environment within macrophages. Next, we further investigated whether EruA affected the replication of AIEC within macrophages. It was observed under an SCM that intracellular numbers of the ΔeruA bacteria decreased in RAW 264.7 macrophages compared with those of the WT or CΔeruA bacteria (Figure 6B). The number of intracellular bacteria increased with the incubation time, as determined by counting CFUs/mL on LB plates (Figure 6C). The intracellular number of ΔeruA was significantly lower than that of the WT and CΔeruA bacteria after 1, 6, and 18 h of culture in RAW 264.7 macrophages (all *p* < 0.0001). The results of AIEC replication performed in THP-1 macrophages showed the same trend as RAW 264.7 macrophages at 1, 6, and 18 h (*p* < 0.001, *p* = 0.0204; all three, *p* < 0.0001; *p* < 0.001; Figure 6D). Overall, the results indicate that EruA affects the replication of AIEC within macrophages.

As bacterial replication within macrophages favors bacterial spread in animals, we compared the WT and ΔeruA bacterial loads in various organs in an i.p. infection mouse model. The results showed that compared with the WT bacteria, the ΔeruA bacteria had reduced dissemination to the livers (all three, *p* < 0.0001; *p* < 0.001; Figure 6E), spleens (both, *p* < 0.0001; *p* = 0.9660; *p* = 0.8733; Figure 6F), and kidneys (both, *p* < 0.0001; *p* = 0.6589; *p* = 0.3739; Figure 6G) on days 1, 3, 5, and 7 post-infection. In conclusion, these data suggest that EruA favors the virulence of AIEC.

### 3.6. EruA Contributes to AIEC Colonization and Exacerbates Intestinal Inflammation in Mice with DSS-Induced Colitis and Those That Are DSS-Free

**Figure 7:**
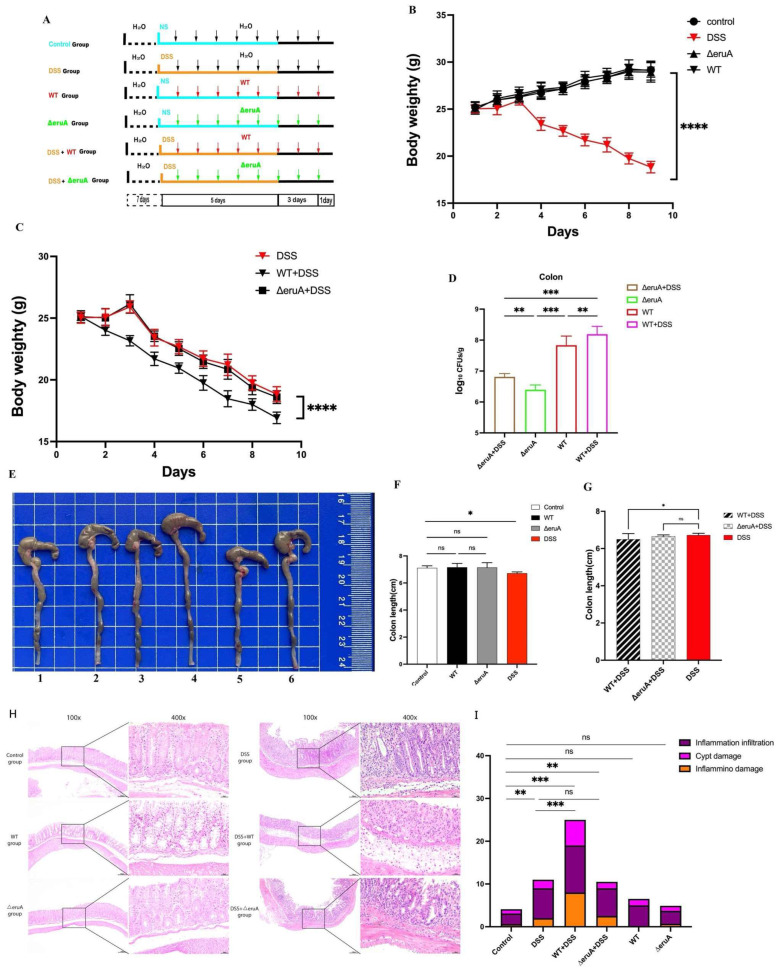
EruA contributes to AIEC colonization and exacerbates intestinal inflammation in mice with DSS-induced colitis and those that were DSS-free. (**A**) Mice in the Control, WT, ∆eruA, DSS, DSS + ∆eruA, and DSS + WT groups were orally administered 2.5% DSS or NS, and/or the WT or ∆eruA bacteria for bacterial infection. (**B**) The body weights in the Control, DSS, WT, and ∆eruA groups, as recorded from the first day after NS or DSS was added to the drinking water. (**C**) The body weights in the DSS, DSS + WT, and DSS + ∆eruA groups, as recorded from the first day after DSS was added to the drinking water. (**D**) The number of WT and ∆eruA bacteria colonized in the colons of mice with and without DSS-induced colitis. (**E**) All mouse colons in the six groups were harvested on day 17. Numbers 1–6 represent the colons in the DSS, WT, ∆eruA, Control, DSS + ∆eruA, and DSS + WT groups, respectively. (**F**) The colon lengths in the DSS, WT, and ∆eruA groups compared with those in the Control group. (**G**) The colon lengths in the DSS + ∆eruA and DSS + WT groups compared with those in the DSS group. (**H**) Representative pictures of H&E-stained colon tissues in the Control, WT, ∆eruA, DSS, DSS + ∆eruA, and DSS + WT groups (scale bars, 100 µm; magnification, 100×, 400× (objective × eyepiece)). (**I**) The cumulative histology scores of the colon tissues in the control group compared with those in the WT, ∆eruA, DSS, DSS + ∆eruA, and DSS + WT groups, and the cumulative histology scores of the colon tissues in the DSS group compared with those in the DSS + ∆eruA and DSS + WT groups. Data are represented as the mean ± SD, with n = 6–8 per group. Statistical significance was assessed using one-way ANOVA, followed by Tukey’s test. Note: * *p* < 0.05, ** *p* < 0.01, *** *p* < 0.001, **** *p* < 0.0001; ns, not significant.

AIEC can replicate within macrophages in the mucous lamina propria of CD patients, resulting in the pathogen crossing the intestinal barrier and invading deeper tissues [4]. We investigated whether EruA contributes to AIEC colonizing in mice, both in mice with DSS-induced colitis and those that were DSS-free. Mice were orally administered 2.5% DSS or NS, with or without the WT or ΔeruA bacteria, on consecutive days (Figure 7A). From the third day of DSS induction, the body weights in the Control, WT, and ΔeruA groups increased (Figure 7B), whereas those in the DSS, DSS + ΔeruA, and DSS + WT groups decreased (Figure 7C). On day nine post-infection, the body weights in the DSS group were significantly lower than those in the Control, WT, and ΔeruA groups (all, *p* < 0.0001), and there were no significant differences in the body weights among the Control, WT, and ΔeruA groups (*p* = 0.9983, *p* = 0.9348, *p* = 0.9747; Figure 7B). The body weights in the DSS + WT group were significantly lower than those in the DSS or DSS + ΔeruA group (both, *p* < 0.0001), and there was no significant difference in the body weights between the DSS and DSS + ΔeruA groups (*p* = 0.7645; Figure 7C). These results showed that the mice model with DSS-induced colitis had been successfully established.

In DSS-free mice, the number of ∆eruA bacteria colonized in the colons of the ∆eruA group significantly decreased, compared with that of the WT bacteria in the colons of the WT group (*p* < 0.001; Figure 7D). In mice with DSS-induced colitis, the number of ∆eruA bacteria colonized in the colons of the DSS + ∆eruA group was significantly lower than that of the WT bacteria in the colons of the DSS + WT group (*p* < 0.001). Moreover, the number of WT and ∆eruA bacteria colonized in the colons of DSS-free mice was significantly lower than that of the WT and ∆eruA bacteria in the colons of DSS-induced mice, respectively (*p* < 0.010; *p* < 0.003). Therefore, the results suggested that EruA helped the LF82 strain colonize the colon in both DSS-free induced and DSS-induced mice, with a stronger effect observed in DSS-induced mice. Together, these results show that EruA enhances the pathogenicity of AIEC in mice regardless of DSS treatment.

Next, we further investigated whether EruA helped the LF82 strain exacerbate intestinal inflammation in mice. The colon length was used to evaluate inflammation severity in the mouse model with DSS-induced colitis. Compared with the Control group, the colon lengths in the mice of the DSS group were significantly shorter (*p* = 0.0253), while there were no differences in colon lengths among the Control, WT, and ∆eruA groups (*p* = 0.9831, *p* = 0.9952, *p* = 0.9992; Figure 7E,F). However, compared with the DSS group, the colon lengths in the DSS + WT group were significantly shorter (*p* = 0.0485), while there was no significant change in the colon lengths in the DSS + ∆eruA group (*p* = 0.7096; Figure 7E,G). The H&E-stained colon tissues in the Control, WT, and ∆eruA groups seemed to be normal, while the epithelial barriers were eroded and ulcerated, the crypts were damaged, and inflammatory cells were infiltrated in the DSS, DSS + ∆eruA, and DSS + WT groups (Figure 7H). There was no significant difference in the degree of colonic inflammation between the WT or ∆eruA group and the Control group (*p* = 0.670, *p* = 0.382). Compared with the Control group, the degree of colonic inflammation in the DSS group significantly increased (*p* = 0.002; Figure 7I). The degree of colonic inflammation in the DSS + WT group was significantly higher than that in the DSS group (*p* < 0.001), while there was no significant difference between the DSS + ∆eruA group and the DSS group (*p* = 0.895). The cumulative scores for each group were compared, and the results (Figure 7I) were consistent with those of Figure 7H. On the whole, oral administration of the WT bacteria exacerbated DSS-induced colitis more than the administration of the ∆eruA.

## 4. Discussion

AIEC colonization in the intestines and replication within macrophages contribute to the pathogen’s persistence in patients with CD [7,9]. In the present study, EruA was identified as a regulator of AIEC involved in the pathogenesis of IBD [3]. EMSA confirmed that EruA directly and sequence-specifically binds to *fimA* and *tnaB,* supporting a direct regulatory role. It activated the *fimA* gene, promoted bacterial adhesion to intestinal epithelial cells, and colonized the intestines of both DSS-free and DSS-induced colitis mice. EruA directly activated the *tnaB* gene, which enhanced bacterial production of the signaling molecule indole and the formation of bacterial biofilms. Eha, another member of the SlyA family, activated the *aroC* gene encoding chorismate synthase [34], which is required for the production of indole to assist in the formation of bacterial biofilms in *Edwardsiella tarda* (*E. tarda*) [27]. Interestingly, AIEC can assemble biofilm-like communities to avoid being cleared by lysosomes and replicate within macrophages [11]. EruA contributes to AIEC biofilm formation to promote bacterial replication within macrophages.

In addition, based on RNA sequencing (Figure 2C), there is a *slyA* gene, which is an orthologue of the *eruA* gene. Firstly, why did RNA sequencing automatic annotation mislabel the *eruA* gene as *slyA*? As EruA in AIEC shares high amino acid homology with SlyA in *Salmonella* and other transcriptional regulators in the SlyA family, the RNA sequencing annotation software (based on standard NCBI/RefSeq databases) automatically assigned the gene to “*slyA*”. The naming of the *eruA* occurred after RNA sequencing analysis. Next, the genome of the AIEC LF82 strain (GenBank accession no: NC_011993) contains only a canonical slyA-like *eruA* gene. Moreover, EruA does not regulate the *fimA* and *tnaB* genes by means of an “on/off” system but rather exerts its effect on these two target genes by means of a dose-dependent relationship (Figure 3C,D). The binding specificity was further validated by mutating the TTAN_6_TAA motif in mPfimA and mPtnaB. Loss of binding in the mutated probes confirmed that EruA interacted directly and sequence-specifically with these promoters, supporting a direct regulatory role rather than an indirect effect via global regulators (Figure 3E,F). Furthermore, for AIEC, the bacterial adhesion ability and resistance against acidic or oxidative stress, as well as the formation of bacterial biofilms, are not only regulated by the *eruA* gene but also by other regulatory genes and two-component system genes [32]. This is the reason why the impact of deleting the *eruA* gene is not particularly significant.

In addition, in the non-pathogenic *Escherichia coli* strain K-12, the overexpression of a *slyA*_(EC)_ gene (another gene homologous to *eruA*) led to the emergence of a hemolytic phenotype [35]. However, like *slyA* in *S. typhimurium*, *eruA* helps AIEC respond to acidic and oxidative signals and tolerate environmental stresses [16,17]. The *slyA* in *S. typhimurium* is not directly activated by sensing signals; instead, its concentration increases under environmental stresses through the action of other transcriptional factors [36]. However, how the *eruA* helps AIEC respond to such signals remains unclear.

Among the target genes of EruA, in *E. coli*, both *evgS*/*evgA* encoding a two-component system and *mdtE*/*mdtF* encoding a proton-driven drug pump MdtEF-TolC contribute to the bacterial resistance to acidic environments [32,33]. In addition, the membrane sensor protein PhoR and transcription regulator PhoB both belong to the two-component system. In *S. enterica*, when phosphorus is deficient, PhoR and PhoB are activated and help maintain the bacterial inorganic phosphate homeostasis [37]. These regulations play an important role under phosphate limitation, nitrogen limitation, and acidic conditions [38]. In *Listeria monocytogenes* (*L. monocytogenes*), TreB is the sole trehalose transporter. Trehalose metabolism enhances the formation of bacterial biofilm and acid resistance [39]. In the study, when the WT bacteria were cultured in acidic LB medium (pH 5.8), the expression levels of the *phoB*, *phoR*, *rstA,* and *rstB* genes were significantly different compared with culture in standard LB medium (pH 7.4) (Figure S2A). Overall, these results indicate that under acidic stress conditions, the aforementioned target genes of the *eruA* gene in AIEC bacteria may be able to play a role. Therefore, the *eruA* gene may activate these target genes under acidic conditions, thus resisting acid stress.

Among the target genes of EruA, ArcA, encoded by *arcA*, is also a key regulator that inhibits aerobic respiration in Gram-negative bacteria to adapt to environmental changes [40]. The *pstFEDCBA* gene cluster encodes a high-affinity phosphate transporter. Compared with the WT bacteria of *Lactococcus lactis*, the *pstA* mutant was less responsive to copper and zinc and more sensitive to oxygen [41]. Moreover, NuoF and SdhB, encoded by the *nuoF* and *sdhB* genes, are two important enzymes in the respiratory chain of *Xanthomonas oryzae* pv. *Oryzae* (Xoo). Parthenolide can bind to NuoF and SdhB, impairing the function of these enzymes and leading to ROS accumulation in Xoo [42]. DegP, encoded by the *degP* gene, is a highly conserved periplasmic protease in bacteria. The *degP* mutant in *Streptococcus pyogenes* was more sensitive to high temperature and reactive oxygen intermediates than the WT bacteria [43]. In addition, the expression level of the *trpE* gene, encoding anthranilate synthetase, was increased when *L. monocytogenes* proliferated in macrophages, suggesting that the *trpE* gene was involved in helping the bacteria overcome oxidative stress and contributed to their intracellular survival [44]. GlcB (malate synthase), encoded by the *glcB* gene in *Pseudomonas aeruginosa*, is a component of the glyoxylate shunt (GS) pathway, an alternative to the tricarboxylic acid (TCA) cycle. The *glcB* gene expression is upregulated when the bacteria infect the host and combat oxidative stress [45]. Membrane microdomains, also known as lipid rafts, belong to the SPFH superfamily and regulate membrane-dependent functions. Four genes (*yqiK*, *qmcA*, *hflK,* and *hflC*) encode SPFH proteins in Gram-negative bacteria, which help the bacteria resist aminoglycoside antibiotics and oxidative stress [46]. The *yiaK-S* operon in *E. coli* is required for the aerobic utilization of L-ascorbate and has been implicated in bacterial resistance to oxidative stress [47]. In this study, there was also a significant difference in the expression levels of the *phoB*, *phoR*, *rstA,* and *rstB* genes between the LF82 strain grown in oxidative LB medium (2 mM H_2_O_2_) and that grown in the standard LB medium (0 mM H_2_O_2_) (Figure S2B). Overall, these results indicate that under aerobic stress conditions, the aforementioned target genes of the *eruA* gene in AIEC bacteria may be able to function. Therefore, the *eruA* gene may activate these target genes to help bacteria resist oxidative stress.

In addition, among the other target genes of EruA, the *proU* gene cluster encodes a transport system consisting of the *proV*, *proW,* and *proX* genes. In *Shigella*, this system efficiently eliminates glycine betaine and proline betaine, thereby protecting bacteria from extreme osmotic stress and promoting their survival within the cells and the infection process [48]. The *ybtA* gene encodes a global regulator, which can regulate the expression of a high-pathogenicity island as well as the process of iron ion uptake in *Yersinia* [49]. The *ybjX* gene encodes an outer membrane protein that functions as an efficient adaptive barrier, helping avian pathogenic *E. coli* resist environmental stress and survive in macrophages [50]. Two *wecC* and *wecB* genes respectively encode UDP-N-acetyl-d-mannosamine dehydrogenase and UDP-N -acetylglucosamine 2-epimerase, which are crucial for the survival of *Yersinia pestis* (*Y. pestis*) in murine macrophages [51]. Therefore, EruA may activate these target genes to respond to osmosis and other signals, enabling bacteria to replicate within macrophages.

In summary, thirty-two DEGs of the *eruA* gene were found by RNA sequencing and qRT-PCR. EruA in AIEC may both positively and negatively regulate these genes, which may encode various regulators, two-component systems, transport systems, enzymes, and others that help AIEC replicate within macrophages and resist oxidative, acid, osmosis, and other stresses. Moreover, understanding the role of such regulators in pathogens is of vital importance for effectively controlling bacteria and preventing infections. In *Salmonella*, the transcriptional regulator SlyA activates the PhoP/PhoQ two-component system, thereby controlling the expression of numerous genes within macrophages and influencing the bacterial virulence [52]. The *emrR* encodes a regulator in the SlyA/MarR family. This regulator plays a crucial role in the virulence of *S. Typhimurium* and *Y. pestis* of the *Enterobacteriaceae* family and is activated by the regulator SlyA and PhoP/PhoQ [53]. A type III secretion system (T3SS), a specialized transport system, helps Gram-negative pathogenic bacteria inject multiple effectors into host cells. A gene cluster, a virulence island, encodes T3SS and is regulated by regulators such as SlyA in response to environmental changes [54]. However, the interaction between the regulator EruA and the two-component system remains unclear.

Lastly, AIEC is different from infectious pathogens such as *S. typhi*, as AIEC only causes disease under specific host conditions. In this study, when DSS-free mice were administered the WT or ∆eruA bacteria, no intestinal inflammation was observed in the mice. In DSS-induced colitis mice, the WT bacteria aggravated the epithelial barrier damage and colonic inflammation, whereas the ∆eruA bacteria had no significant change. This might be because the WT bacteria survived longer within macrophages and colonized more in intestinal epithelial cells than ∆eruA bacteria. These findings are consistent with the report by Drouet et al., who found that AIEC cannot colonize normal or NOD2 knockout mice without antibiotic or DSS treatment [55]. Therefore, in the presence of intestinal inflammation, the expression of the *eruA* gene may favor the competitive growth of AIEC in the gut.

In addition, AIEC is different from commensal bacteria such as *E. coli*. Mice were orally administrated 2.5% DSS or NS, and/or AIEC LF82 or the *E. coli* DH5α strain, over consecutive days (Figure S3A). The results suggested that the LF82 strain was more capable of colonizing the colon than the DH5α strain, both in mice with either DSS-free or DSS-induced colitis. Compared with DSS-free mice, the LF82 strain was more favorable for colonization in mice with DSS-induced colitis (Figure S3D). Moreover, the oral administration effect of the LF82 strain was the same as that of the DH5α strain in DSS-free mice (Figure S3B,E,F,H,I). However, oral administration of the LF82 strain more effectively aggravated intestinal colitis than that of the DH5α strain in the DSS-induced colitis mice, as shown by weight loss, colon length shortening, and pathological changes in colon tissues (Figure S3C,E,G–I).

## 5. Conclusions

EruA is a novel member of the SlyA family and serves as a master regulator in AIEC. It activates the *fimA* gene to promote bacterial adhesion to intestinal epithelial cells and the *tnaB* gene to increase indole production and biofilm formation. EruA also contributes to the pathogen’s resistance to environmental stresses and improves the bacterial survival rates within macrophages, thereby increasing virulence. Overall, EruA promotes bacterial colonization in the gut and aggravates intestinal inflammation in mice with DSS-induced colitis, ultimately enhancing bacterial pathogenicity and influencing the management of AIEC.

## Figures and Tables

**Table 1:** Bacteria and plasmids listed in this work.

Strain or Plasmid	Relevant Characteristics	Source
Strains		
SM10λpir	*E. coli*; RP4-2 (Km::Tn7,Tc::Mu-1), pro-82, LAMpir, recA1, endA1, thiE1, hsdR17, creC510	Prof. Liang [24]
BL21(DE3)	*E. coil*; F-, lon-11, Δ(ompT-nfrA)885, Δ(galM-ybhJ) 884	Stratagene
WT LF82	AIEC; wild type; Amp^r^; isolated from human	Prof. Zhu [23]
∆eruA LF82	AIEC; eruA Deletion mutant of LF82; Amp^r^	This study
C∆eruA LF82	AIEC; ∆eruA containing expression plasmid pACYC184-eruA; Cm^r^, Amp^r^	This study
∆eruA-fimA LF82	AIEC; ∆eruA containing expression plasmid pACYC184-fimA; Cm^r^, Amp^r^	This study
∆eruA-tnaB LF82	AIEC; ∆eruA containing expression plasmid pACYC184-tnaB; Cm^r^, Amp^r^	This study
WT LF82-flu	AIEC; containing pBBbr1mcs2-Tac-mcherry plasmid; Kan^r^, Amp^r^	This study
∆eruA LF82-flu	AIEC; ∆eruA containing pBBbr1mcs2-Tac- mcherry plasmid; Kan^r^, Amp^r^	This study
C∆eruA LF82-flu	AIEC; C∆eruA containing pBBbr1mcs2-Tac- mcherry plasmid; Cm^r^; Kan^r^, Amp^r^	This study
plasmids		
pDS132	Suicide plasmid, mob RP4 oriR6K, SacB, Cm^r^	Prof. Liang [24]
pACYC184	origin, p15A vector; Tet^r^, Cm^r^	Stratagene
pET28a^+^	origin, F1 vector, promoter T7, expression plasmid; Kan^r^	Novagen
pET28a-eruA	435 bp DNA fragment encompassing the *eruA* cloned into pET28a^+^; Kan^r^	This study
pDS132-F1F2	DNA fragment encompassing upstream and downstream of eruA cloned in pDS132, Cm^r^	This study
pACYC184-eruA	DNA fragment encompassing eruA ORF cloned in pACYC184; Cm^r^	This study
pACYC184-fimA	DNA fragment encompassing fimA ORF cloned in pACYC184; Cm^r^	
pACYC184-tnaB	DNA fragment encompassing tnaB ORF cloned in pACYC184; Cm^r^	
pBBbr1mcs2-Tac-mcherry	origin, Rep pBBR1, oriV, promoter Tac, red fluorescence plasmid; Kan^r^	Morzanbio

**Table 2:** PCR primers or probes and product sizes listed in this work.

Gene Name	Primer Sequence (5′-3′)	Product Size	Methods
RC1145-RT3	AAGTTTTGCGATGAGCTTAATCAG	-	5′RACE-RT
RC1145-RT4	CGGGTTTTGTTAATAACGGCTT		
RC1145-R3	GATCGCTGGCACAAGTTTGAC	240 bp	5′RACE-PCR (First round)
5′ adaptor	GCTGTCAACGATACGCTACGTAACGGCATGACAGTGGGIIGGGIIGGGIIG		
RC1145-RT4	CGGGTTTTGTTAATAACGGCTT	257 bp	5′RACE-PCR (Second round)
5.3′ outer	GCTGTCAACGATACGCTACGTAAC		
RC1145-MZ-Y-F	TTGCGATGAGCTTAATCAGTTGTT	380 bp	5′RACE-PCR
RC1145-MZ-Y-R	AGGTTCTGATCTGGCACGGTT		
F1-F	CATGCATGCATGATGCCATATACTTTGACCATGAG	515 bp	PCR
F1-R	GCTCTAGAGCCACCTGACTGCCATTGCTG		
F2-F	GCTCTAGAGCAAGATATTATGCGGCTTTTAGAA	582 bp	PCR
F2-R	CGAGCTCGCAATAATACGTCCCGACACCT		
X-F	GGAATTCCCCTTCATTTCACCCTTTG	435 bp	PCR
X-R	CAAGCTTCGCATAATATCTTAGCAAGCTAATT		
eruA-F	CGGAATTCCGCCCTTCATTTCACCCTTTG	435 bp	PCR
eruA-R	CCCAAGCTTGGGCGCATAATATCTTAGCAAGCTAATT		
eruA-F	CGACTGGTCTGGAGGTAA	140 bp	qRT-PCR
eruA-R	AATCGCCACTAGGTTCTG		
fimA-F	CGGAATTCCGACGACGGTAAATGGTGGGAC	422 bp	PCR
fim-R	CCCAAGCTTGGCAGCACCGGTTGCAAAATA		
tnaB-F	CGGAATTCCGAAAGCGTGGTTTTCGGTTCG	229 bp	PCR
tnaB-R	CCCAAGCTTGTTCTTGCGGGCTTTGATTGC		
evgA-F	GCAGAGTTGACTGAAGGCGGA	197 bp	qRT-PCR
evgA-R	GCATCAGCACAATGTTTCCCG		
tnaB-F	GCTTTACCTGTTGACCTTGC	198 bp	qRT-PCR
tnaB-R	GGCAACGGTAATACCGCTGA		
glpC-F	CACCAGCTTCGAAAACTGCATT	167 bp	qRT-PCR
glpC-R	AGTTGATGCAATATTTCAGCGCC		
yiaK-F	AGCAGCCTTTAATCGGGTCT	123 bp	qRT-PCR
yiaK-R	GGGAAACGATTAACGCCGTGAG		
tnaA-F	TTTGACCTTGAGGGATTAG	130 bp	qRT-PCR
tnaA-R	ACATCGCTTTTAAGTTTGC		
arcA-F	ATTCGGTAACGGCTTCATA	109 bp	qRT-PCR
arcA-R	TCGTCATTTAAGTGGTGGG		
fimA-F	GCAATCGGCTACAAACAAC	89 bp	qRT-PCR
fimA-R	TGGTAAATGCCGCTTGTGC		
phoR-F	TTGATGTGCCGATGATGCT	113 bp	qRT-PCR
phoR-R	TTCGTTGCCTGACACCTTG		
degP-F	AACGGCGGCTGAGACTTCTT	127 bp	qRT-PCR
degP-R	CGGCGTATTAACGGTTGTGC		
evgS-F	GCAAGGAACAACGGGTGGAG	183 bp	qRT-PCR
evgS-R	CAGCAATCGCAGCGAAAGAG		
glcB-F	TTTGCCCATCCACAACAACC	203 bp	qRT-PCR
glcB-R	TCATCTGCTGGCGTTAAGTCG		
hflC-F	ATCGTGCTGGTAGTGCTTT	184 bp	qRT-PCR
hflC-R	TGGTCTGAATACGTGCGTC		
mdtE-F	ACACCGCACGCACCCAGTT	135 bp	qRT-PCR
mdtE-R	CCGACGATTTCCCGCTGAC		
mdtF-F	GATAGGGTTACTGGTGGAT	259 bp	qRT-PCR
mdtF-R	GCTCATTGCTACAAATACTG		
mgtA-F	TTTGCCGCAGGTGTTATCG	156 bp	qRT-PCR
mgtA-R	GCCATTTTCGCCTTTGTCG		
nuoF-F	TGCGTGGGCACAGAAAGTT	265 bp	qRT-PCR
nuoF-R	CATCGGTAAAGCGGGAAGC		
nuoE-F	AACCGTTGATGTGACAGAC	185 bp	qRT-PCR
nuoE-R	GAAAATCGTTCAGAAGCAG		
proV-F	GGATGCGTCAACGAGTGGG	145 bp	qRT-PCR
proV-R	CGCTGATGTTTCGCCTGTA		
proX-F	TCAATAAACCCAGCGAAGT	182 bp	qRT-PCR
proX-R	GATCAGGTAACCCTGTGCC		
proW-F	TATTCGTCCACTGCTTGAT	240 bp	qRT-PCR
ProW-R	AGCGGTAACTGAACTTTGA		
pstA-F	AGCAGCAGTACGCACAGGG	273 bp	qRT-PCR
pstA-R	TACGCCGAAGTGGAAGATG		
pstC-F	CCAGATAACTTCCCAGGTG	235 bp	qRT-PCR
stC-R	GTTCGCCGTTTACTTCCAG		
phoB-F	AAGAACCGCTGGAGATGGG	163 bp	qRT-PCR
PhoB-R	TTACGCAGGCGACGAATGT		
rstB-F	CTGGGGTAAGACGCTGAAA	263 bp	qRT-PCR
rstB-R	GGGAAATGGCAATAAAAGC		
rstA-F	CGGACCGATTGTTCTTCTA	159 bp	qRT-PCR
rstA-R	GTCACTGTGGCTTGCTCAT		
slyA-F	TTGCGACTGGTCTGGAGGT	132 bp	qRT-PCR
slyA-R	GGTTCTGATCTGGCACGGT		
sdhB-F	ACACCCTGGAAGCGGAAGA	186 bp	qRT-PCR
sdhB-R	GCTGGTTGAGTGCCGAAAT		
treB-F	TGCTTGCGGCTGGAAATGAT	314 bp	qRT-PCR
treB-R	GTGCCCGTCTGTTCGCTGAT		
trpE-F	CTTCATCGCTCTGGTTACAT	119 bp	qRT-PCR
trpE-R	AAAACAACGTCTCACTGCTC		
ybjX-F	TAATGGCGTTCAGCATCACA	281 bp	qRT-PCR
ybjX-R	ACGATGTTTATTGGCGGACT		
ybtA-F	GCAAACGCAATCTGAAATCT	235 bp	qRT-PCR
ybtA-R	GAGTCCCTGAATCGCAAAGC		
wecC-F	CTGCCGTAGAAGGCGGTTTT	187 bp	qRT-PCR
wecC-R	GGCGAGGTGGATTCAAGGAT		
16S rRNA-F	GTAGTCCACGCTGTAAACGA	173 bp	qRT-PCR
16S rRNA-R	GAATTAAACCACATGCTCCA		
PfimA-F	5′-CY3-TCCATGTGTTAAGAGGATAAGCGGGGAAACCG	32 bp probe	EMSA
PfimA-R	5′-CY3-CGGTTTCCCCGCTTATCCTCTTAACACATGGA		
mPfimA-F	5′-CY3-TCCATGTG∆AGCGGGGAAACCG	21 bp probe	EMSA
mPfimA-R	5′-CY3-CGGTTTCCCCGCT∆CACATGGA		
uPfimA-F	5′-TCCATGTGTTAAGAGGATAAGCGGGGAAACCG	32 bp probe	EMSA
uPfimA-R	5′-CGGTTTCCCCGCTTATCCTCTTAACACATGGA		
PtnaB-F	5′-CY3-GCGGCGAATATTAAAGGGTTAACCTTTACCTA	32 bp probe	EMSA
PtnaB-R	5′-CY3-TAGGTAAAGGTTAACCCTTTAATATTCGCCGC		
mPtnaB-F	5′-CY3-GCGGCGAATAAA∆AACCTTTACCTA	24 bp probe	EMSA
mPtnaB-R	5′-CY3-TAGGTAAAGGT∆TTTTATTCGCCGC		
uPtnaB-F	5′-GCGGCGAATATTAAAGGGTTAACCTTTACCTA	32 bp probe	EMSA
uPtnaB-R	5′-TAGGTAAAGGTTAACCCTTTAATATTCGCCGC		

F: forward primer. R: reverse primer. The underlined nucleotide sequence represents the motif.

## Data Availability

The *eruA* gene data have been deposited in the NCBI database (Genbank Accession Number: MZ475355). The raw data from RNA sequencing have been deposited in the NCBI SRA database (BioProject number: PRJNA1070331).

## Supplementary Materials

The following supporting information can be downloaded at: https://www.mdpi.com/article/10.3390/biom16010152/s1, Table S1: Variations of the expression of DEGs based on RNA sequencing. Supplementary methods: The β-galactosidase activity of the ΔeruA-pMP220-P_eruA_*LacZ* bacteria was detected under acidic or oxidative stress using an ELISA assay. Figure S1: The β-galactosidase activity of the ΔeruA-pMP220-P_eruA_*lacZ* bacteria was detected under acidic or oxidative stress using an ELISA assay. Figure S2: The expression levels of some target genes of the *eruA* gene of the AIEC LF82 strain changed under acidic or oxidative stress. Figure S3: The intestinal inflammation of DSS-free or DSS-induced mice infected with the AIEC LF82 strain was compared with that of mice infected with *E. coli* DH5α. Figure S4: Graphical model representing the relationship between the *eruA*, *fimA,* and *tnaB* genes and the bacterial indole, adhesion, biofilm, indoles, colonization, stress resistance, as well as survival within macrophages. File S1: Original images in the manuscript.
