# Supplementary material for: EruA, a Regulator of Adherent-Invasive E. coli, Enhances Bacterial Pathogenicity by Promoting Adhesion to Epithelial Cells and Survival Within Macrophages
Source: Biomolecules. 2026 Jan 14;16(1):152. doi: 10.3390/biom16010152 (PMC12839154; doi:10.3390/biom16010152)
Supplement: Supplementary file 1 [file biomolecules-16-00152-s001.zip › Supplement material.pdf]

1.The β-galactosidase activity of the ΔeruA-pMP220-P$_{eruA}$*LacZ* bacteria was detected under acidic or oxidative stress using an ELISA assay

Methods

1.1. Constructing a plasmid pMP220-P$_{eruA}$*LacZ* and produced the ΔeruA-pMP220 -P$_{eruA}$*LacZ* bacteria

A fragment of the promoter of the *eruA* gene was amplified with primers designed according to the genomic sequence of the AIEC LF82 strain (GenBank accession no: NC_011993. The 356 bp fragment was inserted into a pMP220 vector to construct a recombinant plasmid pMP220-P$_{eruA}$*LacZ*. The plasmid was introduced into the ΔeruA bacteria by $CaCl_2$ method. The ΔeruA-pMP220-P$_{eruA}$*LacZ* bacteria were produced.

1.2. Preparing bacterial samples

The logarithmic phase ΔeruA-pMP220 -P$_{eruA}$*LacZ* bacteria were cultured in 5 mL LB broth (pH 3.9, 4.3, 4.7, 5.1, 5.5, 5.9, 6.3, 6.7 or 7.1), or in LB broth with various concentrations of H_2O_2 (0, 0.5, 1.0, 2.0, 3.0 or 4.0 mM) for 2 hr at 37°C. After centrifuging the bacterial culture at 12,000 rpm for 10 min at 4°C, the precipitate was washed twice with PBS. After treating the bacteria with an ultrasonic power of 1200 W for 8 min with Plus 5s/10s on ice and centrifuging at 13000 rpm for 15 min at 4°C, the supernatant was collected and was stored at -20°C.

1.3. Detecting the activity of bacterial β-galactosidase (β-gal) using an ELISA assay

According to the manufacturer's instructions of the β-gal-linked ELISA assay kit (Mlbio, Shanghai, China), an β-gal standard curve indicating the relationships between a different concentrations of β-gal standard and the corresponding spectrophotometric absorbances (OD_{450}) were established. After added 40 μL diluent and 10 μL sample to each well in coated 96-well plate, mixed and incubated for 30 min at 37°C. After washing with scrubbing solution, 50 μL enzyme-labeled antibody was added to each well and incubated for 30 min at 37°C. After washing with scrubbing solution, 50 μL color developer A and 50 μL color developer B were added to each well. After mixing gently, incubated for 15 min at 37°C in the dark. The absorbance at OD_{450} was measured by ultraviolet spectrophotometer. The relative

β-gal concentrations were calculated from the indole standard curve.

2. Figure S1. The β-galactosidase activity of the ΔeruA-pMP220-P_{eruA}*LacZ* bacteria was detected under acidic or oxidative stress using an ELISA assay. (**A**) The activity of the bacterial β-galactosidase was measured under LB medium with the different pH. (**B**) The activity of the bacterial β-galactosidase was measured under LB medium with the different concentrations of H_2O_2.

3.Figure S2. The expression levels of some target genes of the *eruA* gene of AIEC LF82 strain changed under acidic or oxidative stress. (**A**) The expression levels of the *eruA* gene in the WT bacteria in acidic LB medium (pH 5.8) were compared with those in standard LB medium (pH 7.4) based on qRT-PCR analysis. (**B**) The expression levels of the *eruA* gene in the WT bacteria in oxidative LB medium (2 mM H_2O_2) were compared with those in standard LB medium (0 mM H_2O_2) based on qRT-PCR analysis.

3. Figure S3: The intestinal inflammation of DSS-free or DSS-induced mice infected with AIEC LF82 strain was compared with that of mice infected with *E.coli* DH5α. (**A**) Mice in the Control, LF82, DH5α, DSS, DH5α+DSS and LF82+DSS groups were orally administered 2.5% DSS or NS, and/or LF82 or DH5α bacteria for bacterial infection. (**B**) The body weights in the Control, DSS, LF82 and DH5α groups were recorded from the first day after NS or DSS was added to the drinking water. (**C**) The body weights in the DSS, LF82+DSS and DH5α+DSS groups were recorded from the first day after DSS was added to the drinking water. (**D**) The number of the LF82 and DH5α bacteria colonized in the colon of mice, either with DSS-induced colitis or DSS-free, was compared. (**E**) The entire mice colons in the six groups were harvested on Day 17th. 1-6 represent the colons in the DSS, LF82, DH5α, Control, LF82+DSS and DH5α+DSS groups, respectively. (**F**) The colon lengths in the DSS, LF82 and DH5α groups were compared with those in the Control group. (**G**) The colon lengths in the LF82+DSS and DH5α+DSS groups were compared with those in the DSS

group. (**H**) Representative pictures of H&E-stained colon tissues in the Control, LF82, DH5α, DSS, LF82+DSS and DH5α+DSS groups were shown (scale bars, 100 µm; magnification, 100 x and 400 x). (**I**) The cumulative histology scores of the colon tissues in the control group were compared with those in LF82, DH5α, DSS, LF82+DSS and DH5α+DSS groups, and the cumulative histology scores of the colon tissues in the DSS group were compared with those in the LF82+DSS and DH5α+DSS groups. Data were represented as the mean ± SD, with n = 6-8 per group. Statistical significance was assessed using a one-way ANOVA, followed by Tukey's test, *$P < 0.05$, **$P < 0.01$,***$P < 0.001$; NS, not significant.

4. Figure S4: The graphical model represented the relationship between the *eruA*, *fimA* and *tnaB* genes and the bacterial indole, adhesion, biofilm, indoles, colonization, stress resistance as well as survival within macrophages.
