# Supplementary material for: EruA, a Regulator of Adherent-Invasive E. coli, Enhances Bacterial Pathogenicity by Promoting Adhesion to Epithelial Cells and Survival Within Macrophages
Source: Biomolecules. 2026 Jan 14;16(1):152. doi: 10.3390/biom16010152 (PMC12839154; doi:10.3390/biom16010152)

Original content, all rights reserved.

EruA, a regulator of adherent-invasive *E.coli,* enhances the bacterial pathogenicity by promoting adhesion to epithelial cells and survival within macrophages

Images images

H&E staining in pathology

control group-10x

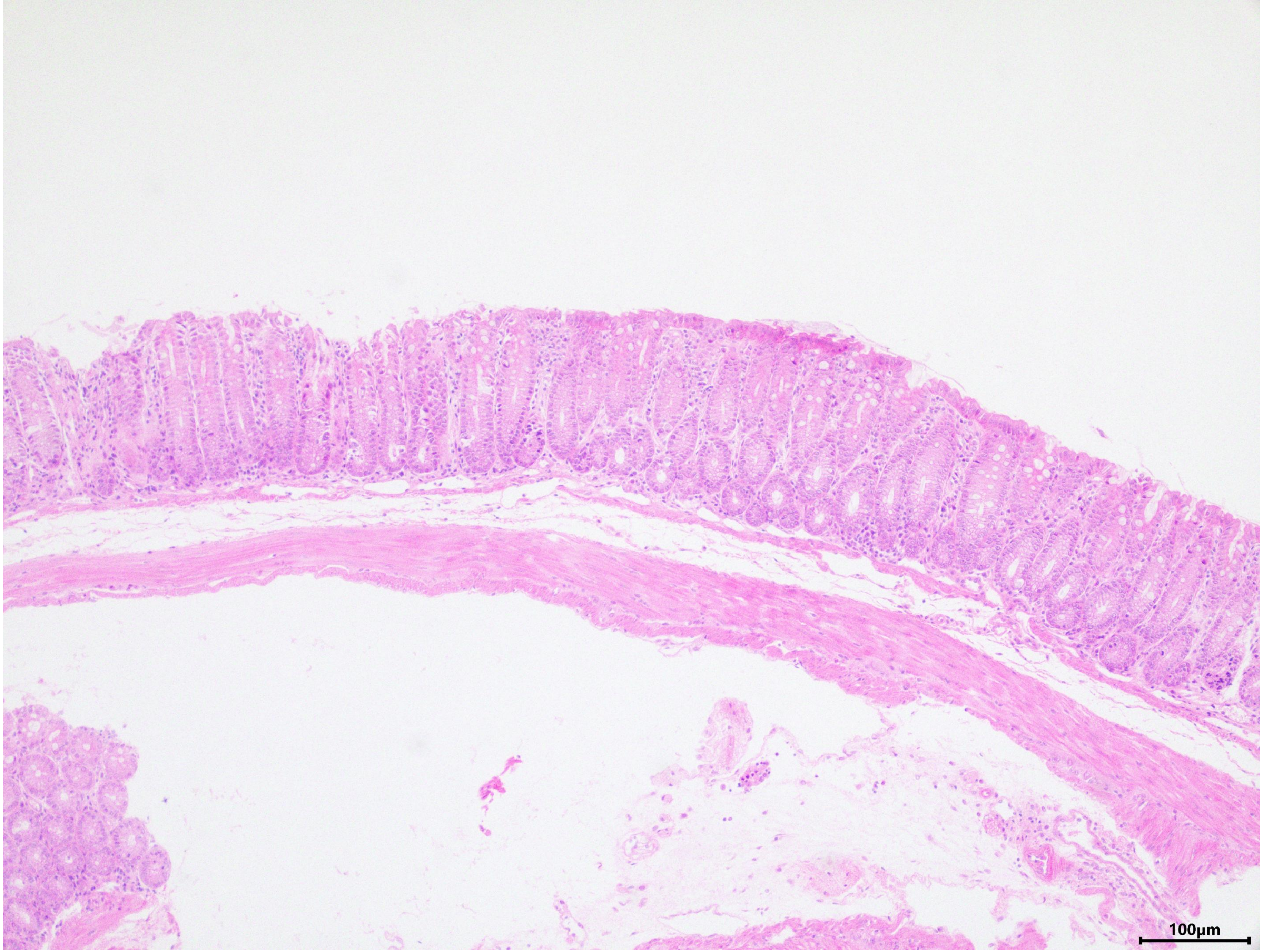

control group-40x

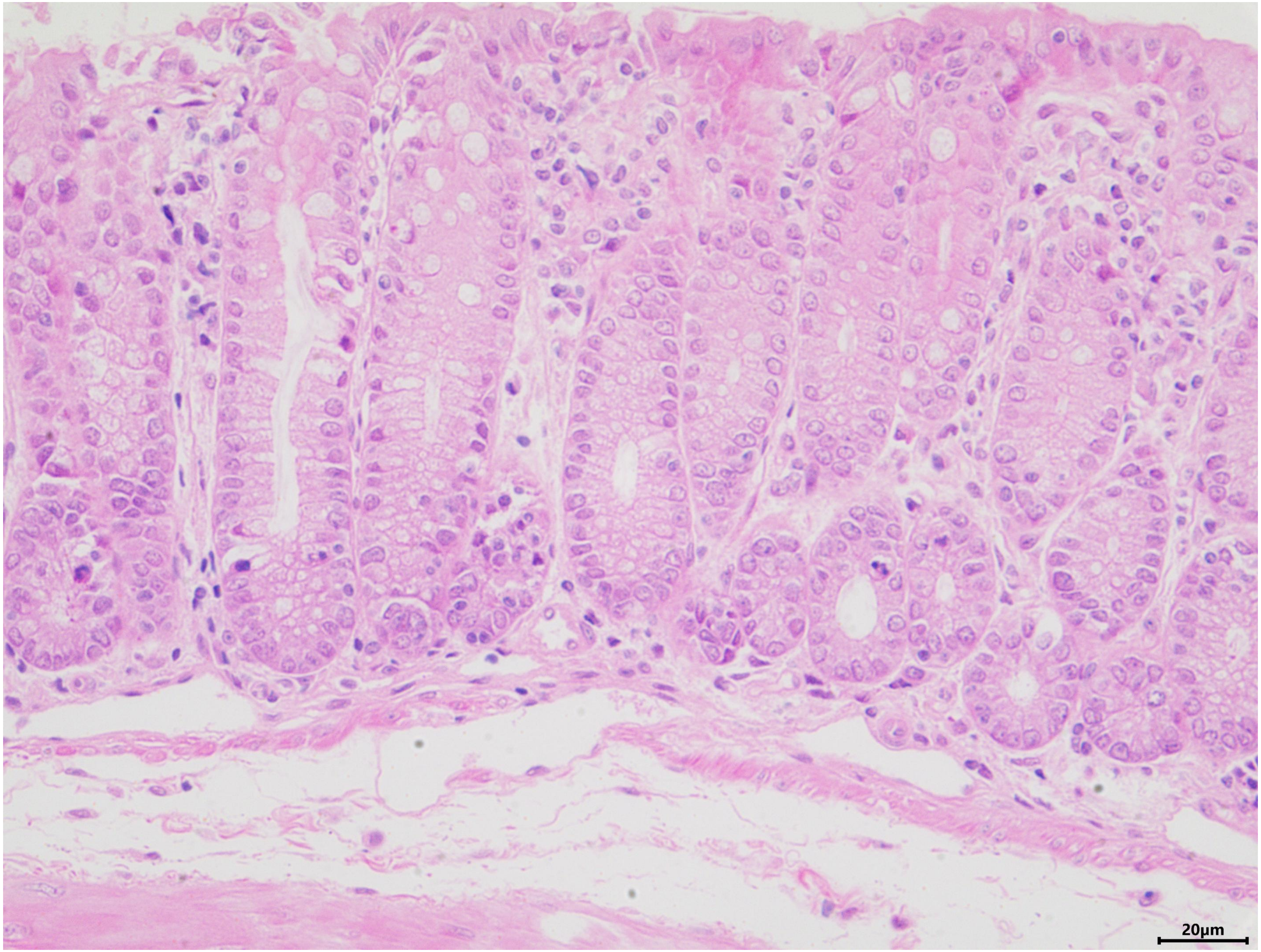

wt/LF82 group-10x

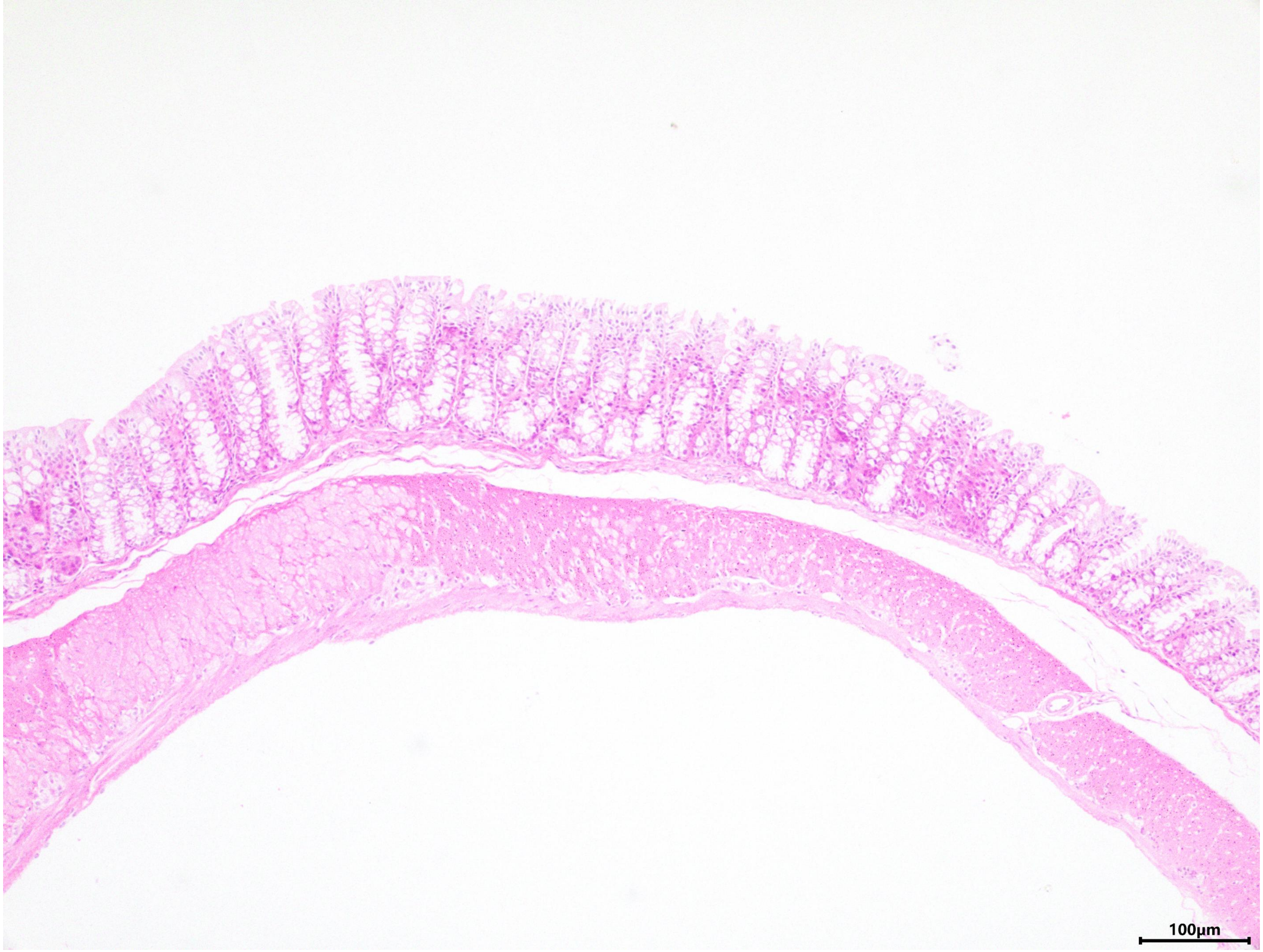

wt/LF82 group-40x

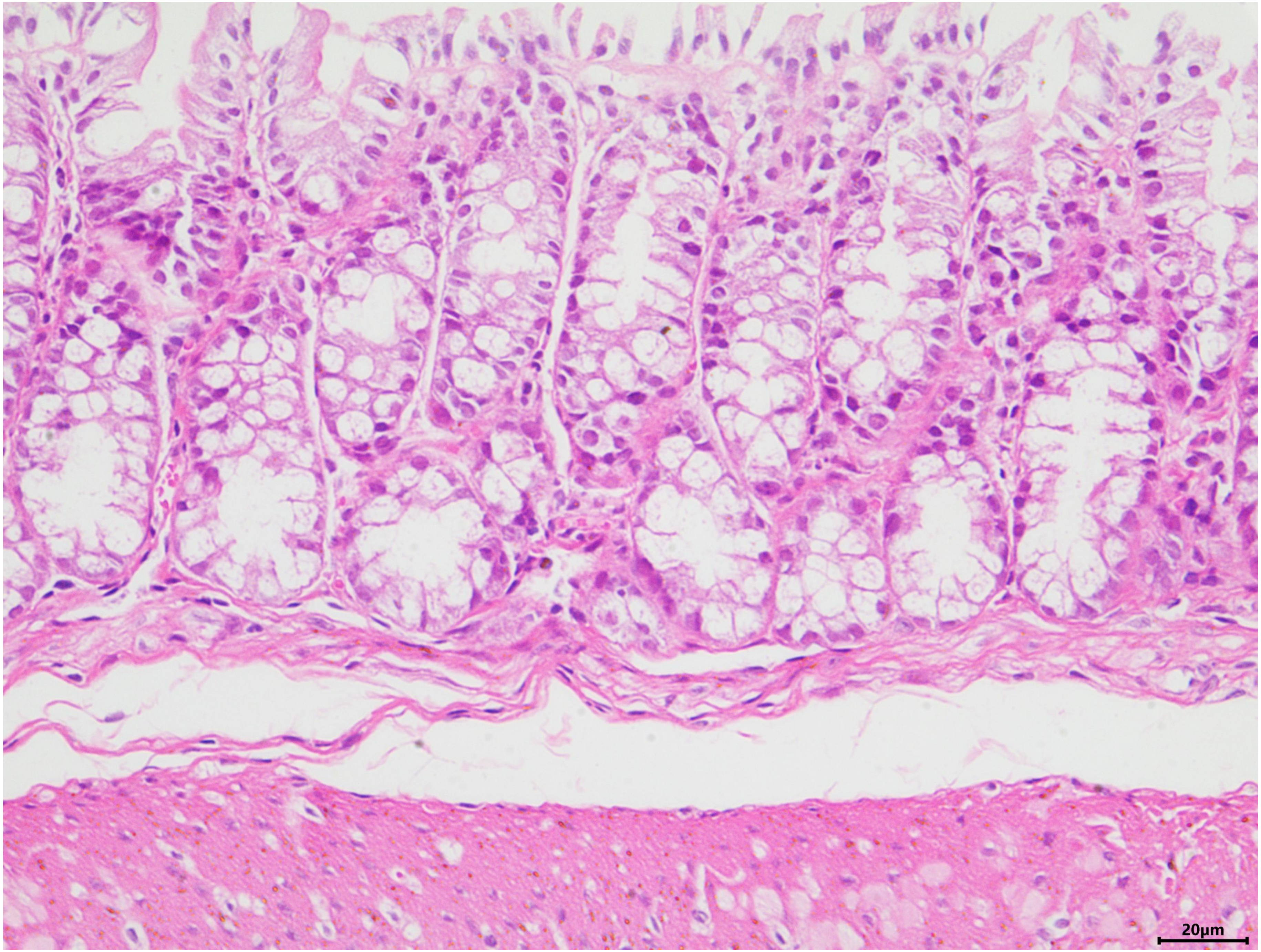

Delta erua group-10x

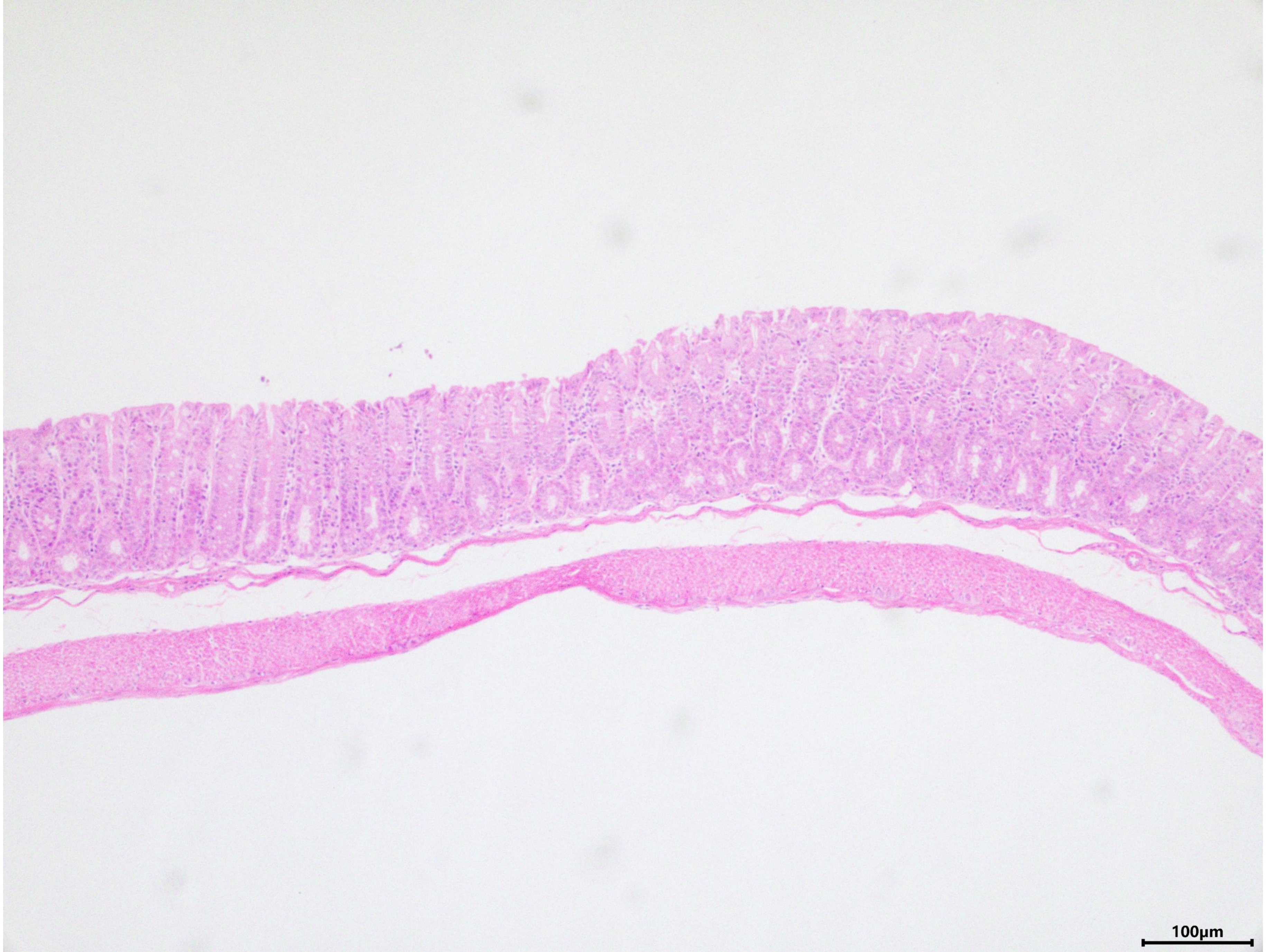

Delta erua group-40x

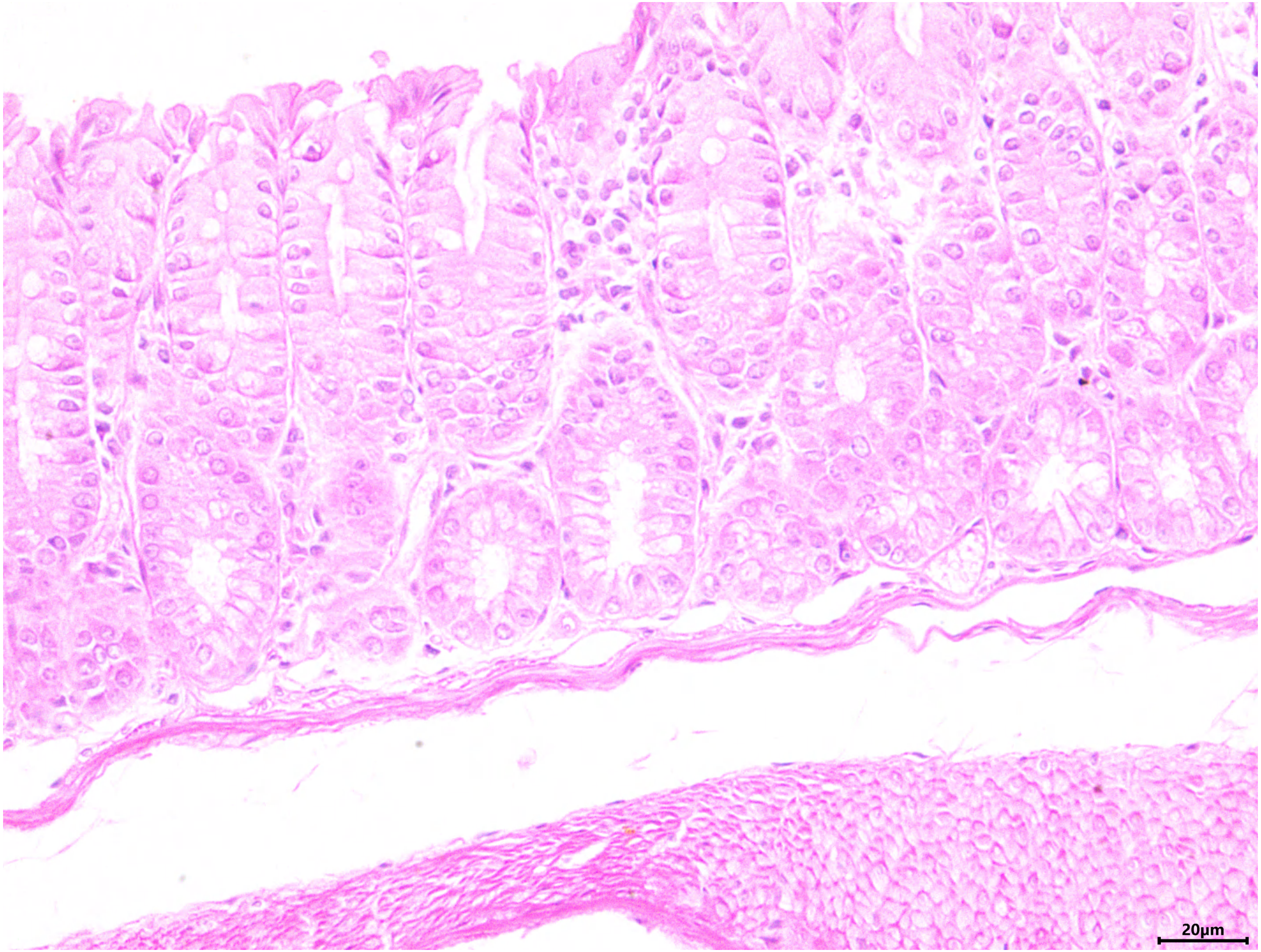

DH5a group-10x

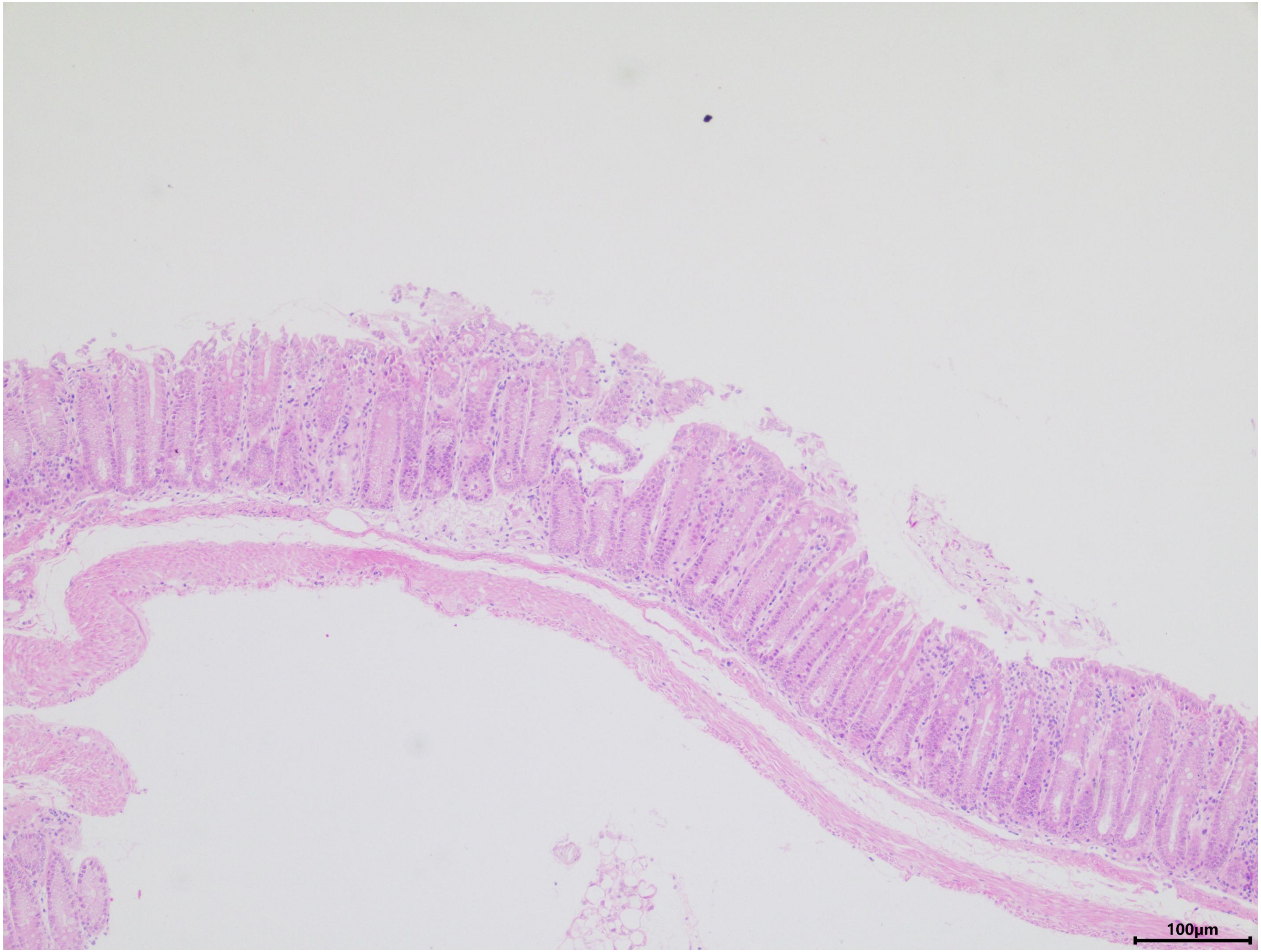

DH5a group-40x

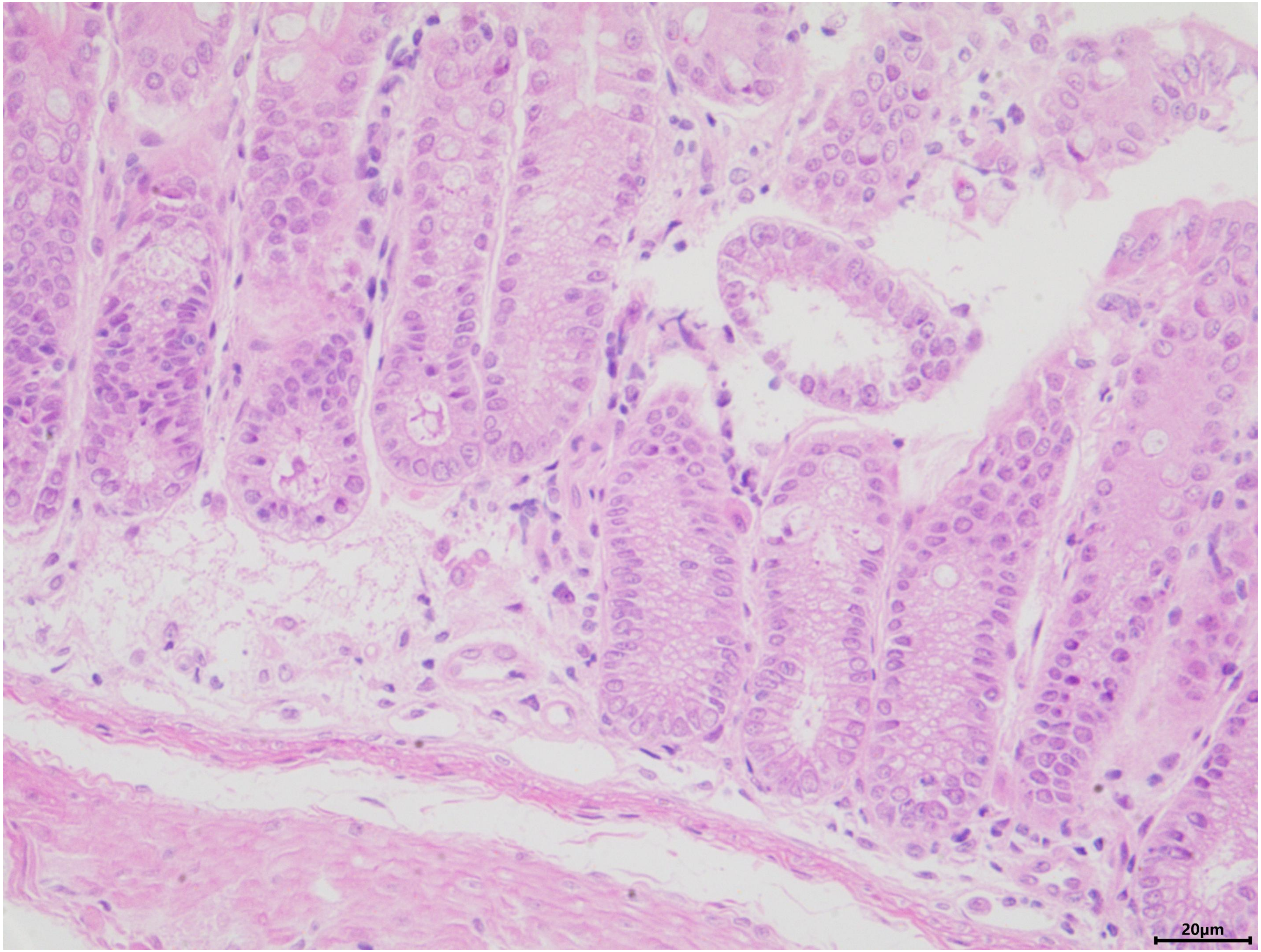

dss group-10x

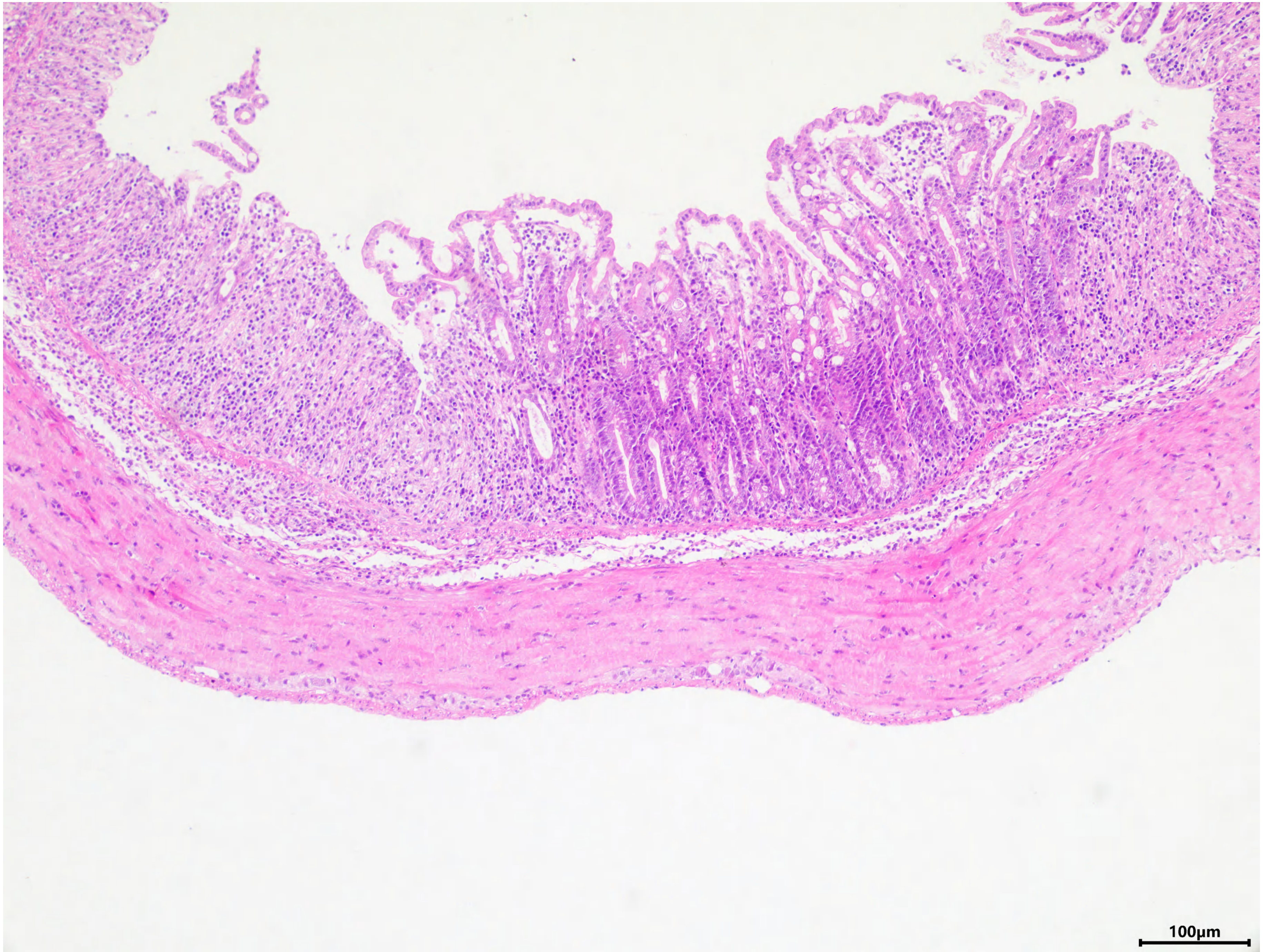

dss group-40x

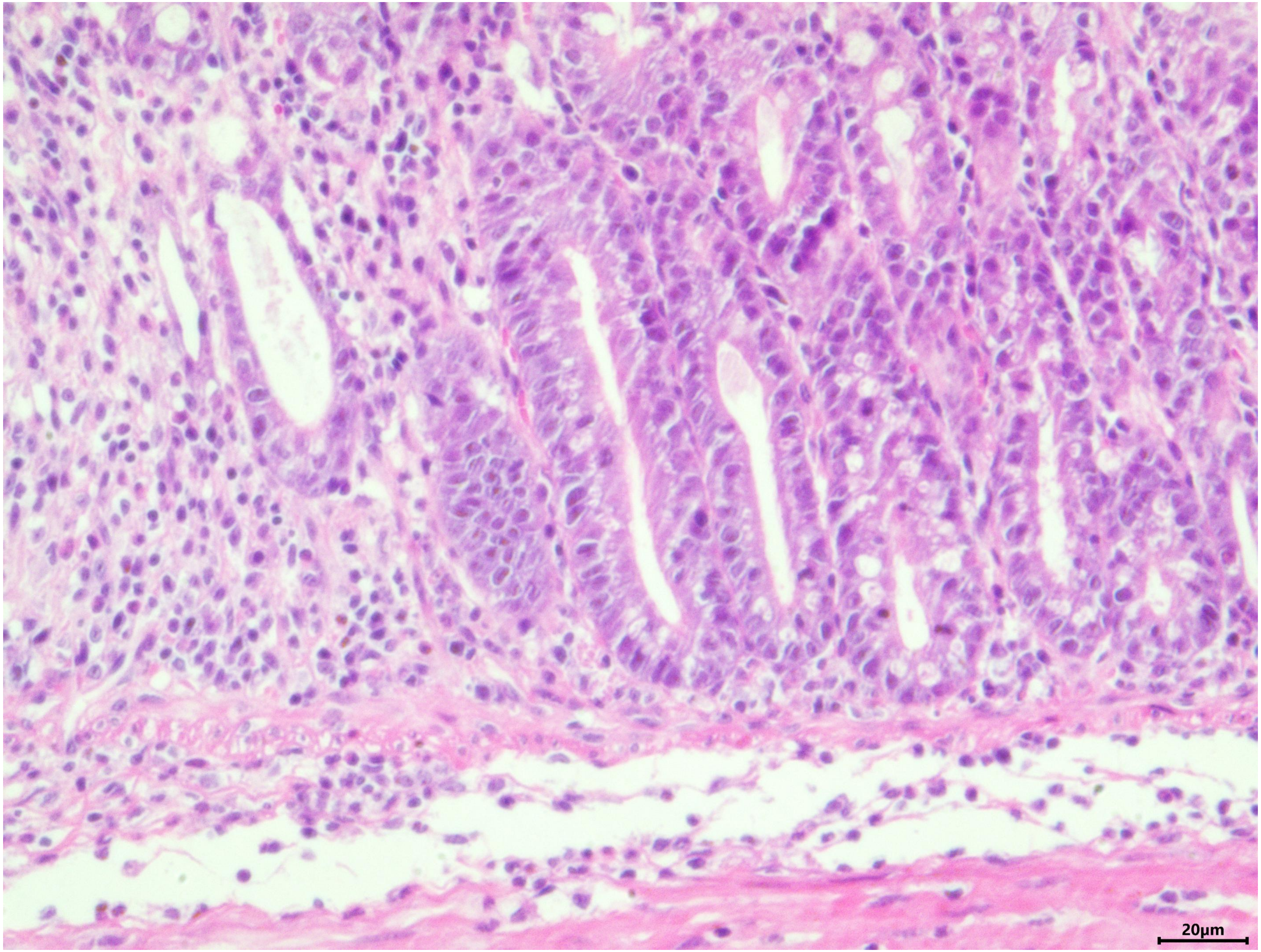

dss+wt/LF82 group-10x

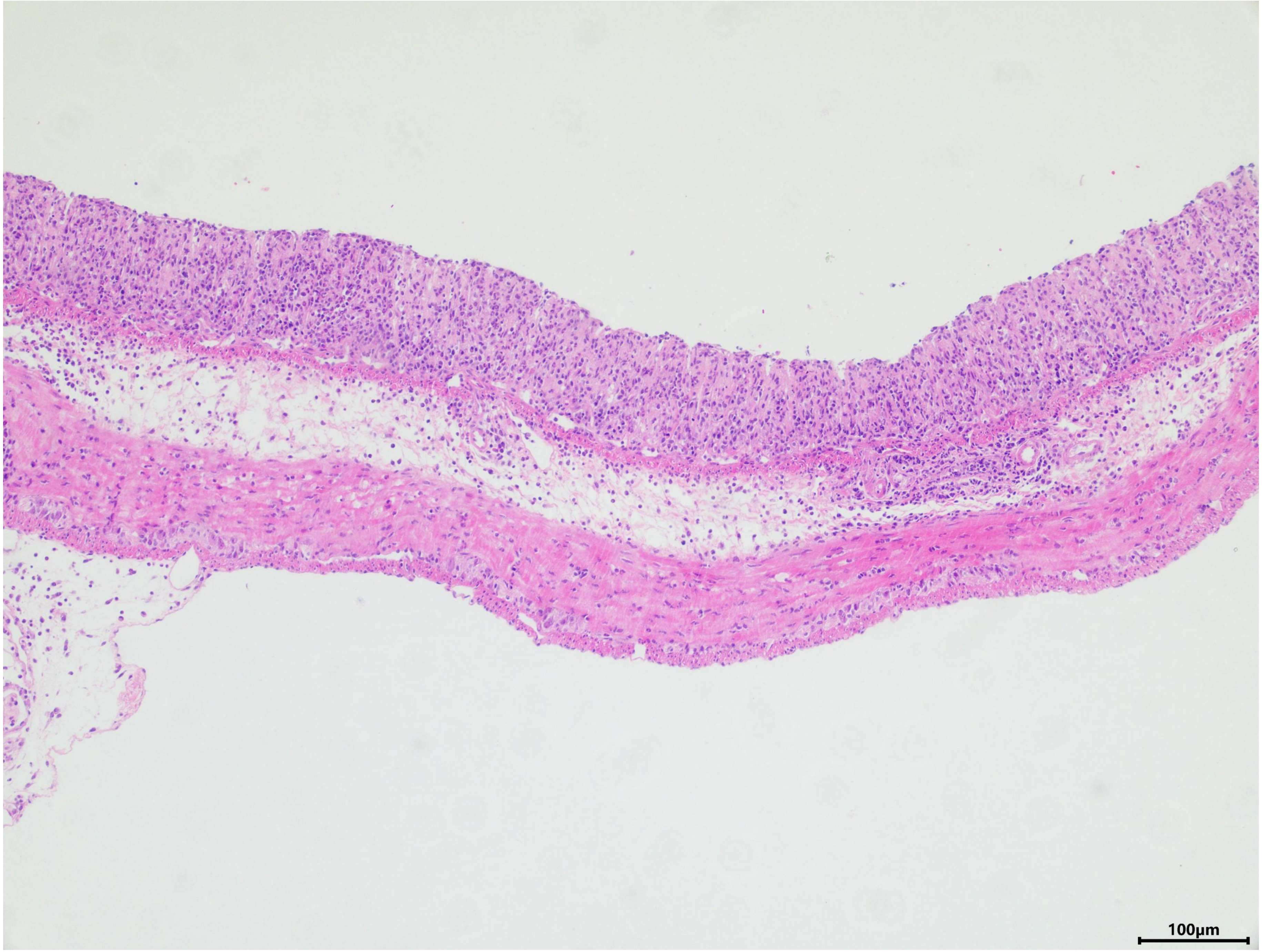

dss+wt/LF82 group-40x

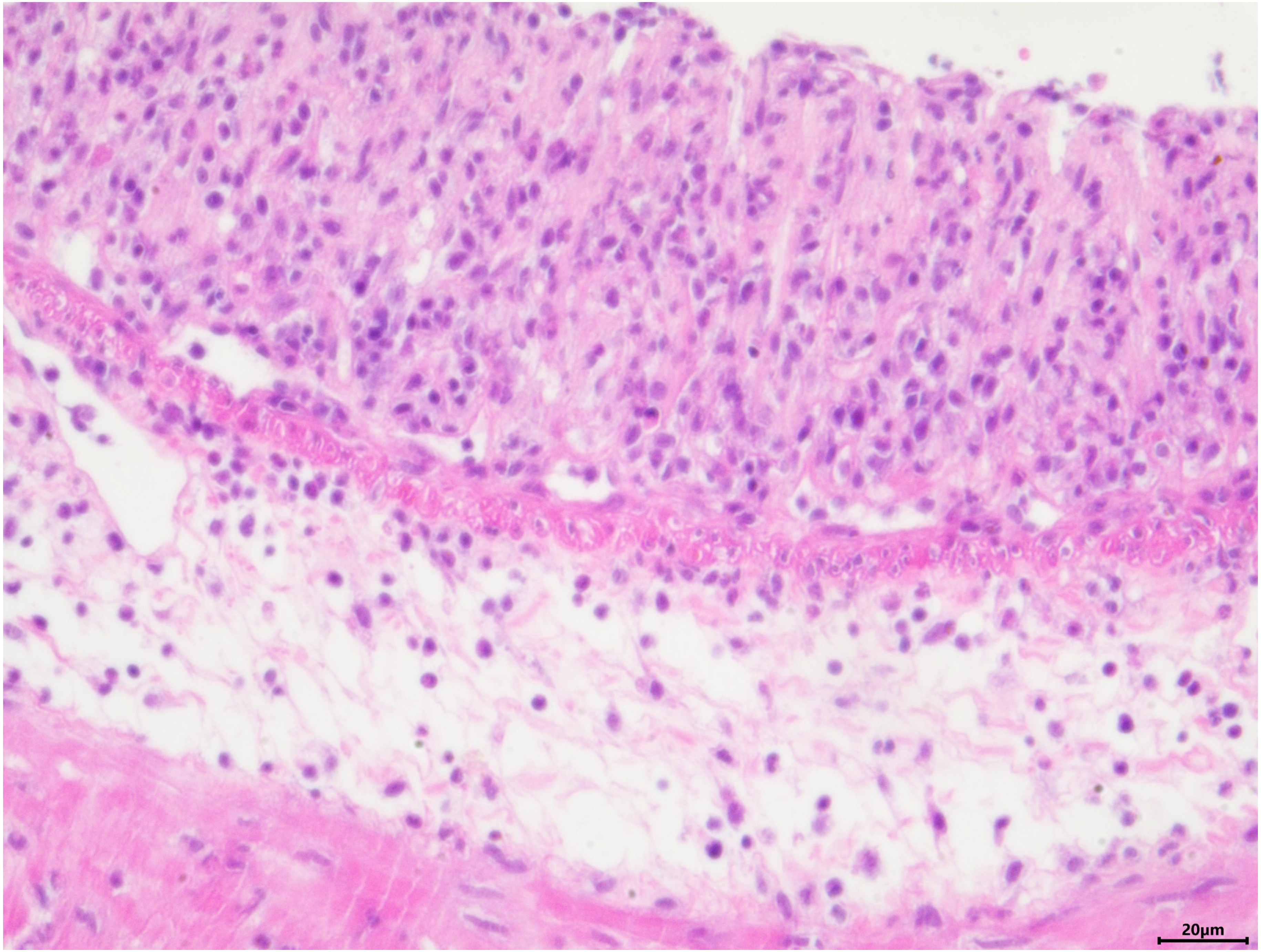

dss + delta erua group-10x

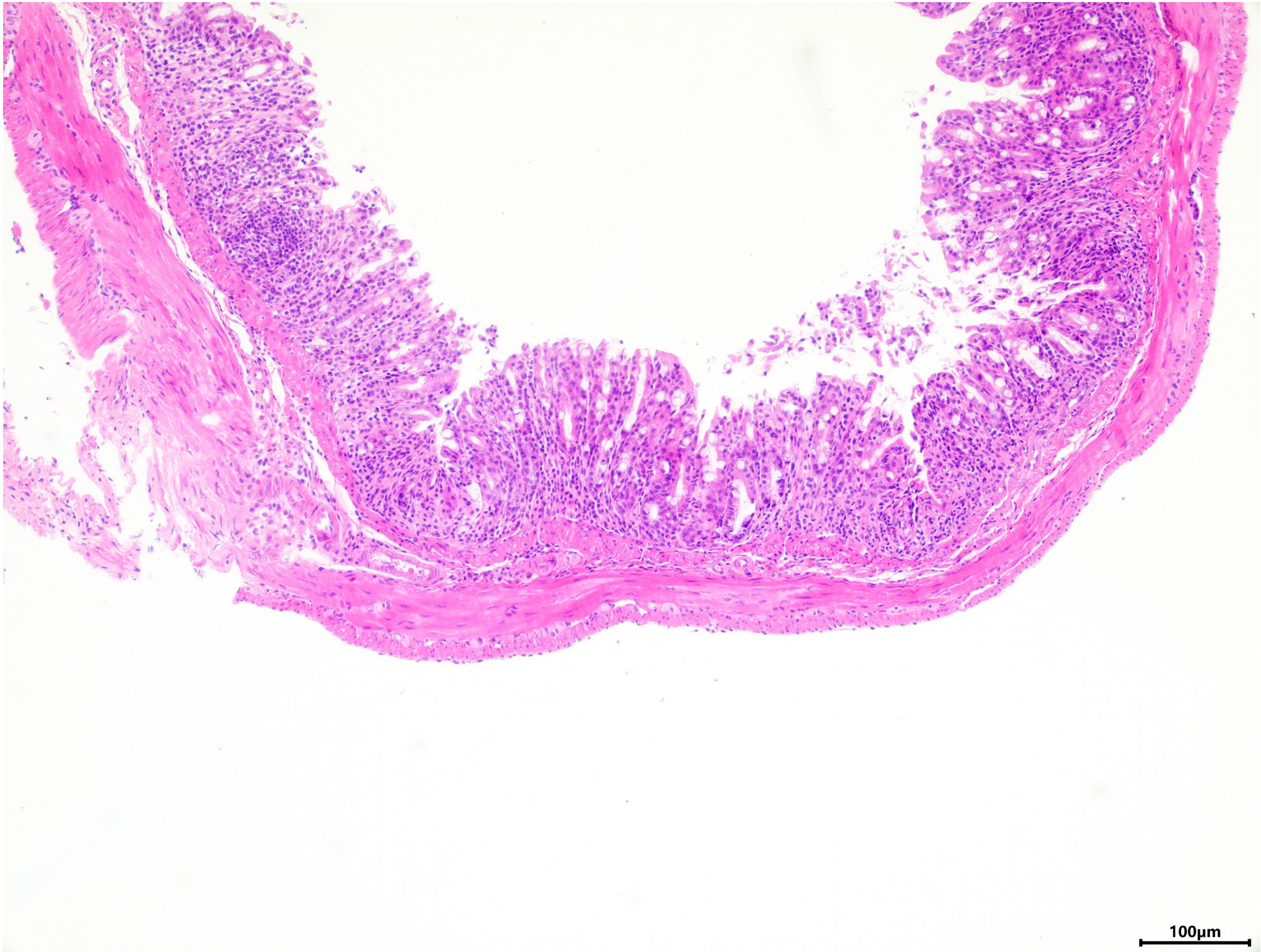

dss + delta erua group-40x

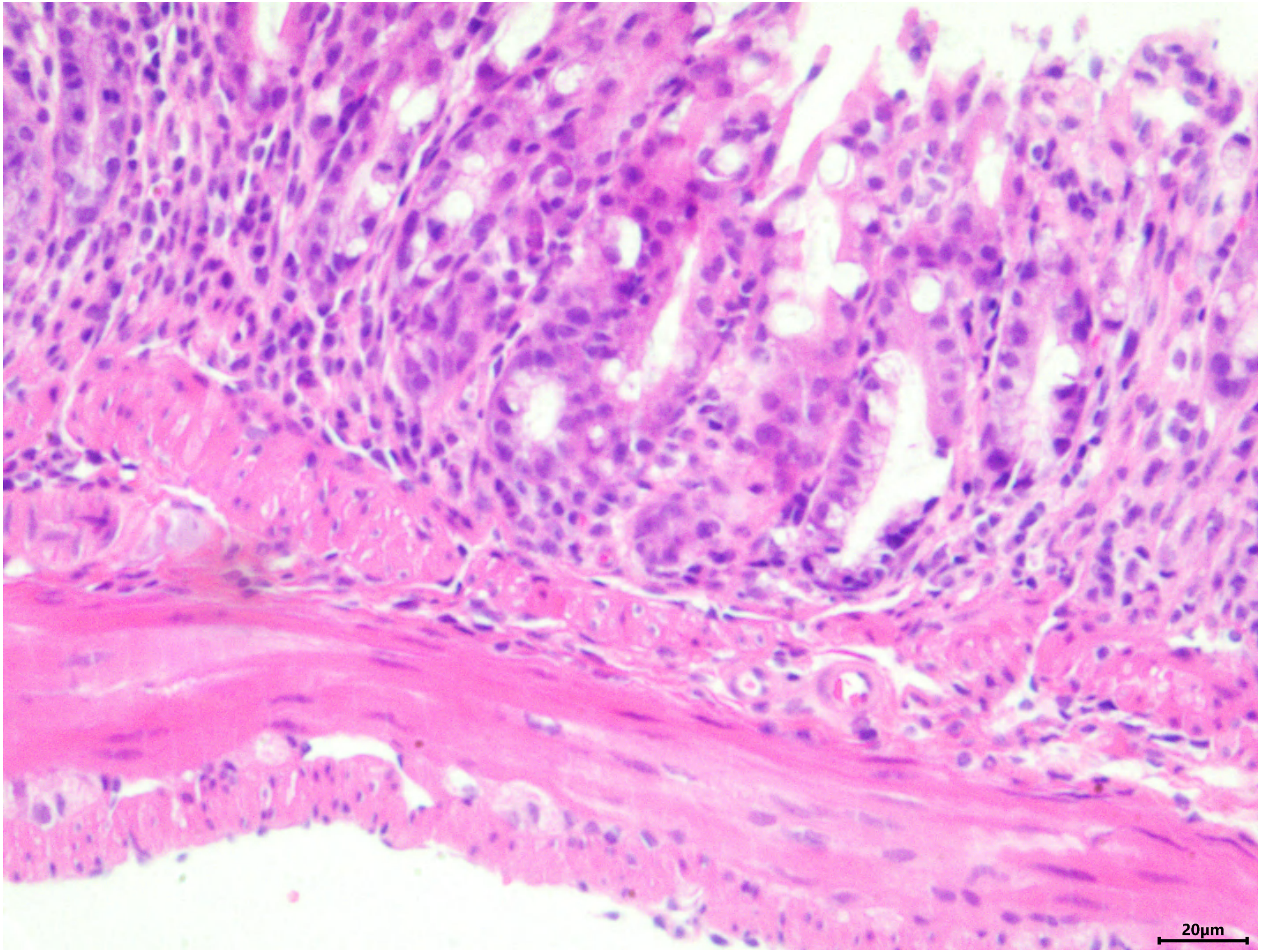

dss + DH5a group-10x

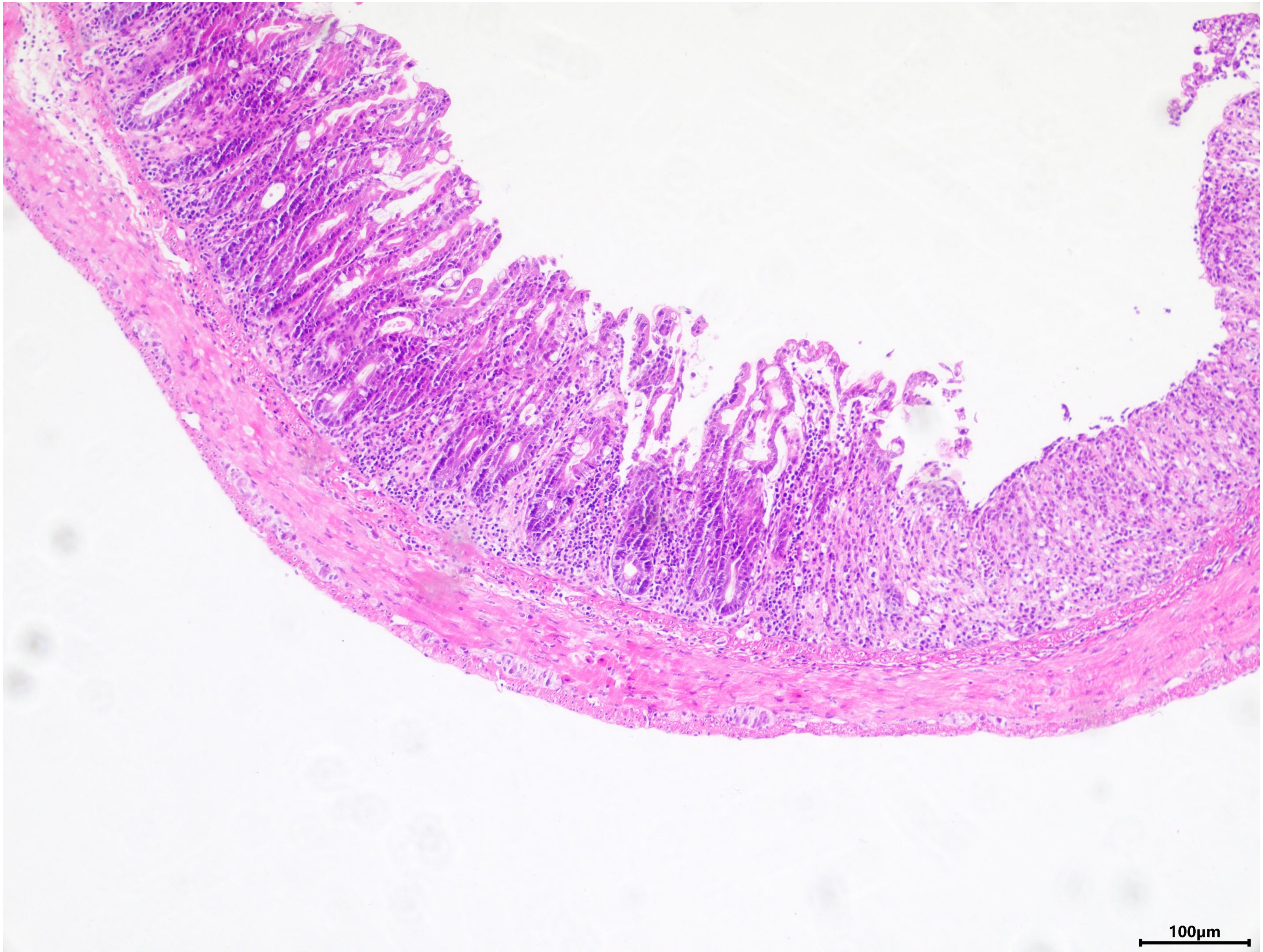

dss + DH5a group-40x

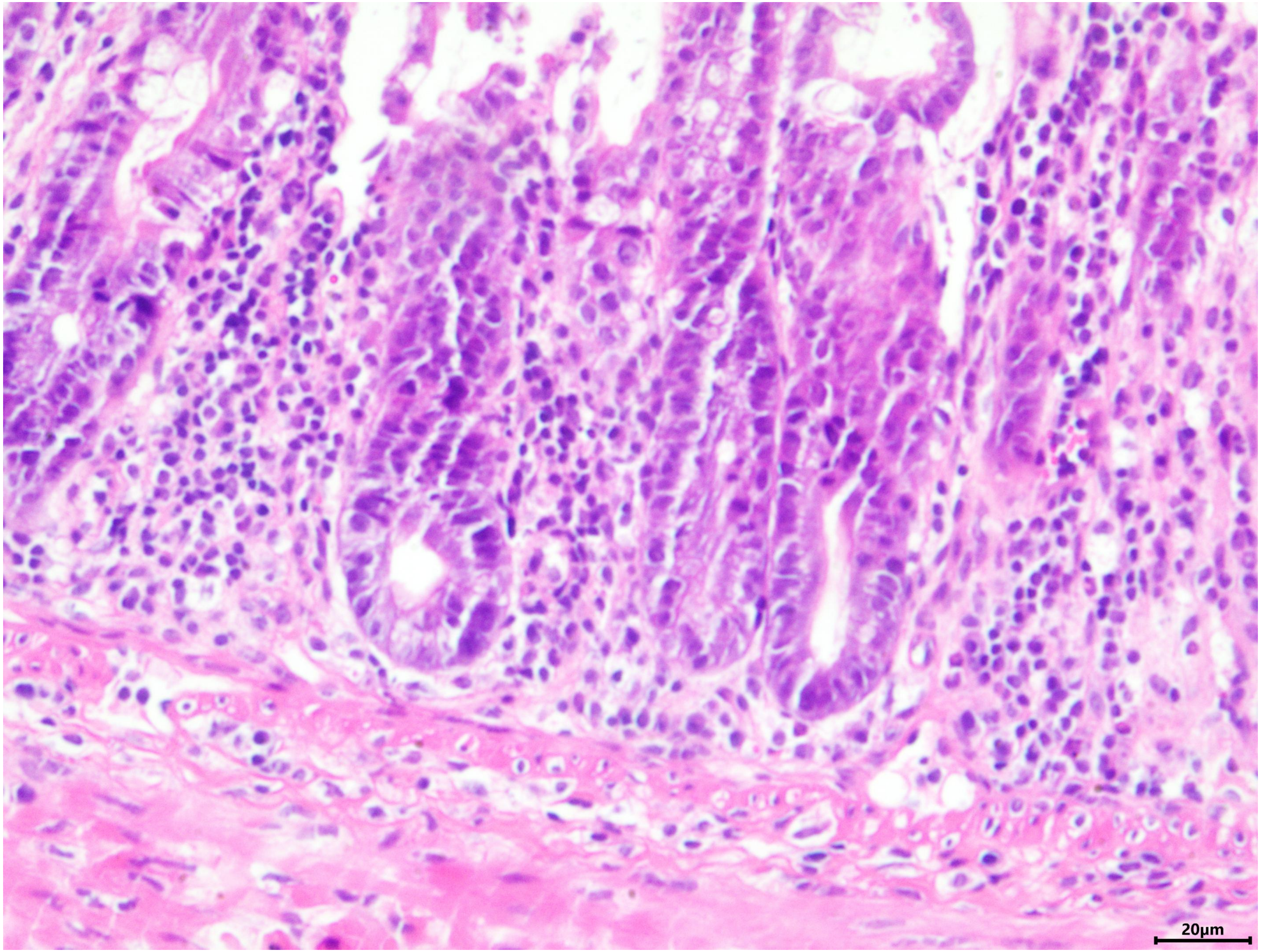

Survival
Delta erua-1h

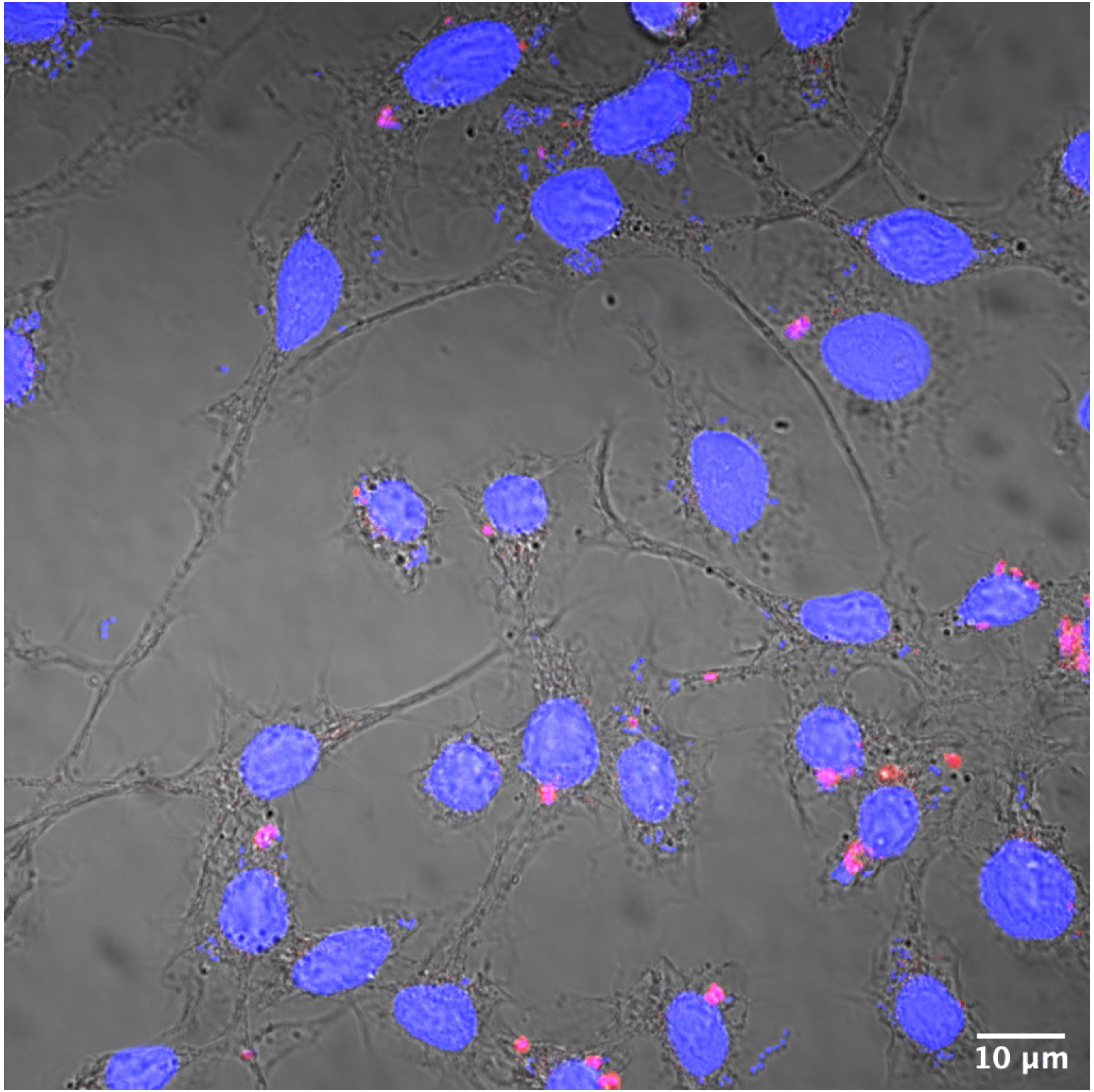

Survival
Delta erua-
6h

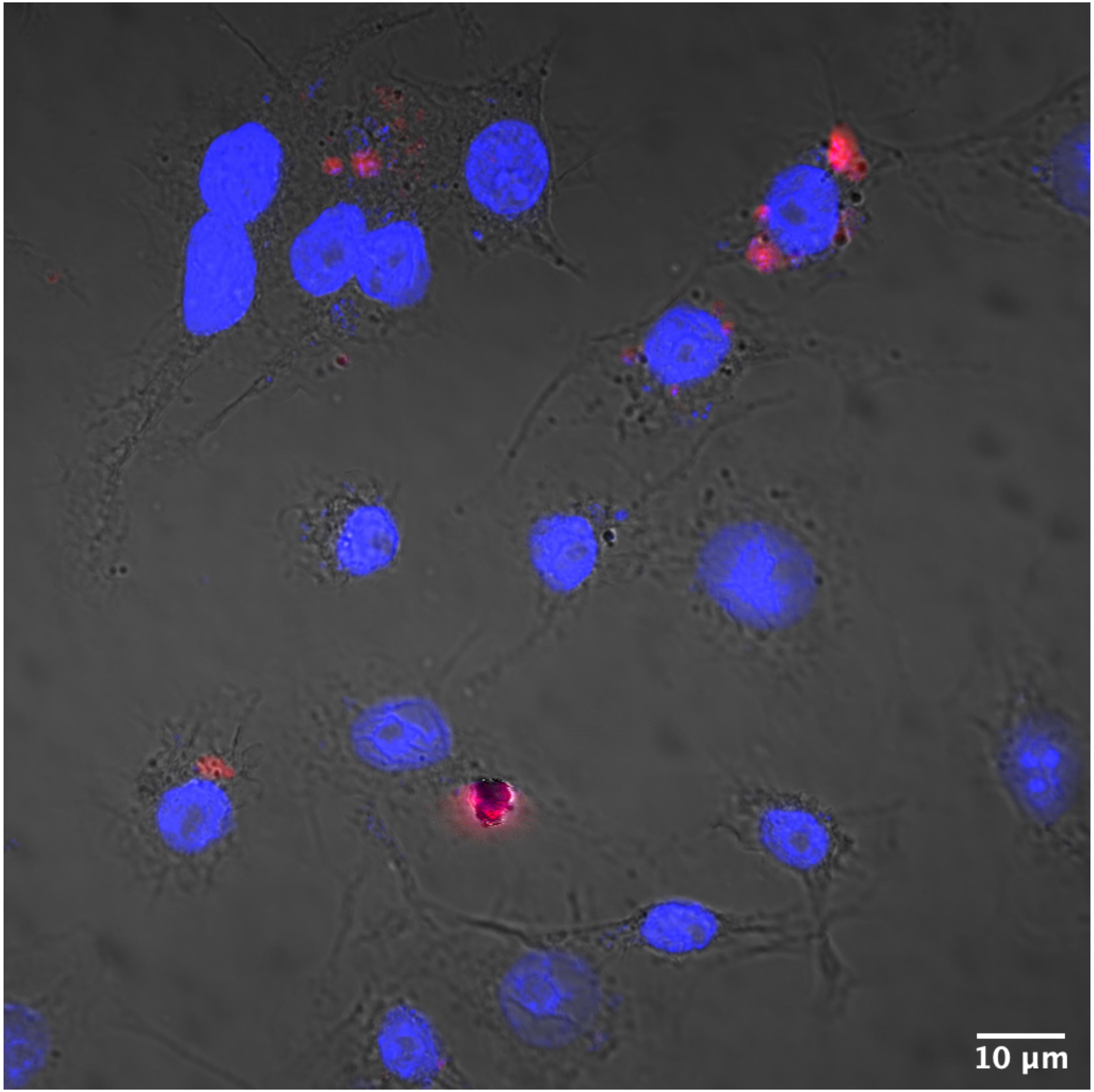

Survival

Delta erua-
18h

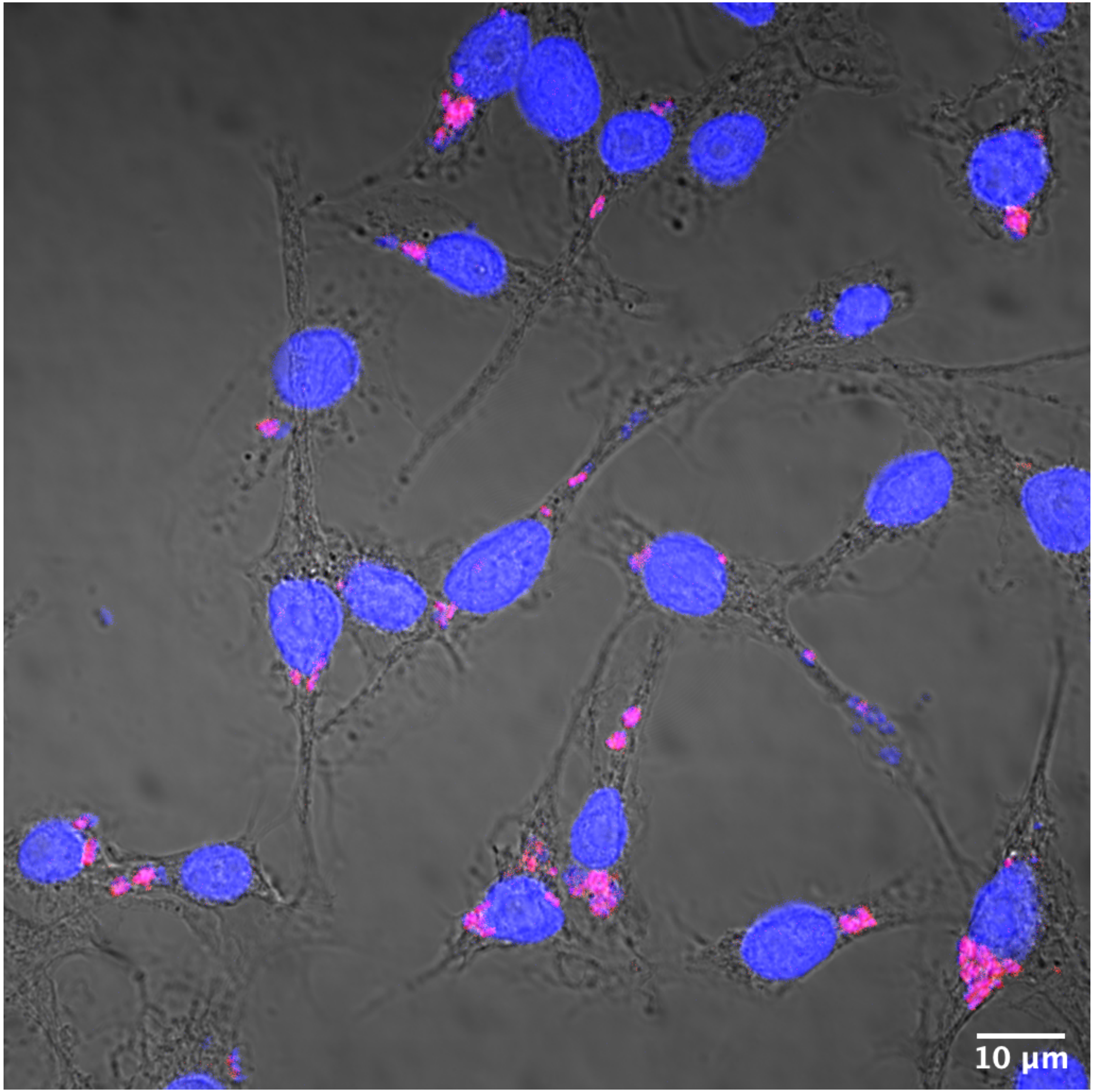

Survival
C-delta erua-
1h

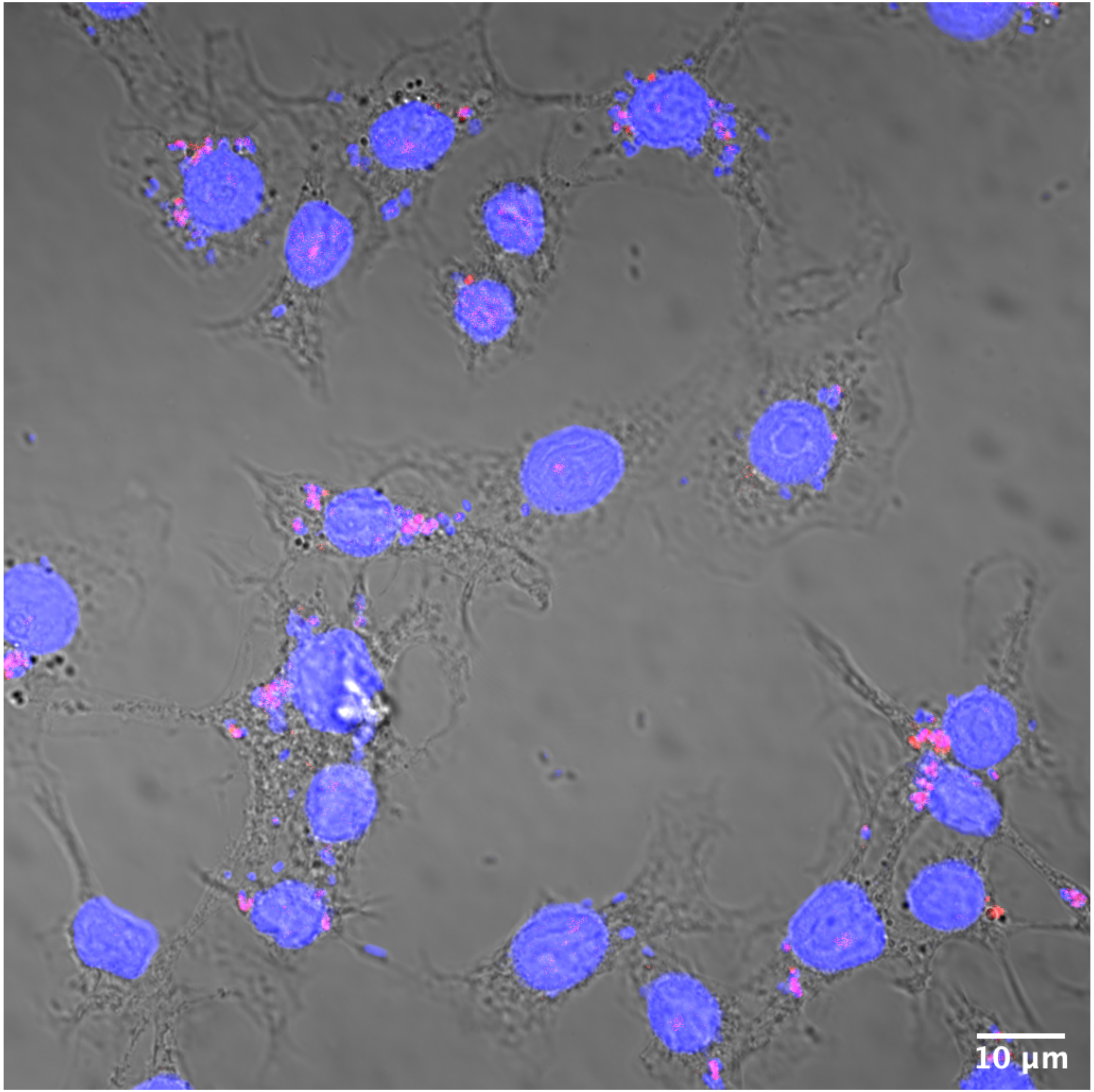

Survival
C-delta erua-
6h

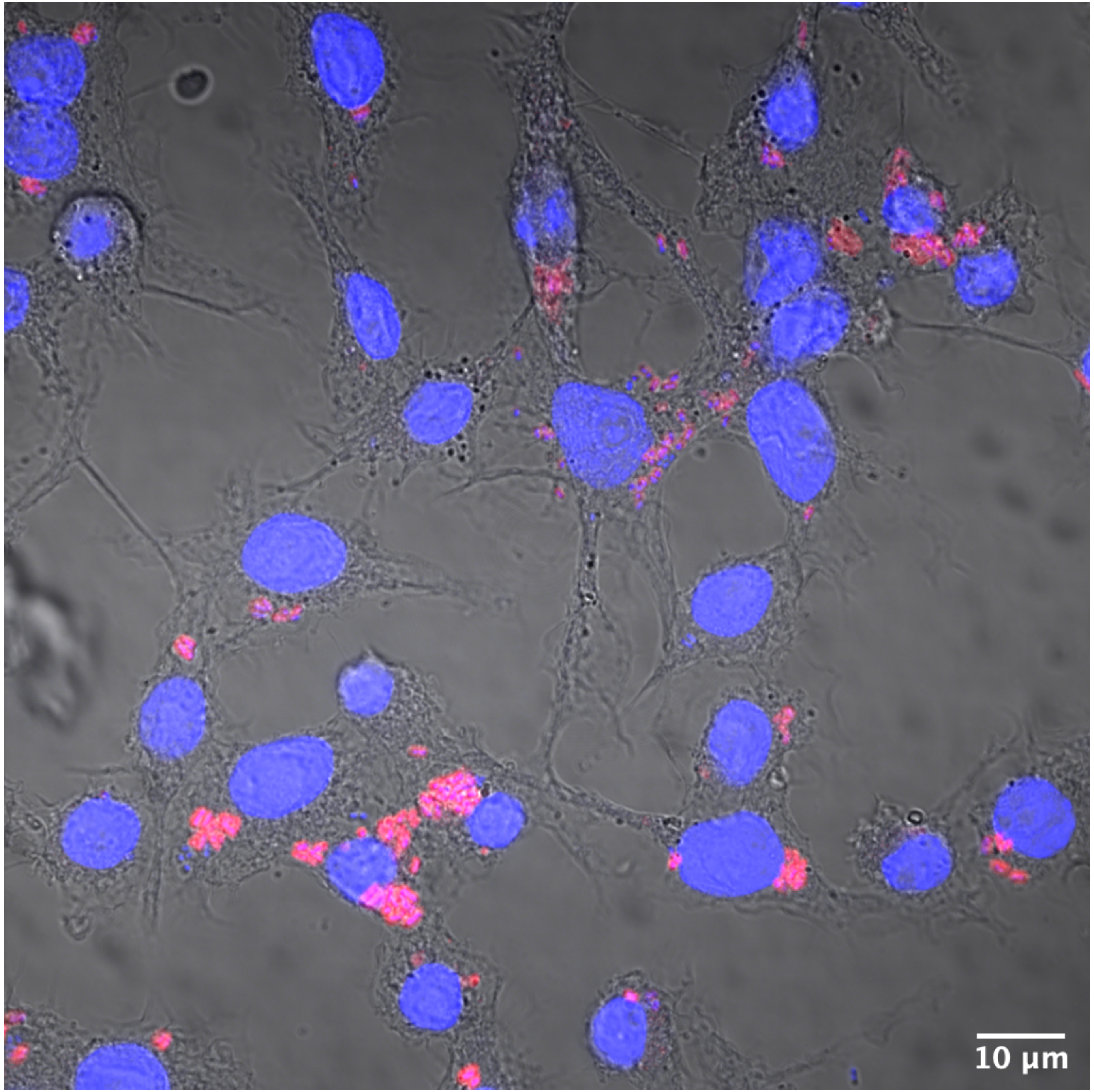

Survival
C-delta erua-18h

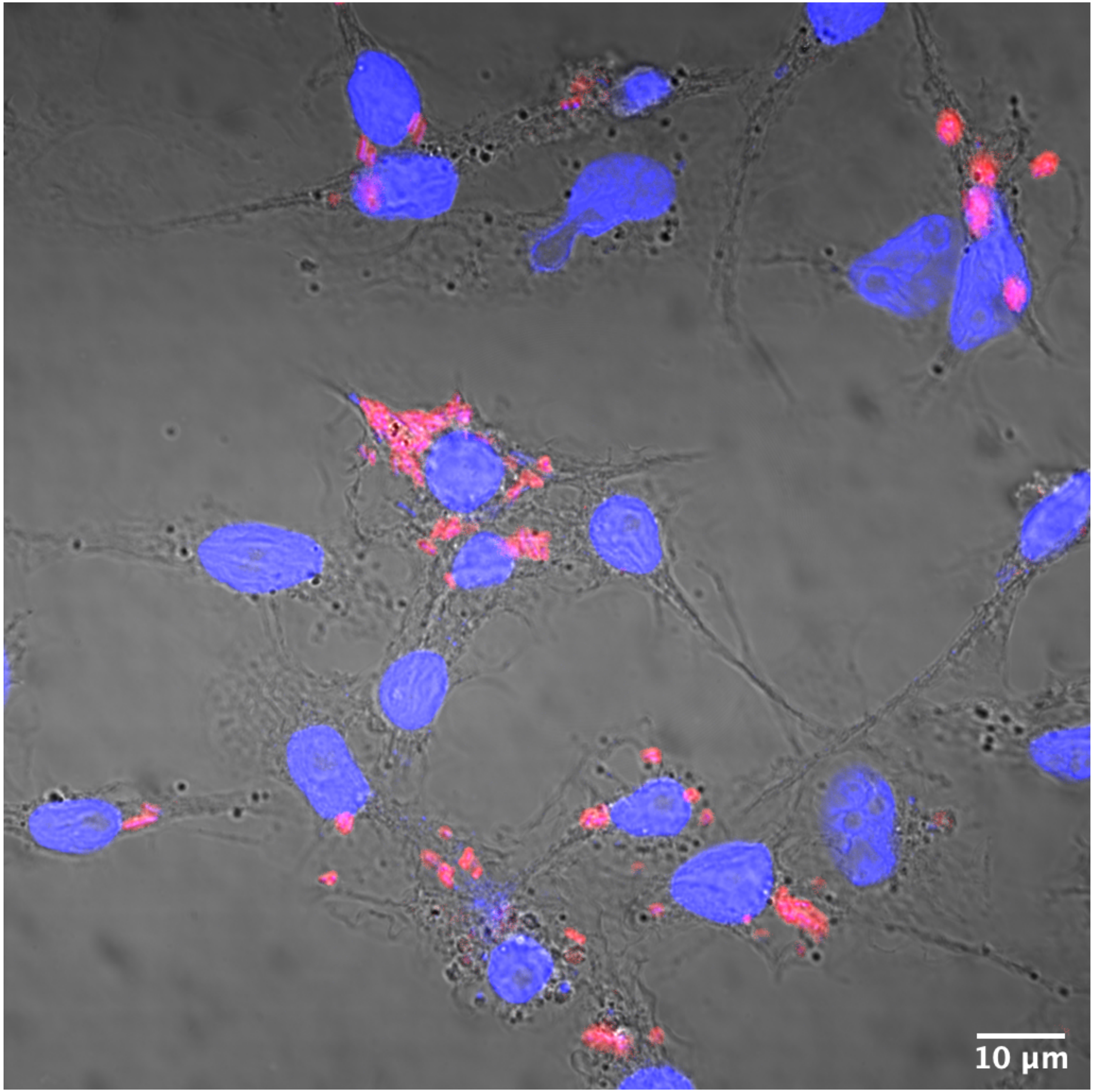

Survival
WT-1h

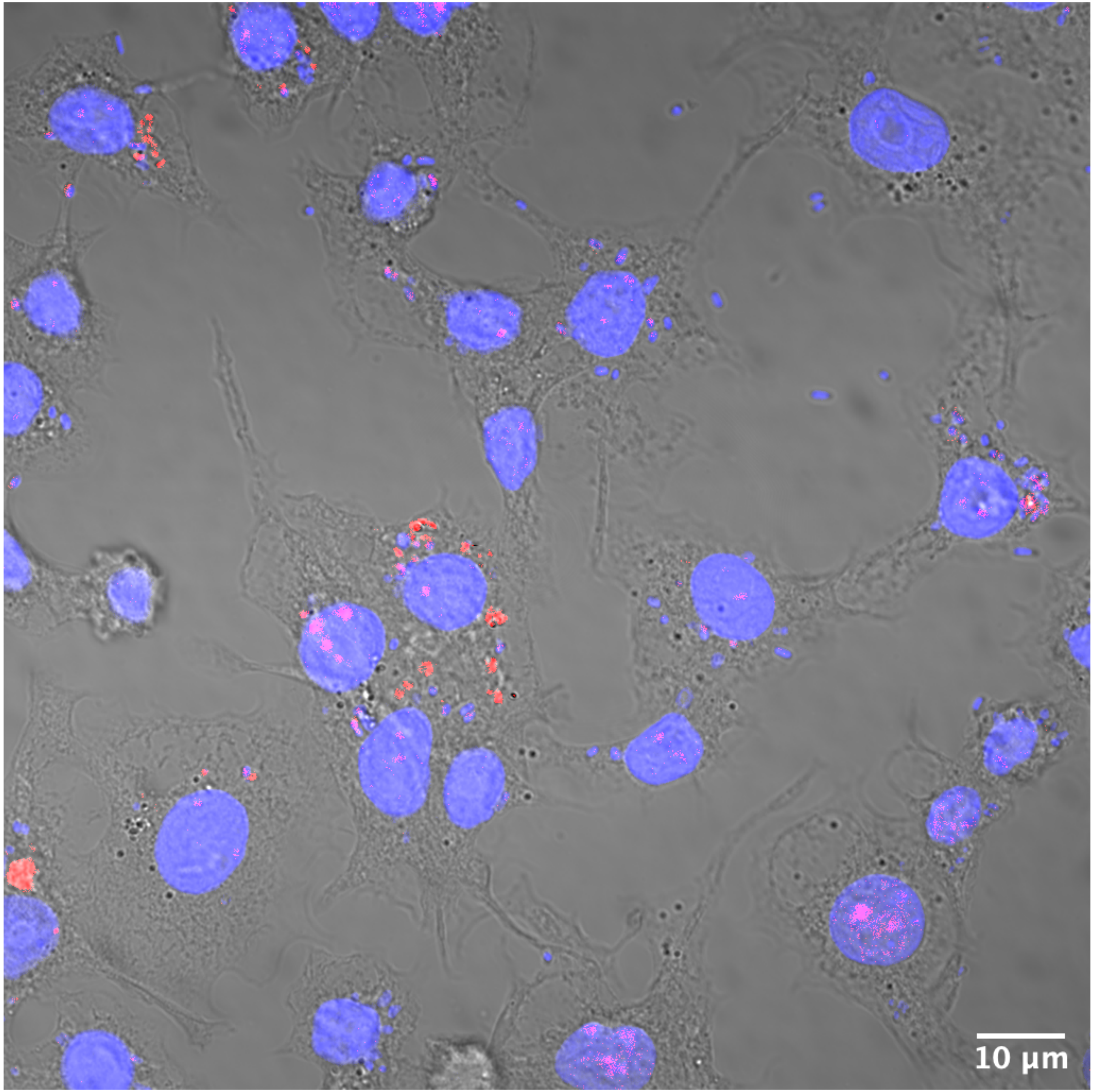

Survival
WT-6h

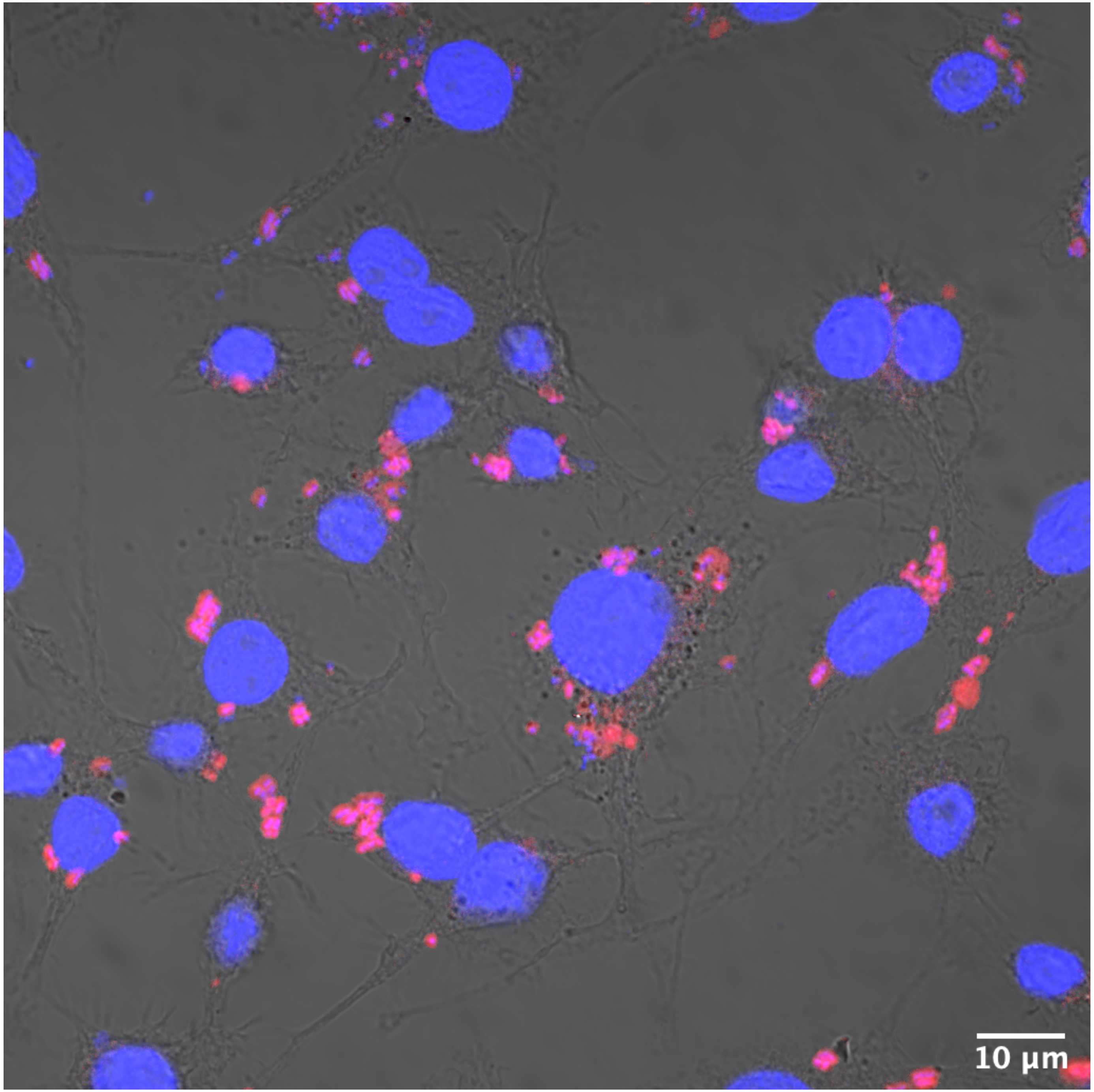

Survival
WT-18h

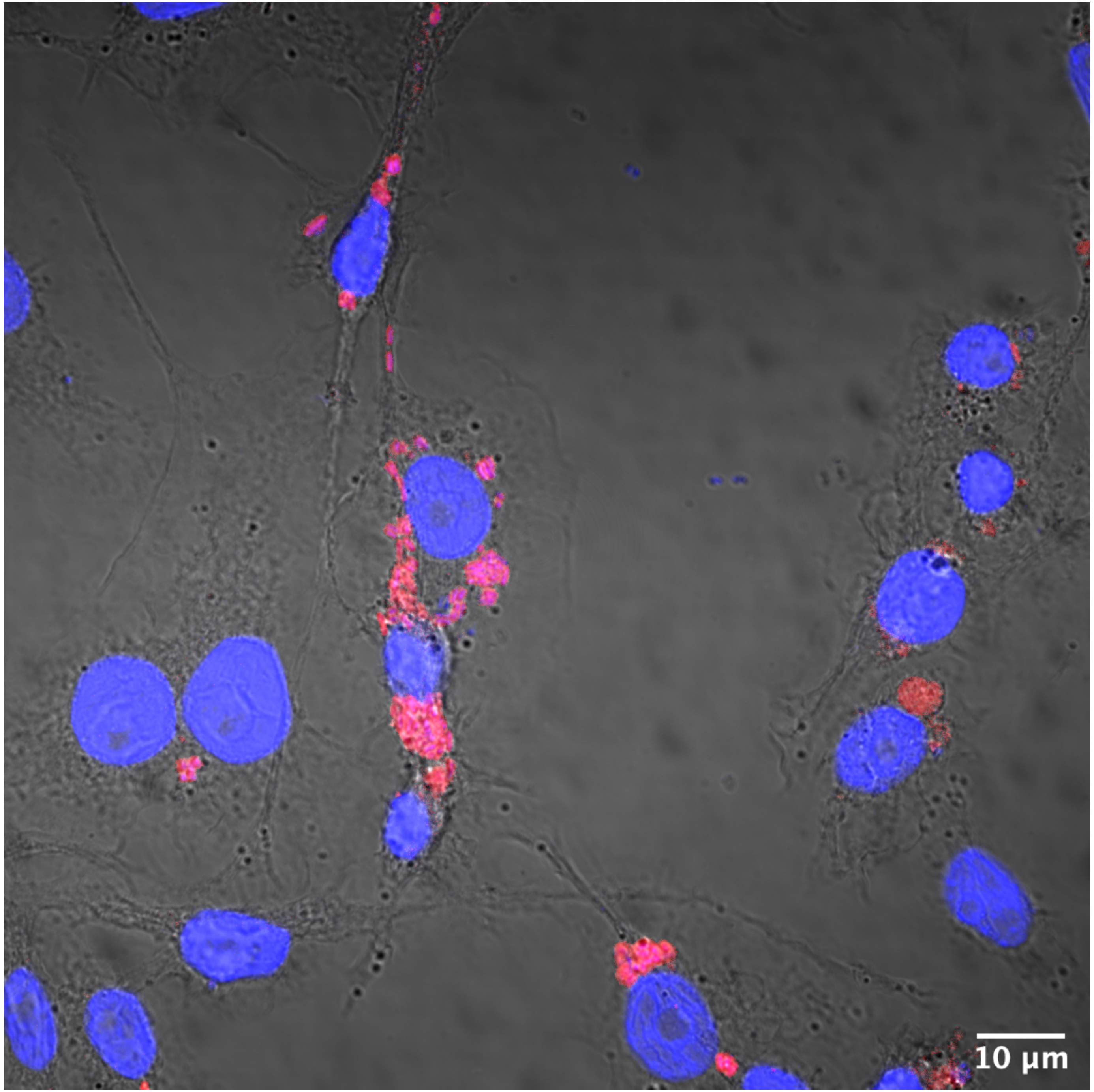

Attachment

uninoculated-
20min

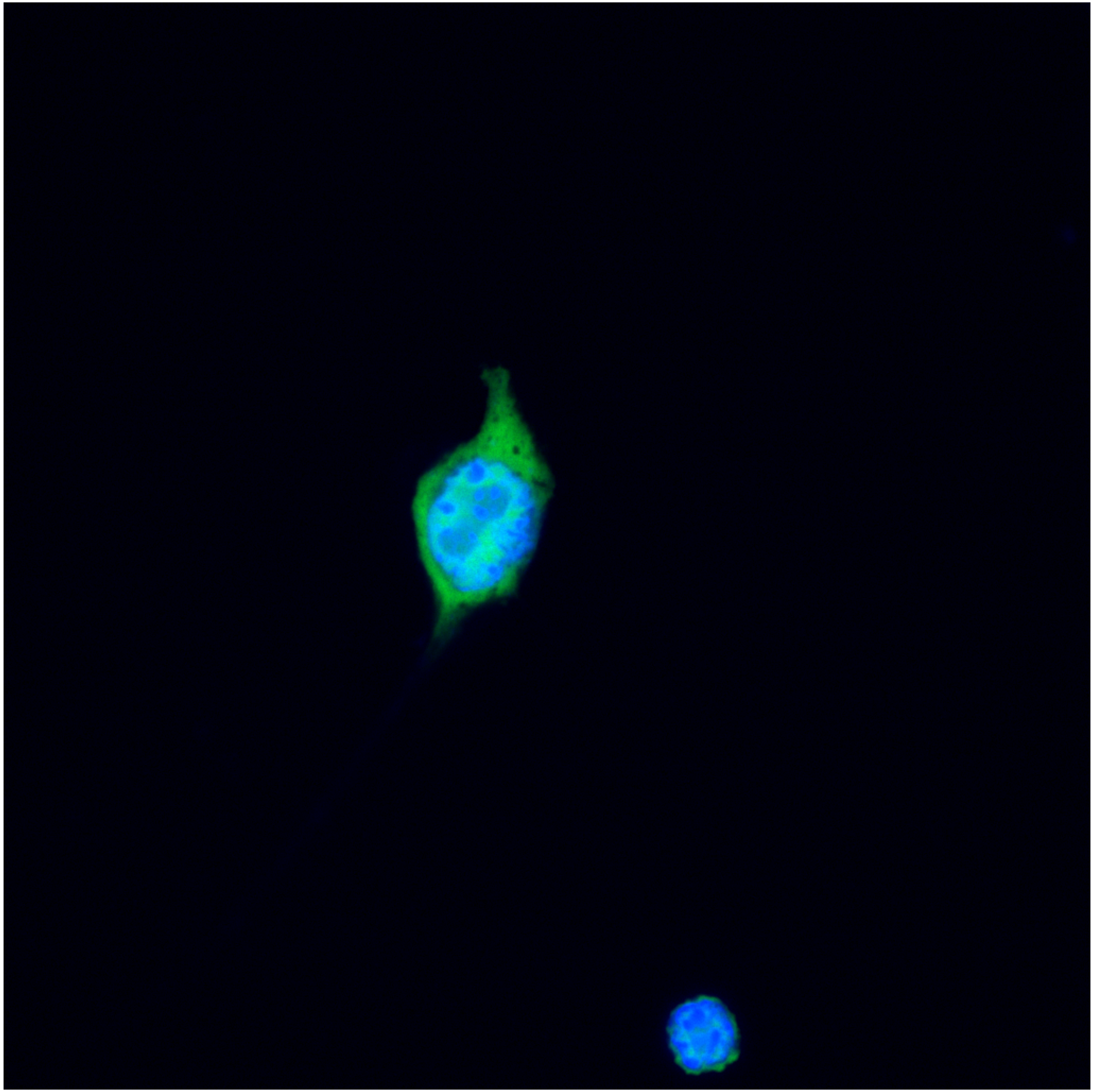

Attachment
uninoculated-2h

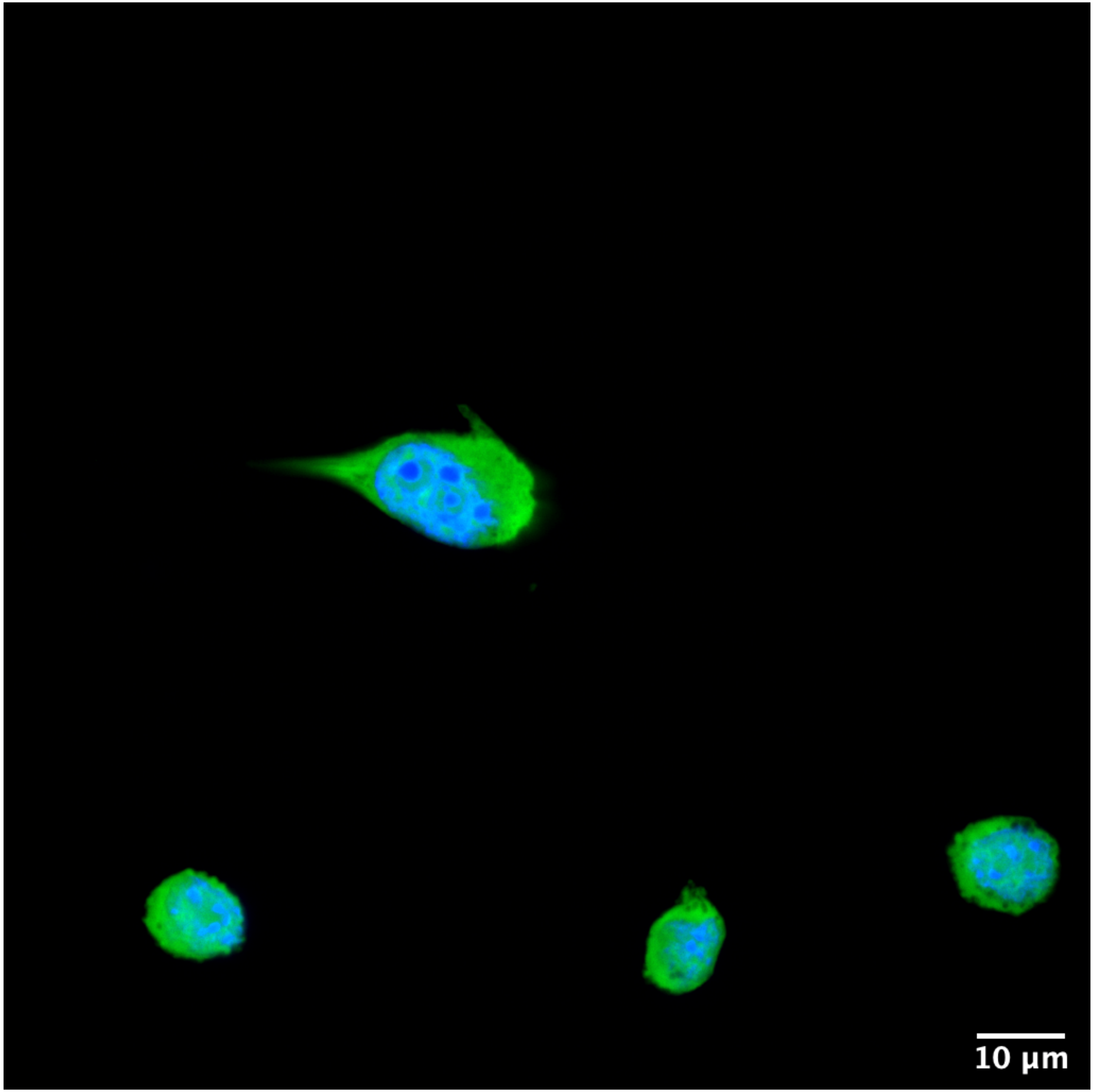

Attachment

Delta erua-fima-
20min

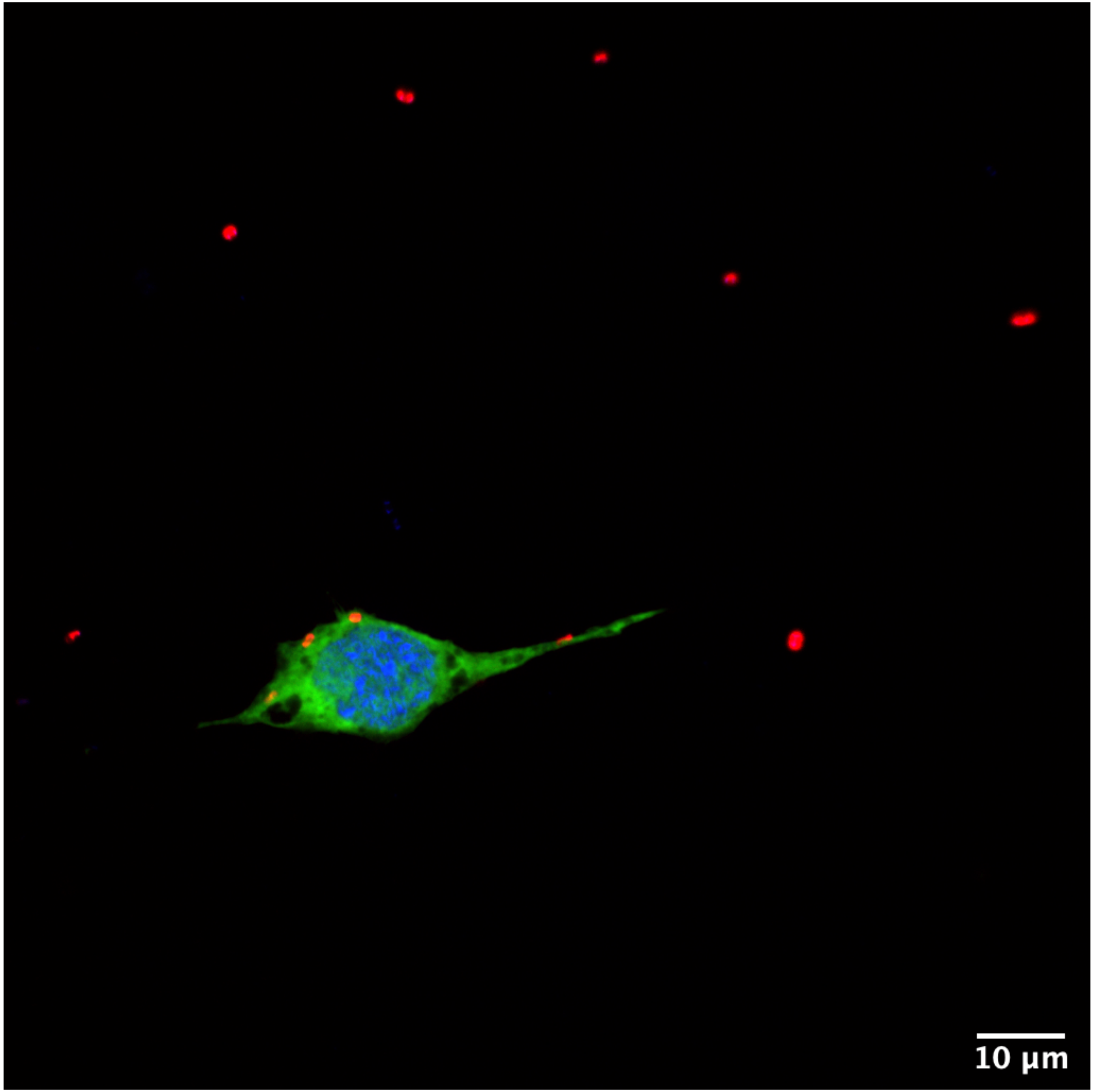

Attachment

Delta erua-fima-
2h

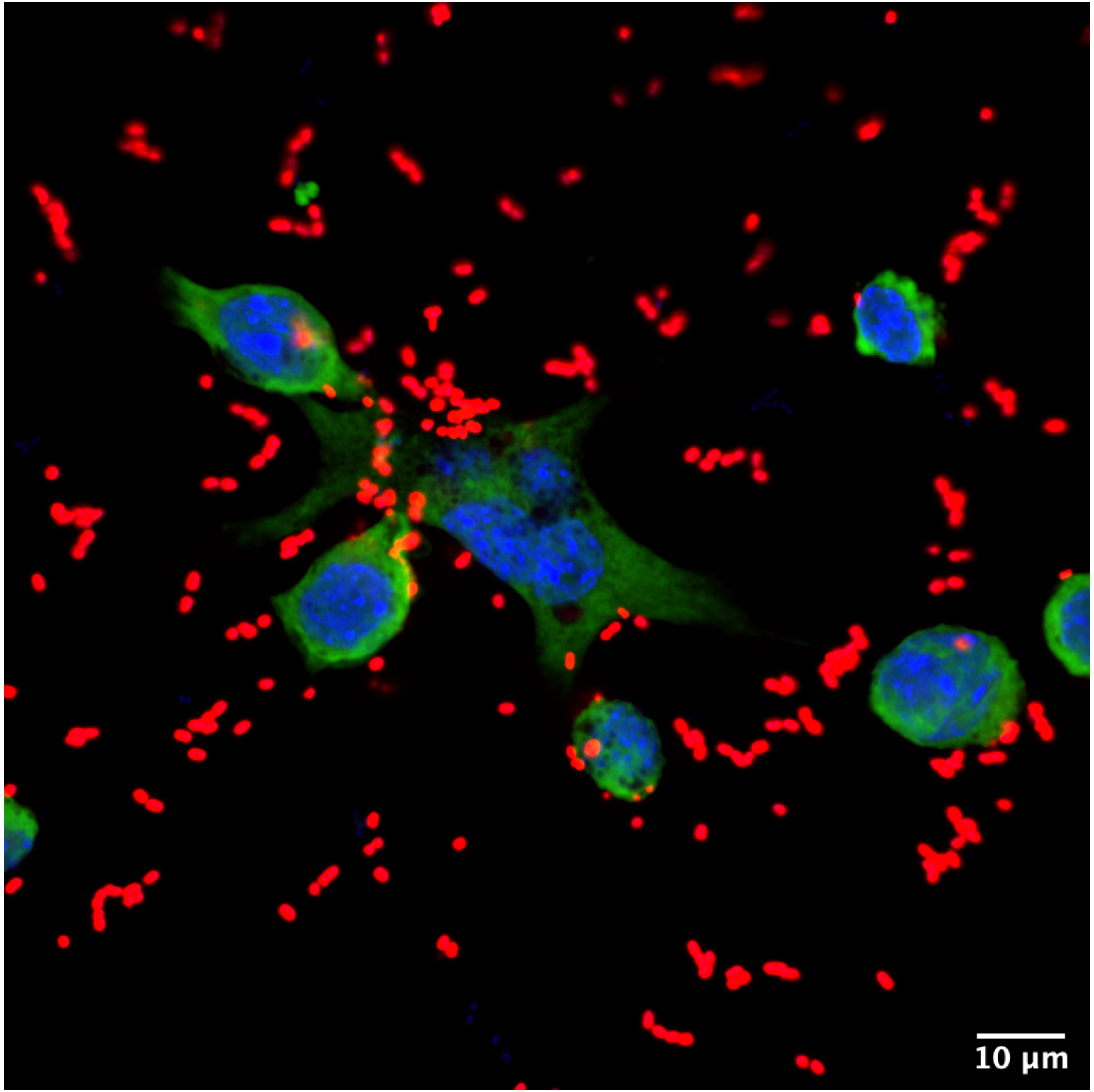

Attachment

Delta erua-flu-
20min

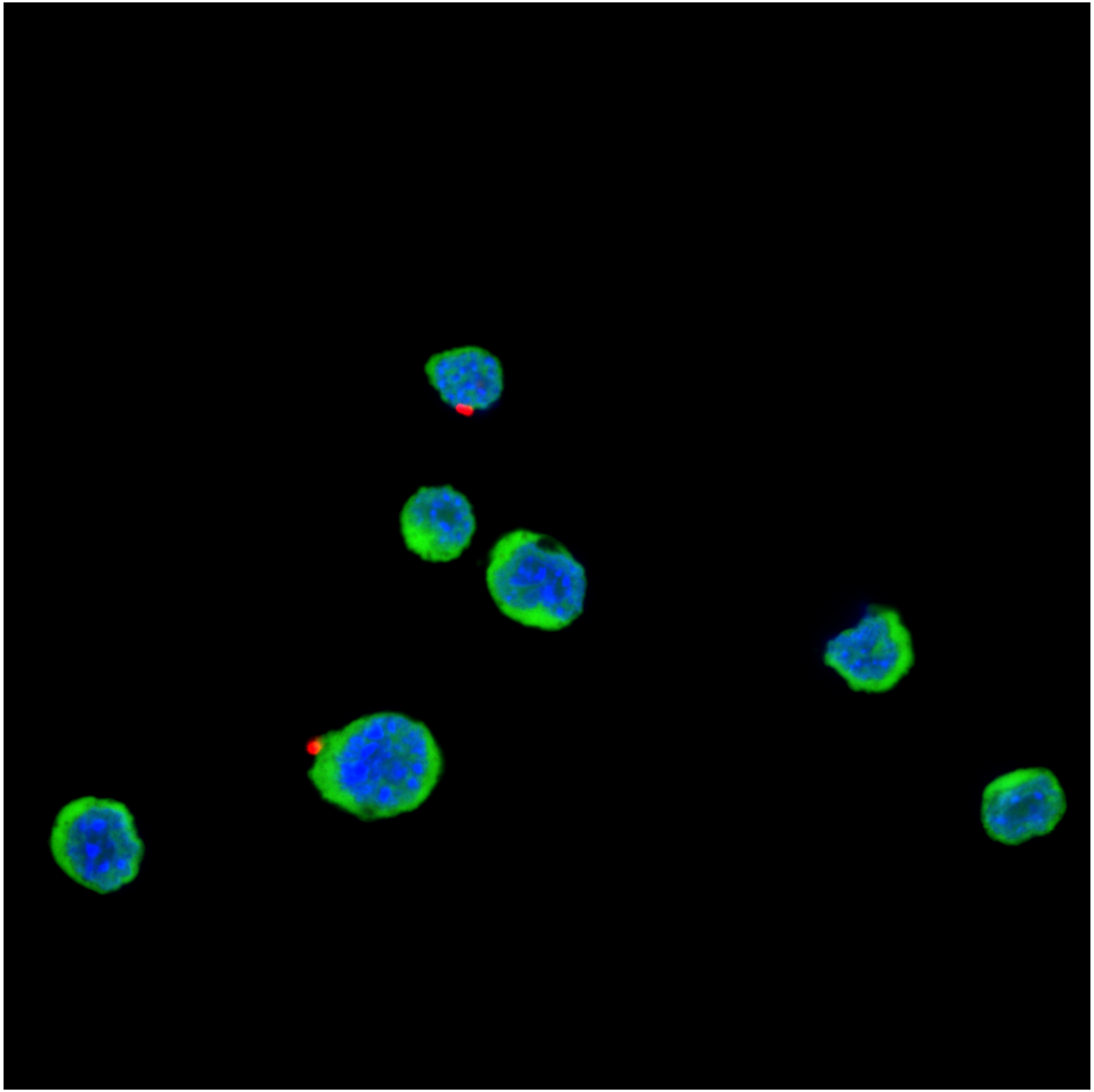

Attachment

Delta erua-flu-
2h

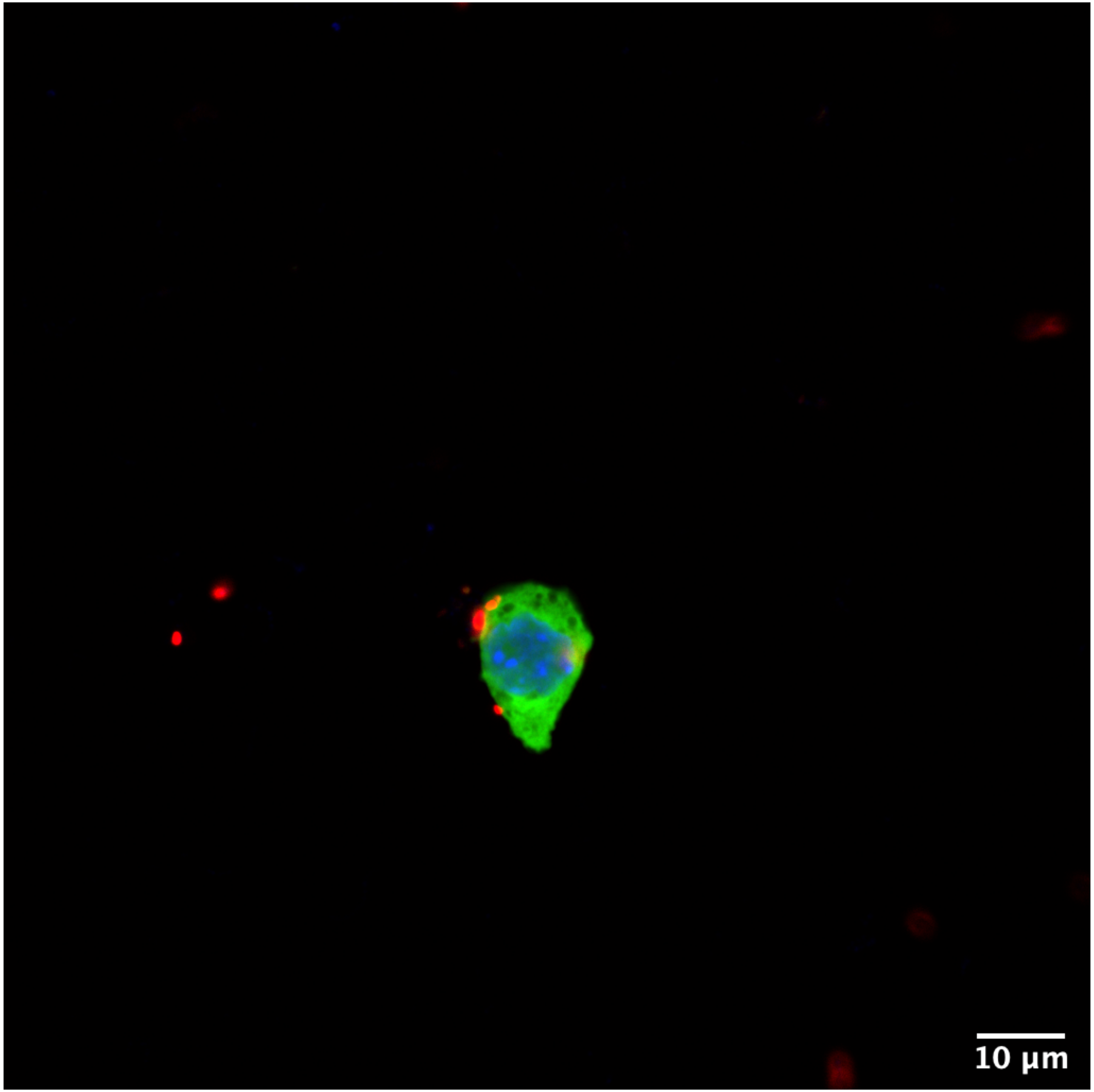

Attachment
WT-flu-20min

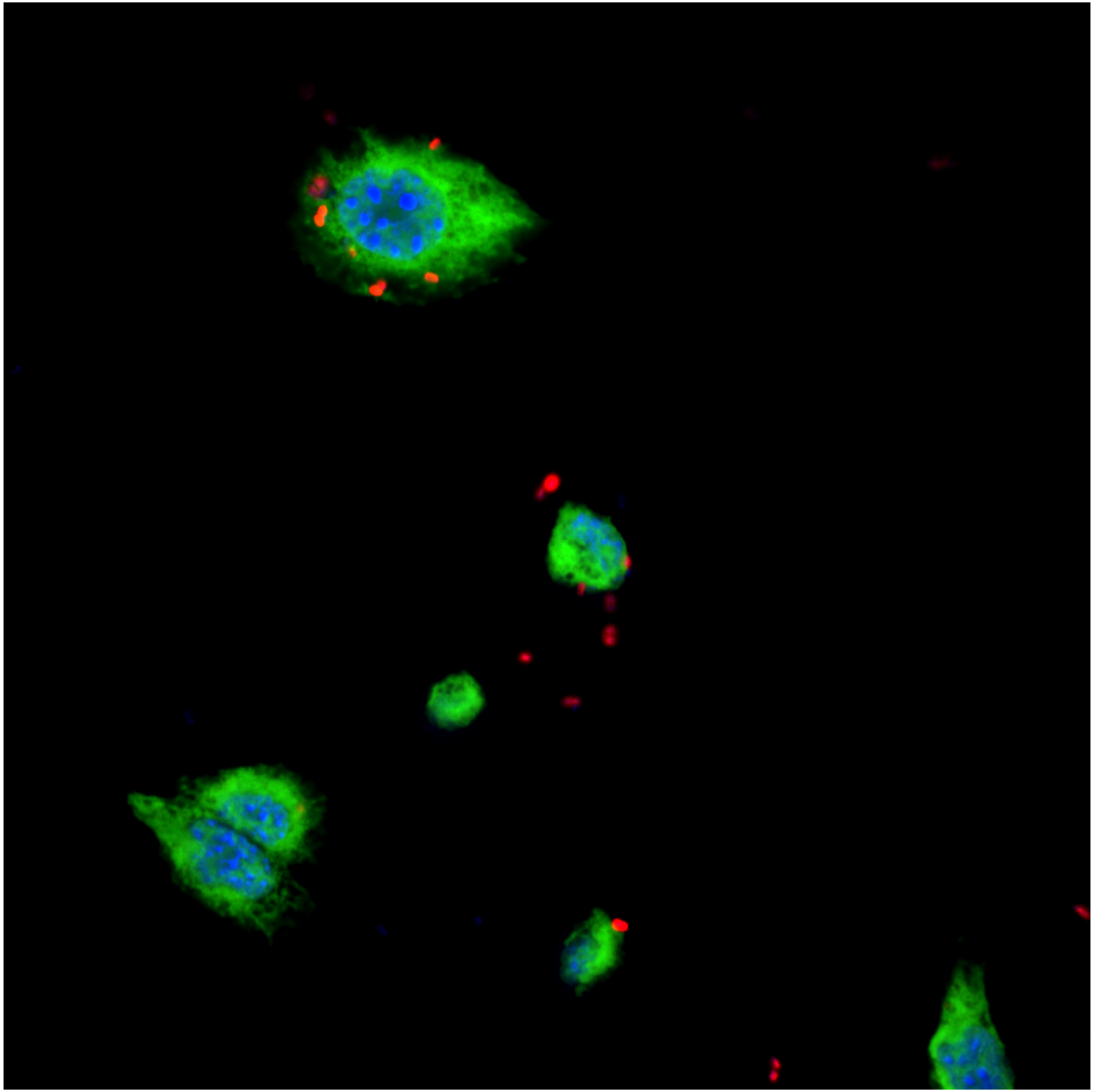

Attachment
WT-flu-2h

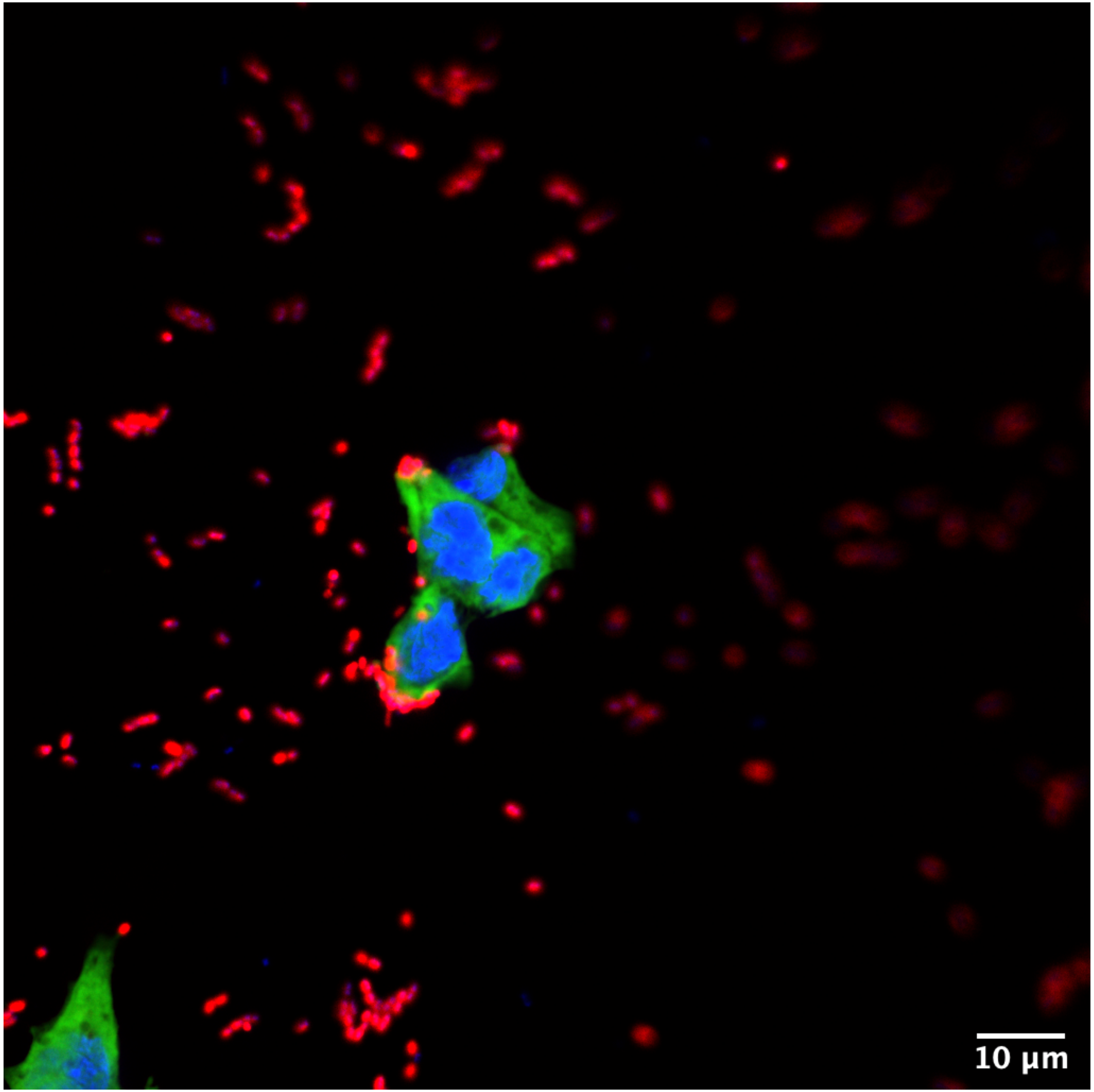

EMSA-1

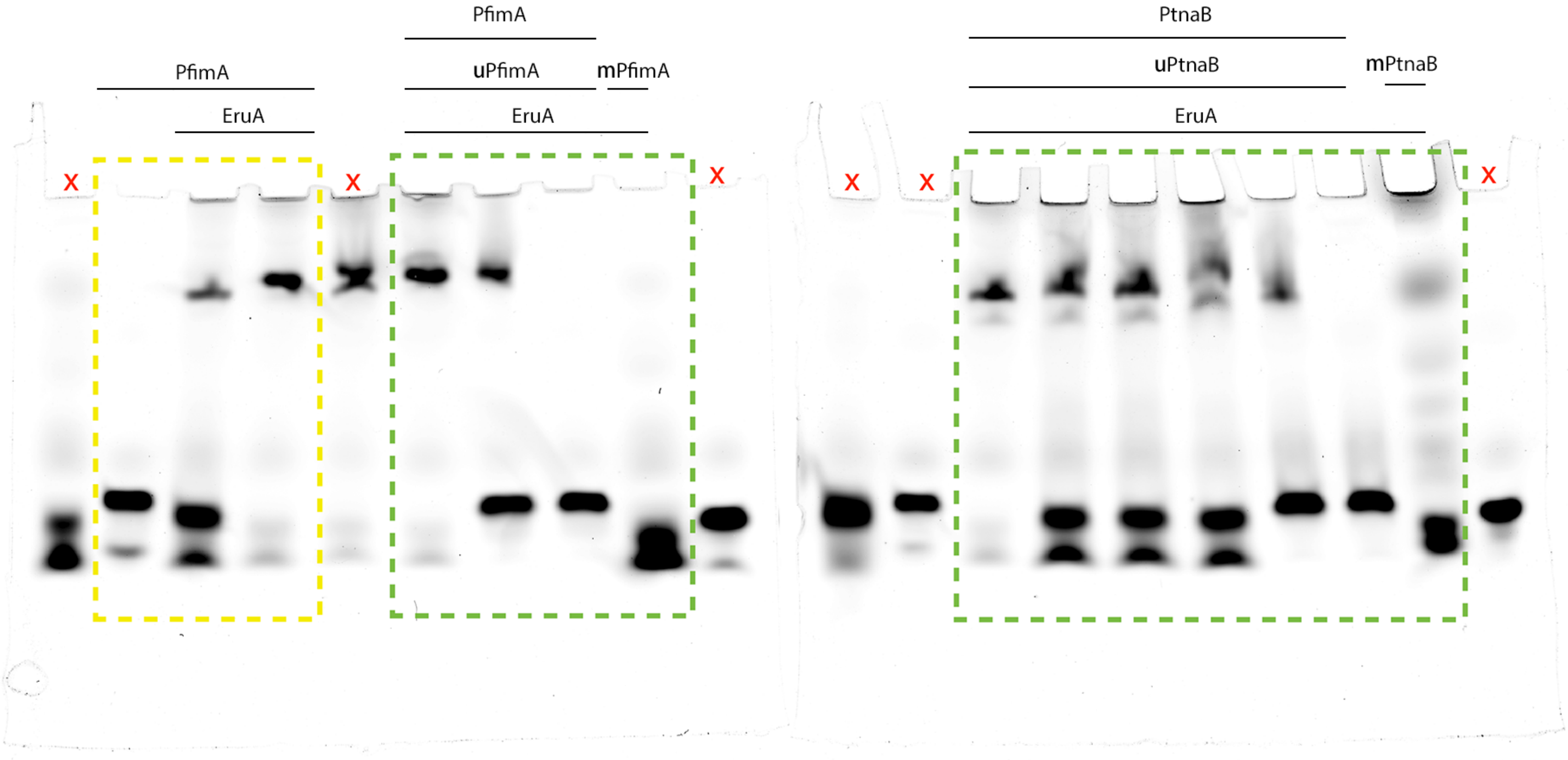
PfimA
EruA
X
PfimA
uPfimA
mPfimA
EruA
X
X
X
PtnaB
uPtnaB
mPtnaB
EruA
X

EMSA-2

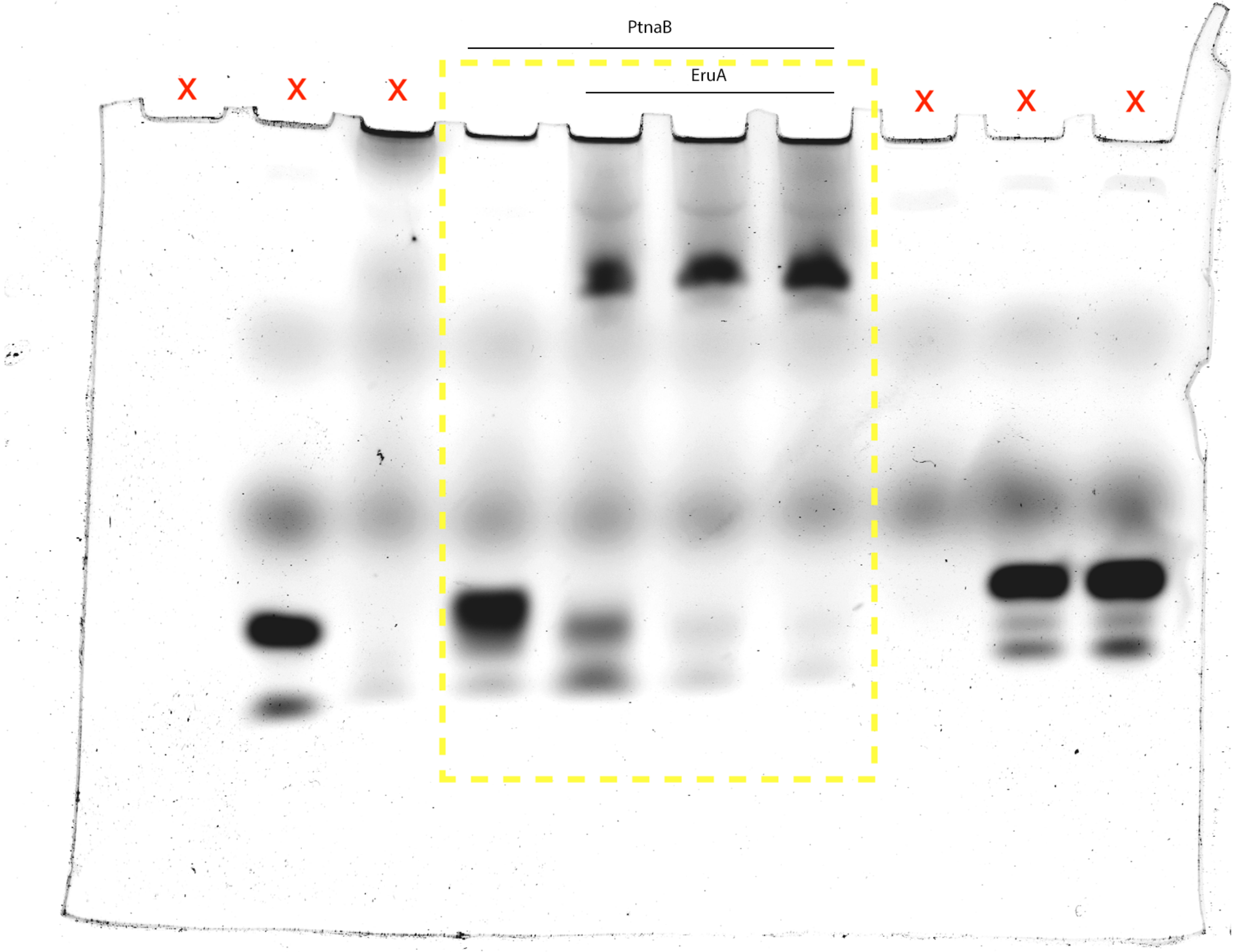

SDS-PAGE

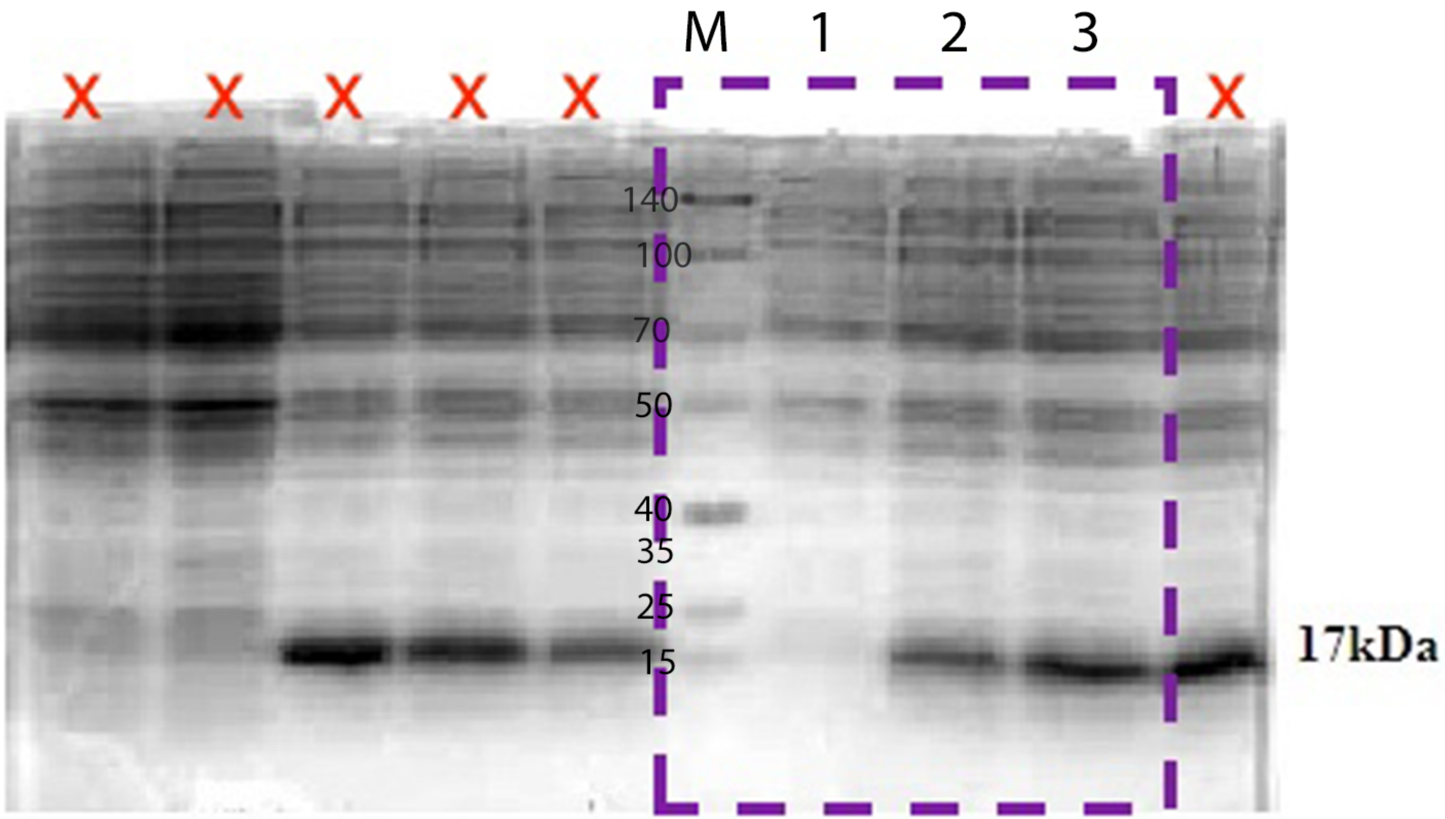

Supplement: Supplementary file 1 [file biomolecules-16-00152-s001.zip › biomolecules-3990550-File S1.original images.pdf]
