# Supplementary material for: EruA, a Regulator of Adherent-Invasive E. coli, Enhances Bacterial Pathogenicity by Promoting Adhesion to Epithelial Cells and Survival Within Macrophages
Source: Biomolecules. 2026 Jan 14;16(1):152. doi: 10.3390/biom16010152 (PMC12839154; doi:10.3390/biom16010152)
Supplement: Supplementary file 1 [file biomolecules-16-00152-s001.zip › table S1.pdf]

Table S1. Variations of the expression of DEGs based on RNA-sequencing.

| Gene ID | Gene alias | Gene size | $log_2$ fold change (eruA_mutant/WT) | *p* value |
|---|---|---|---|---|
| LF82_RS08540 | slyA | 435 | -16.07960978 | 0 |
| LF82_RS06725 | trpE | 1563 | -5.738406092 | 0 |
| LF82_RS12360 | evgS | 3594 | -4.811822006 | 0 |
| LF82_RS19485 | tnaA | 1416 | -4.175652197 | 0 |
| LF82_RS12355 | evgA | 615 | -4.09666578 | 0 |
| LF82_RS08360 | rstA | 720 | -4.081524944 | 0 |
| LF82_RS21910 | lysS | 1518 | -3.929286029 | 0 |
| LF82_RS18430 | mdtF | 3114 | -3.872984468 | 0 |
| LF82_RS18425 | mdtE | 1158 | -3.595811859 | 0 |
| LF82_RS19490 | tnaB | 1248 | -3.506117658 | $3.67x10^{-275}$ |
| LF82_RS19155 | xanP | 1392 | -3.320211495 | 0 |
| LF82_RS13885 | proV | 1203 | -2.975331939 | 0 |
| LF82_RS07625 | bdm | 216 | -2.643044808 | $1.33x10^{-09}$ |
| LF82_RS07720 | fimA | 564 | -2.637390286 | $2.16x10^{-67}$ |
| LF82_RS04110 | artM | 669 | -2.631295685 | $2.43x10^{-207}$ |
| LF82_RS02115 | cyoC | 615 | -2.622220315 | 0 |
| LF82_RS19610 | atpA | 1542 | -2.588714528 | 0 |
| LF82_RS08365 | rstB | 1302 | -2.580844456 | 0 |
| LF82_RS19605 | atpG | 864 | -2.550447911 | 0 |
| LF82_RS19145 | recG | 2082 | -2.528155555 | $1.77x10^{-23}$ |
| LF82_RS03755 | ybhR | 1107 | -2.490154841 | 0 |
| LF82_RS12005 | nuoF | 1338 | -2.475956053 | 0 |
| LF82_RS19600 | atpD | 1383 | -2.453976727 | 0 |
| LF82_RS09815 | cheW | 504 | -2.453507231 | $2.46x10^{-106}$ |
| LF82_RS02110 | cyoD | 330 | -2.422310838 | 0 |
| LF82_RS00620 | aceF | 1893 | -2.403114972 | 0 |
| LF82_RS01610 | betA | 1671 | -2.400084855 | 0 |
| LF82_RS00190 | caiD | 786 | -2.314314911 | $7.90x10^{-4}$ |
| LF82_RS22420 | hflC | 1005 | -2.304998831 | 0 |
| LF82_RS19595 | atpC | 420 | -2.293439676 | 0 |
| LF82_RS04670 | hyaB | 1794 | -2.283390963 | $1.16x10^{-243}$ |
| LF82_RS15570 | glcB | 2172 | -2.262088313 | 0 |
| LF82_RS19615 | atpH | 534 | -2.250947345 | 0 |
| LF82_RS12010 | nuoE | 501 | -2.241145773 | 0 |
| LF82_RS09800 | cheB | 1050 | -2.225266577 | $2.85x10^{-54}$ |
| LF82_RS02105 | cyoE | 891 | -2.219375816 | 0 |
| LF82_RS09820 | cheA | 1965 | -2.210702313 | $1.82x10^{-273}$ |
| LF82_RS03400 | sdhB | 717 | -2.206160141 | 0 |

| | | | | |
|---|---|---|---|---|
| LF82_RS13890 | proW | 1065 | -2.178691506 | $3.73x10^{-147}$ |
| LF82_RS18340 | slp | 567 | -2.155384197 | 0 |
| LF82_RS11855 | nudI | 426 | -2.154536217 | $8.38x10^{-47}$ |
| LF82_RS18395 | yhiD | 648 | -2.137597102 | $8.41x10^{-181}$ |
| LF82_RS13895 | proX | 993 | -2.110785245 | $3.25x10^{-282}$ |
| LF82_RS04715 | cspH | 213 | -2.109637149 | $4.085x10^{-4}$ |
| LF82_RS11605 | napB | 450 | -2.095964029 | $7.56x10^{-14}$ |
| LF82_RS13050 | pepB | 1284 | -2.079373153 | 0 |
| LF82_RS19860 | wecC | 1263 | -2.061268734 | $3.15x10^{-288}$ |
| LF82_RS04195 | ybjX | 957 | -2.055783358 | 0 |
| LF82_RS26175 | ynfU | 171 | -2.007692438 | $5.87x10^{-05}$ |
| LF82_RS19865 | rffG | 1068 | -2.002429958 | $4.20x10^{-168}$ |
| LF82_RS16215 | fadH | 2019 | 2.007867575 | 0 |
| LF82_RS22755 | nrdG | 465 | 2.00945686 | $3.76x10^{-65}$ |
| LF82_RS15900 | mdaB | 582 | 2.010019332 | $4.20x10^{-214}$ |
| LF82_RS21860 | yjdF | 651 | 2.01427245 | $6.44x10^{-25}$ |
| LF82_RS18535 | bcsQ | 753 | 2.024660637 | $4.00x10^{-206}$ |
| LF82_RS02205 | decR | 459 | 2.027735517 | $1.04x10^{-141}$ |
| LF82_RS01845 | sbmA | 1221 | 2.03030074 | $2.84x10^{-144}$ |
| LF82_RS02920 | citC | 1059 | 2.03170886 | $7.53x10^{-3}$ |
| LF82_RS06165 | ymgC | 249 | 2.037768057 | $3.12x10^{-11}$ |
| LF82_RS07635 | dosP | 2400 | 2.041136351 | $4.51x10^{-117}$ |
| LF82_RS14945 | mocA | 579 | 2.049497692 | $2.08x10^{-30}$ |
| LF82_RS07815 | yneJ | 882 | 2.050642378 | $2.29x10^{-93}$ |
| LF82_RS12730 | eutJ | 837 | 2.053439259 | $2.67x10^{-4}$ |
| LF82_RS12615 | yfeK | 369 | 2.058727757 | $3.75x10^{-16}$ |
| LF82_RS02955 | LF82_RS02955 | 789 | 2.060255591 | $1.07x10^{-229}$ |
| LF82_RS18765 | malS | 2031 | 2.06396705 | $1.33x10^{-186}$ |
| LF82_RS18975 | waaH | 1035 | 2.0670207 | $5.24x10^{-113}$ |
| LF82_RS11810 | glpC | 1191 | 2.070036978 | $6.23x10^{-45}$ |
| LF82_RS17620 | tsgA | 1182 | 2.073664511 | $1.44x10^{-136}$ |
| LF82_RS19220 | rhuM | 1038 | 2.078920237 | 0 |
| LF82_RS01405 | LF82_RS01405 | 495 | 2.081196083 | 0.01631584 |
| LF82_RS21525 | soxR | 465 | 2.092634041 | $1.34x10^{-66}$ |
| LF82_RS14100 | hycG | 768 | 2.098284796 | $1.67x10^{-28}$ |
| LF82_RS13640 | LF82_RS13640 | 531 | 2.101467639 | $1.54x10^{-4}$ |
| LF82_RS04075 | potF | 1113 | 2.103265208 | 0 |
| LF82_RS18500 | yhjJ | 1497 | 2.114176203 | 0 |
| LF82_RS18790 | yiaK | 999 | 2.118802678 | $3.51x10^{-36}$ |
| LF82_RS02015 | yajD | 348 | 2.132106582 | $1.94x10^{-257}$ |
| LF82_RS12405 | lpxP | 921 | 2.134316093 | 0 |

| | | | | |
|---|---|---|---|---|
| LF82_RS10675 | yeeY | 930 | 2.158743405 | $1.72x10^{-141}$ |
| LF82_RS22170 | LF82_RS22170 | 204 | 2.159647124 | 0.00913896 |
| LF82_RS07370 | LF82_RS07370 | 237 | 2.172355828 | 0.00241542 |
| LF82_RS04945 | LF82_RS04945 | 243 | 2.173791782 | 0.00913896 |
| LF82_RS18800 | LF82_RS18800 | 921 | 2.176465151 | $1.40x10^{-11}$ |
| LF82_RS10280 | ybtP | 1713 | 2.187958924 | $1.63x10^{-26}$ |
| LF82_RS04245 | csy3 | 1002 | 2.193109191 | $2.03x10^{-231}$ |
| LF82_RS25360 | LF82_RS25360 | 316 | 2.194209654 | 0.0360406 |
| LF82_RS14380 | LF82_RS14380 | 603 | 2.203399706 | 0.00913896 |
| LF82_RS11355 | cirA | 1992 | 2.204986991 | $9.08x10^{-259}$ |
| LF82_RS14720 | LF82_RS14720 | 825 | 2.205675026 | 0.0360406 |
| LF82_RS06795 | yciX | 168 | 2.220993825 | $8.91x10^{-28}$ |
| LF82_RS09510 | tnpA | 459 | 2.22457711 | $1.02x10^{-33}$ |
| LF82_RS09365 | fadD | 1686 | 2.225767337 | 0 |
| LF82_RS00800 | LF82_RS00800 | 2244 | 2.243041409 | 0 |
| LF82_RS07055 | mpaA | 729 | 2.249702592 | $1.92x10^{-126}$ |
| LF82_RS16285 | yqjA | 663 | 2.250350608 | 0 |
| LF82_RS09490 | mgrB | 144 | 2.256780158 | $7.13x10^{-44}$ |
| LF82_RS02220 | glnK | 339 | 2.257319013 | $1.04x10^{-07}$ |
| LF82_RS10390 | LF82_RS10390 | 1263 | 2.282998577 | $5.82x10^{-135}$ |
| LF82_RS08235 | speG | 561 | 2.286642772 | 0 |
| LF82_RS07695 | ydeQ | 915 | 2.290849784 | $1.28x10^{-06}$ |
| LF82_RS20930 | fabR | 651 | 2.291699698 | 0 |
| LF82_RS19660 | asnA | 993 | 2.3221172 | 0 |
| LF82_RS18615 | eptB | 1692 | 2.330673307 | 0 |
| LF82_RS21985 | LF82_RS21985 | 753 | 2.332983283 | 0.0050602 |
| LF82_RS13840 | alaE | 450 | 2.339799503 | 0 |
| LF82_RS08615 | ydhP | 1170 | 2.340440475 | $1.78x10^{-211}$ |
| LF82_RS06085 | LF82_RS06085 | 279 | 2.353146825 | 0.01967002 |
| LF82_RS14095 | hycH | 411 | 2.353622737 | $3.78x10^{-34}$ |
| LF82_RS22605 | LF82_RS22605 | 792 | 2.358453971 | $5.39x10^{-09}$ |
| LF82_RS10340 | LF82_RS10340 | 1053 | 2.359310317 | $2.54x10^{-10}$ |
| LF82_RS02270 | tomB | 375 | 2.367908903 | $7.36x10^{-159}$ |
| LF82_RS17095 | yhdU | 180 | 2.370698535 | 0.0027732 |
| LF82_RS23325 | fhuF | 789 | 2.370760741 | 0 |
| LF82_RS06665 | tonB | 720 | 2.376358825 | 0 |
| LF82_RS07480 | LF82_RS07480 | 480 | 2.379606669 | $8.56x10^{-122}$ |
| LF82_RS05175 | phoH | 789 | 2.382623786 | 0 |
| LF82_RS02800 | entC | 1176 | 2.39933726 | 0 |
| LF82_RS06135 | ycgX | 405 | 2.400340988 | $1.13x10^{-4}$ |
| LF82_RS25795 | LF82_RS25795 | 1348 | 2.42204969 | 0 |

| | | | | |
|---|---|---|---|---|
| LF82_RS15460 | LF82_RS15460 | 1158 | 2.465122731 | $6.92x10^{-08}$ |
| LF82_RS18795 | yiaL | 465 | 2.486709793 | $2.40x10^{-07}$ |
| LF82_RS15590 | glcD | 1500 | 2.494263324 | 0 |
| LF82_RS08840 | ydiT | 294 | 2.504994196 | 0.0105989 |
| LF82_RS20055 | rhtB | 621 | 2.517942443 | $1.69x10^{-34}$ |
| LF82_RS16420 | prlF | 336 | 2.519094937 | $2.97x10^{-183}$ |
| LF82_RS17460 | LF82_RS17460 | 462 | 2.522678222 | 0.0105989 |
| LF82_RS02640 | ompT | 954 | 2.525529587 | $2.53x10^{-81}$ |
| LF82_RS00080 | LF82_RS00080 | 762 | 2.527931556 | $3.41x10^{-05}$ |
| LF82_RS01290 | fadE | 2445 | 2.531674202 | 0 |
| LF82_RS15930 | LF82_RS15930 | 819 | 2.540559565 | $1.36x10^{-129}$ |
| LF82_RS05250 | csgD | 651 | 2.540684076 | $1.50x10^{-217}$ |
| LF82_RS02750 | fepA | 2250 | 2.551483863 | 0 |
| LF82_RS04250 | cas6f | 555 | 2.555239375 | $2.43x10^{-82}$ |
| LF82_RS25575 | LF82_RS25575 | 186 | 2.566054038 | $8.11x10^{-4}$ |
| LF82_RS08230 | ynfB | 342 | 2.573101074 | 0 |
| LF82_RS26305 | LF82_RS26305 | 229 | 2.59164928 | 0 |
| LF82_RS18685 | yiaG | 291 | 2.604927388 | 0 |
| LF82_RS07890 | dgcZ | 891 | 2.613953718 | 0 |
| LF82_RS09370 | yeaY | 582 | 2.62328369 | 0 |
| LF82_RS07460 | LF82_RS07460 | 285 | 2.62624224 | $3.33x10^{-05}$ |
| LF82_RS20270 | fadB | 2190 | 2.650105436 | 0 |
| LF82_RS00060 | yaaI | 405 | 2.658340163 | $1.63x10^{-10}$ |
| LF82_RS22815 | LF82_RS22815 | 1404 | 2.658800729 | $2.98x10^{-147}$ |
| LF82_RS25445 | LF82_RS25445 | 399 | 2.660877478 | $6.53x10^{-05}$ |
| LF82_RS18025 | yhhZ | 1179 | 2.672425342 | $6.53x10^{-05}$ |
| LF82_RS10700 | hisD | 1305 | 2.692833224 | 0 |
| LF82_RS02500 | hyi | 777 | 2.700439718 | $6.25x10^{-09}$ |
| LF82_RS21365 | malM | 921 | 2.714478716 | 0 |
| LF82_RS13725 | LF82_RS13725 | 189 | 2.737416366 | 0.0426822 |
| LF82_RS06600 | LF82_RS06600 | 1048 | 2.748180671 | $7.19x10^{-49}$ |
| LF82_RS18370 | hutW | 1338 | 2.749505891 | $9.28x10^{-153}$ |
| LF82_RS01230 | LF82_RS01230 | 666 | 2.757429697 | 0.0426822 |
| LF82_RS05895 | rusA | 363 | 2.776780108 | 0.00298186 |
| LF82_RS21345 | malE | 1191 | 2.779152208 | 0 |
| LF82_RS11100 | yehA | 1035 | 2.785261151 | $1.86x10^{-05}$ |
| LF82_RS19755 | maoP | 339 | 2.795790048 | 0 |
| LF82_RS16940 | aaeX | 204 | 2.803700623 | $9.87x10^{-06}$ |
| LF82_RS02265 | hha | 219 | 2.806457185 | $3.08x10^{-169}$ |
| LF82_RS10670 | plaP | 1359 | 2.839183359 | 0 |
| LF82_RS12385 | yfdX | 633 | 2.839359977 | $1.86x10^{-13}$ |

| | | | | |
|---|---|---|---|---|
| LF82_RS21330 | psiE | 411 | 2.879930892 | $1.35x10^{-139}$ |
| LF82_RS08955 | ydiY | 759 | 2.880850189 | $4.66x10^{-249}$ |
| LF82_RS12030 | lrhA | 939 | 2.889546876 | 0 |
| LF82_RS14045 | norR | 1515 | 2.890146573 | 0 |
| LF82_RS21770 | phnE | 780 | 2.908812908 | $1.96x10^{-08}$ |
| LF82_RS02680 | cusB | 1224 | 2.917228534 | $5.94x10^{-44}$ |
| LF82_RS06160 | ariR | 267 | 2.933184624 | $3.92x10^{-112}$ |
| LF82_RS26380 | yqgG | 144 | 2.943583699 | 0.021934 |
| LF82_RS13875 | nrdE | 2145 | 2.953417367 | $2.22x10^{-303}$ |
| LF82_RS03785 | LF82_RS03785 | 201 | 2.966103581 | 0.021934 |
| LF82_RS02755 | fes | 1203 | 2.967862222 | 0 |
| LF82_RS09610 | yebE | 660 | 2.986162108 | 0 |
| LF82_RS08555 | ydhK | 2013 | 2.988454999 | $8.01x10^{-301}$ |
| LF82_RS17475 | bfd | 195 | 2.993561163 | 0 |
| LF82_RS04955 | LF82_RS04955 | 423 | 3.013135402 | $8.11x10^{-4}$ |
| LF82_RS06100 | sitC | 858 | 3.027918399 | $5.88x10^{-122}$ |
| LF82_RS01795 | LF82_RS01795 | 672 | 3.03562391 | 0.021934 |
| LF82_RS21335 | malG | 891 | 3.047401258 | 0 |
| LF82_RS03145 | asnB | 1665 | 3.09006945 | 0 |
| LF82_RS04005 | ybjG | 597 | 3.097795433 | 0 |
| LF82_RS19115 | yicG | 618 | 3.10388965 | $1.64x10^{-209}$ |
| LF82_RS09875 | ftnB | 504 | 3.179823485 | 0 |
| LF82_RS15400 | speC | 2136 | 3.200773083 | 0 |
| LF82_RS21360 | lamB | 1341 | 3.211585904 | 0 |
| LF82_RS13680 | LF82_RS13680 | 651 | 3.222392421 | 0.01115562 |
| LF82_RS10695 | hisG | 900 | 3.233835351 | 0 |
| LF82_RS16565 | mtr | 1245 | 3.261666775 | 0 |
| LF82_RS19775 | ilvM | 264 | 3.266298423 | $2.29x10^{-15}$ |
| LF82_RS13865 | nrdH | 246 | 3.282677539 | $2.99x10^{-67}$ |
| LF82_RS08370 | tus | 930 | 3.287910221 | 0 |
| LF82_RS18355 | chuA | 1983 | 3.329993345 | 0 |
| LF82_RS07715 | LF82_RS07715 | 711 | 3.342933953 | $2.99x10^{-23}$ |
| LF82_RS22775 | treB | 1422 | 3.388713271 | 0 |
| LF82_RS19570 | pstA | 891 | 3.419842017 | 0 |
| LF82_RS01445 | ykgJ | 330 | 3.488218313 | $5.30x10^{-76}$ |
| LF82_RS04330 | ycaD | 1149 | 3.489412223 | 0 |
| LF82_RS02495 | gcl | 1782 | 3.496679003 | $1.37x10^{-195}$ |
| LF82_RS18365 | chuT | 915 | 3.497503768 | $1.94x10^{-284}$ |
| LF82_RS05930 | LF82_RS05930 | 534 | 3.539158811 | $1.42x10^{-05}$ |
| LF82_RS04240 | csy2 | 924 | 3.539671056 | $2.07x10^{-302}$ |
| LF82_RS04235 | csy1 | 1308 | 3.578570078 | 0 |

| | | | | |
|---|---|---|---|---|
| LF82_RS06105 | sitB | 828 | 3.609521393 | 0 |
| LF82_RS19770 | ilvG | 1647 | 3.610970676 | 0 |
| LF82_RS20015 | corA | 951 | 3.622298269 | 0 |
| LF82_RS02675 | cusF | 333 | 3.6283046 | $5.84 \times 10^{-16}$ |
| LF82_RS07365 | yncJ | 231 | 3.642196136 | $1.58 \times 10^{-26}$ |
| LF82_RS00390 | leuA | 1572 | 3.729640713 | 0 |
| LF82_RS05390 | mdtH | 1209 | 3.742508926 | 0 |
| LF82_RS17100 | acrS | 663 | 3.772589504 | $6.94 \times 10^{-4}$ |
| LF82_RS05835 | LF82_RS05835 | 540 | 3.777607579 | $6.94 \times 10^{-4}$ |
| LF82_RS08425 | malX | 1593 | 3.803495038 | $1.48 \times 10^{-159}$ |
| LF82_RS01500 | LF82_RS01500 | 921 | 3.826277288 | 0 |
| LF82_RS01875 | phoA | 1416 | 3.857222271 | 0 |
| LF82_RS00855 | degP | 1425 | 3.865916236 | 0 |
| LF82_RS06360 | dhaM | 1419 | 3.884744145 | 0 |
| LF82_RS18870 | yibH | 1137 | 3.904189295 | 0 |
| LF82_RS22820 | argF | 1005 | 3.96580508 | $2.19 \times 10^{-149}$ |
| LF82_RS02485 | allA | 483 | 3.97713848 | $1.59 \times 10^{-178}$ |
| LF82_RS06365 | dhaL | 633 | 4.032573813 | 0 |
| LF82_RS06720 | trpD | 1596 | 4.042563163 | 0 |
| LF82_RS18875 | yibI | 363 | 4.095648531 | $1.66 \times 10^{-100}$ |
| LF82_RS21340 | malF | 1545 | 4.111099206 | 0 |
| LF82_RS05575 | fhuE | 2190 | 4.134635233 | 0 |
| LF82_RS10285 | ybtA | 960 | 4.224087461 | 0 |
| LF82_RS23330 | yjjZ | 237 | 4.231098385 | $5.84 \times 10^{-52}$ |
| LF82_RS06370 | dhaK | 1071 | 4.237992232 | 0 |
| LF82_RS22830 | arcA | 1221 | 4.333199207 | $1.80 \times 10^{-213}$ |
| LF82_RS22825 | arcC | 945 | 4.544885061 | $1.15 \times 10^{-149}$ |
| LF82_RS13870 | nrdI | 411 | 4.552511851 | $2.21 \times 10^{-141}$ |
| LF82_RS22785 | mgtA | 2697 | 4.56089265 | 0 |
| LF82_RS08550 | ydhJ | 858 | 4.678078271 | 0 |
| LF82_RS08545 | ydhI | 237 | 4.680827472 | 0 |
| LF82_RS01495 | LF82_RS01495 | 1143 | 4.759953255 | 0 |
| LF82_RS01960 | phoB | 690 | 4.776845876 | 0 |
| LF82_RS01965 | phoR | 1296 | 4.905170215 | 0 |
| LF82_RS19575 | pstC | 960 | 5.11768889 | 0 |
| LF82_RS03305 | kdpB | 2049 | 5.124987252 | 0 |
| LF82_RS21355 | malK | 1116 | 5.30752503 | 0 |
| LF82_RS06110 | sitA | 915 | 5.430796888 | 0 |
| LF82_RS21775 | phnD | 1017 | 5.540552551 | $1.74 \times 10^{-176}$ |
| LF82_RS02670 | cusC | 1383 | 5.899951915 | $3.75 \times 10^{-124}$ |
| LF82_RS15470 | LF82_RS15470 | 1251 | 6.022367813 | 0.0089524 |

| | | | | |
|---|---|---|---|---|
| LF82_RS01380 | phoE | 1056 | 6.106035382 | 0 |
| LF82_RS19580 | pstS | 1041 | 6.397772529 | 0 |
| LF82_RS21780 | phnC | 789 | 6.439458188 | 0 |
| LF82_RS03310 | kdpA | 1674 | 7.461210162 | 0 |
| LF82_RS16920 | yhcN | 264 | 7.77463688 | 0 |
| LF82_RS06255 | LF82_RS06255 | 324 | 9.28771238 | 0.00190884 |
| LF82_RS07640 | LF82_RS07640 | 165 | 13.14752294 | $1.85x10^{-05}$ |
| LF82_RS26080 | LF82_RS26080 | 135 | 13.47231006 | 0.0193876 |

*P v*alues shown are significant.
