# Supplementary figures and images for: EruA, a Regulator of Adherent-Invasive E. coli, Enhances Bacterial Pathogenicity by Promoting Adhesion to Epithelial Cells and Survival Within Macrophages
Source: Biomolecules. 2026 Jan 14;16(1):152. doi: 10.3390/biom16010152 (PMC12839154; doi:10.3390/biom16010152)

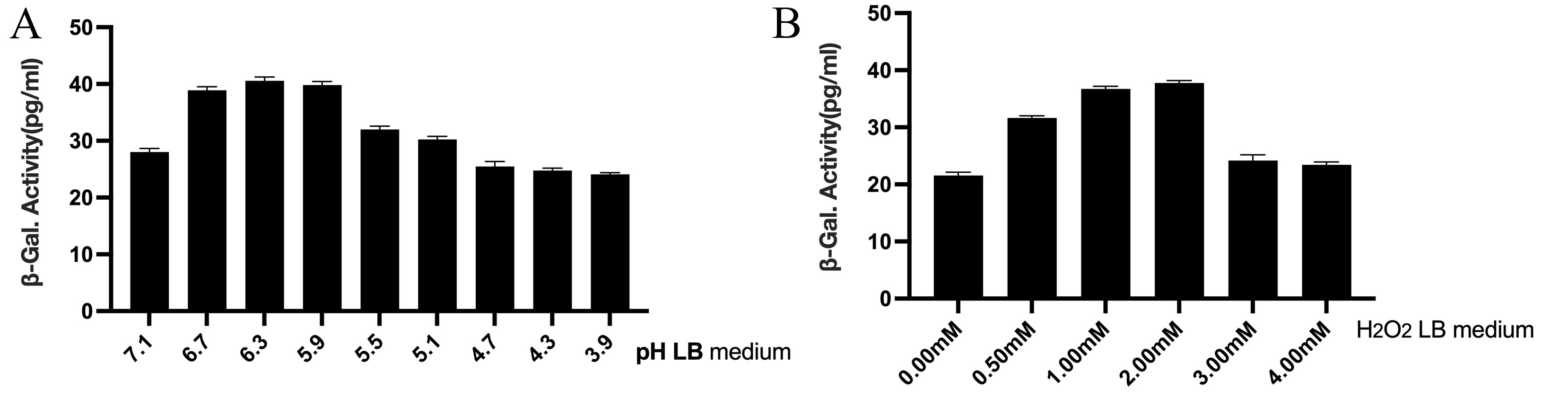

Supplement: Supplementary file 1 [file biomolecules-16-00152-s001.zip › Figure S1.jpg]

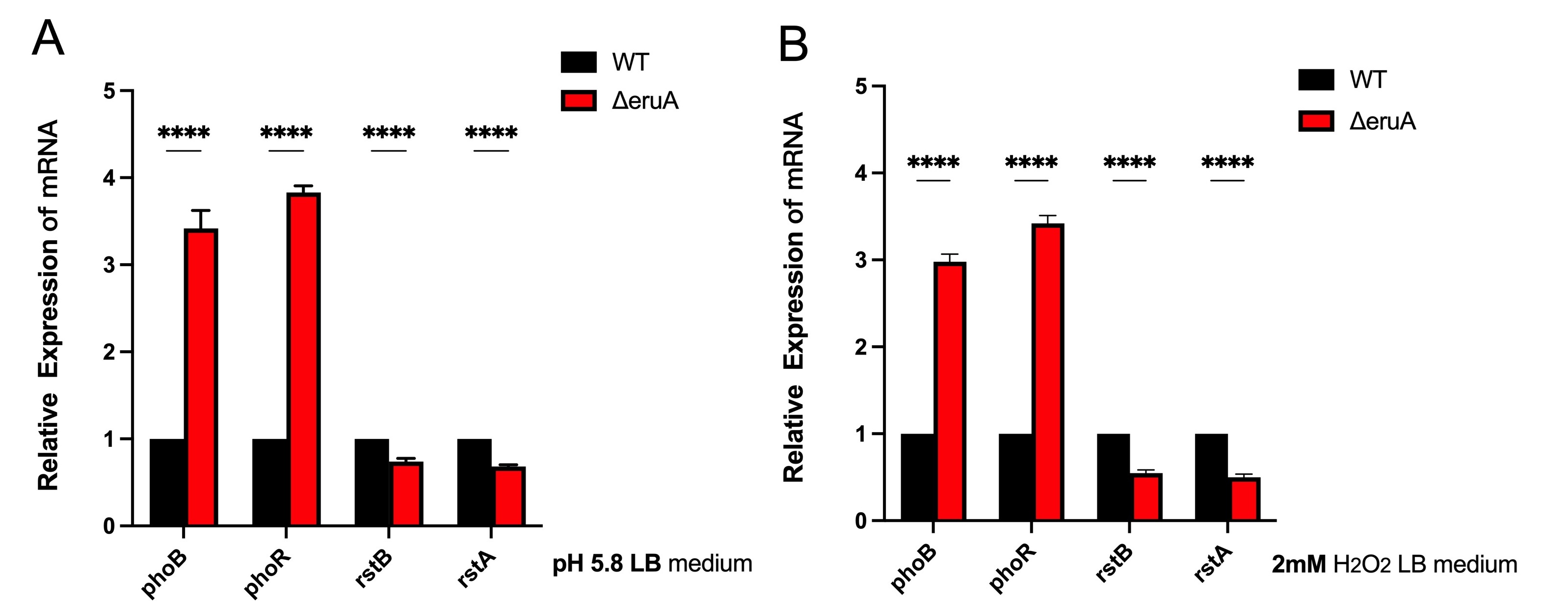

Supplement: Supplementary file 1 [file biomolecules-16-00152-s001.zip › Figure S2.jpg]

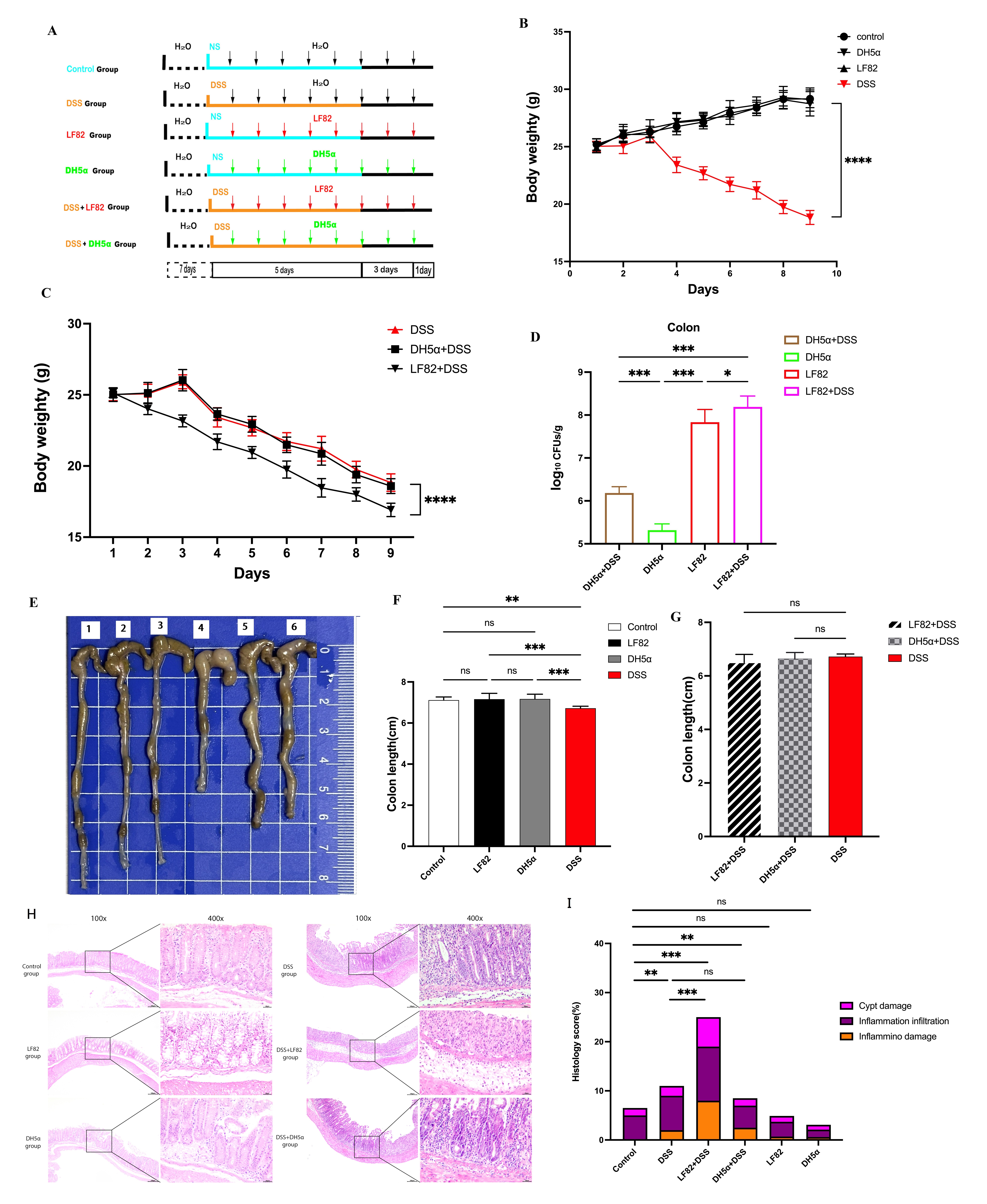

Supplement: Supplementary file 1 [file biomolecules-16-00152-s001.zip › Figure S3.jpg]

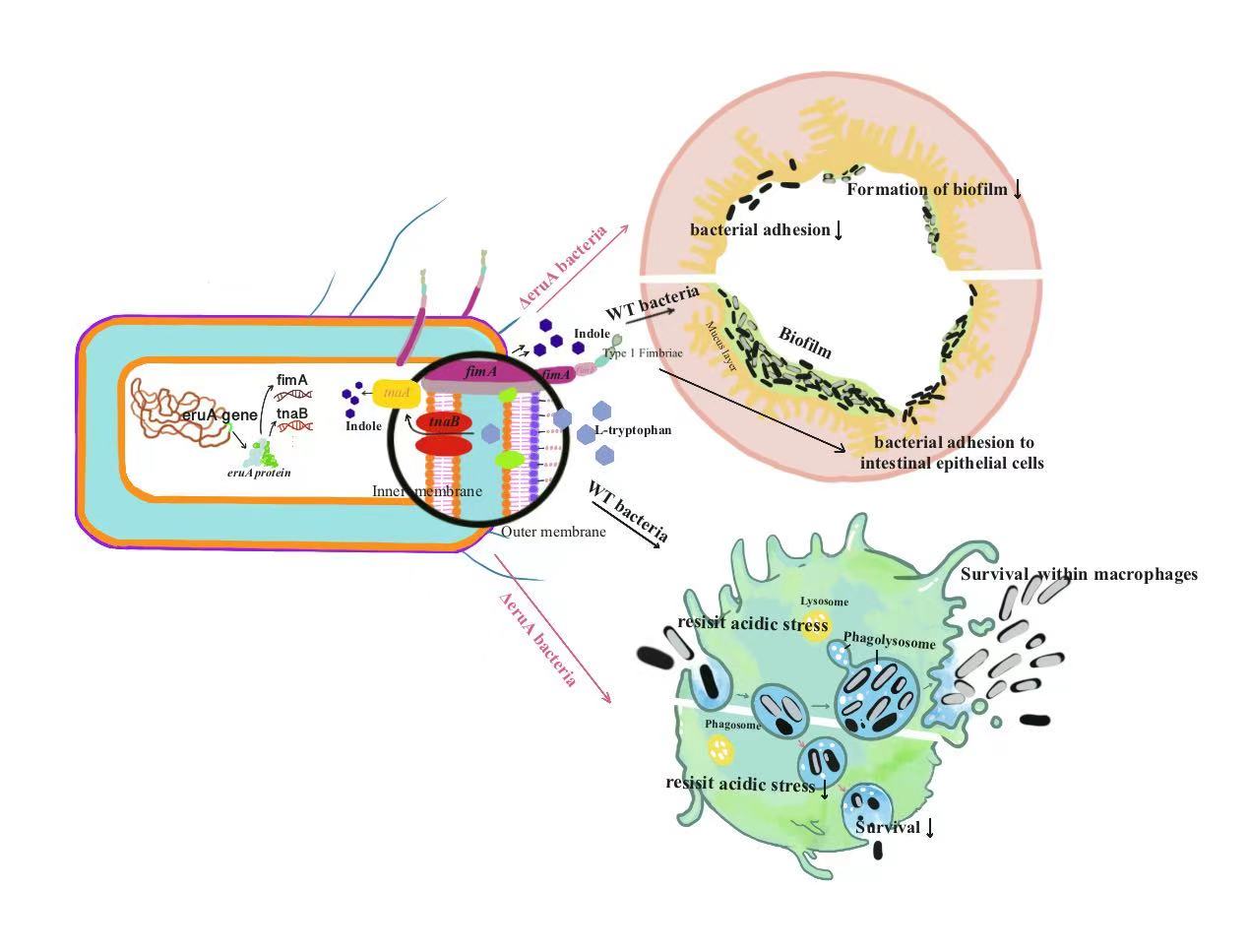

Supplement: Supplementary file 1 [file biomolecules-16-00152-s001.zip › Figure S4.jpg]
